# UPLC-ESI-TOF-MS Profiling of Metabolome Alterations in Barley (*Hordeum vulgare* L.) Leaves Induced by *Bipolaris sorokiniana*

**DOI:** 10.1021/acs.jafc.5c05419

**Published:** 2025-09-18

**Authors:** Lisa Kurzweil, Timo D. Stark, Karina Hille, Felix Hoheneder, Jana Mrtva, Johann Hausladen, Miriam Lenk, Mohammed Saddik Motawia, Nicole Strittmatter, A. Corina Vlot, Klaus Pillen, Mette So̷rensen, Birger L. Mo̷ller, Ralph Hückelhoven, Corinna Dawid

**Affiliations:** † Professorship for Functional Phytometabolomics, TUM School of Life Sciences, 9184 Technical University of Munich, Lise-Meitner-Str. 34, 85354 Freising, Germany; ‡ Chair of Food Chemistry and Molecular Sensory Science, TUM School of Life Sciences, 9184 Technical University of Munich, Lise-Meitner-Str. 34, 85354 Freising, Germany; § Chair of Phytopathology, TUM School of Life Sciences, 9184 Technical University of Munich, Emil-Ramann-Str. 2, 85354 Freising, Germany; ∥ Plant Technology Center, TUM School of Life Sciences, 9184 Technical University of Munich, Dürnast 9, 85354 Freising, Germany; ⊥ Institute of Biochemical Plant Pathology, Helmholtz Zentrum München, Ingolstädter Landstraße 1, 85764 Neuherberg, Germany; # Department of Plant and Environmental Sciences, 4321 University of Copenhagen, Thorvaldsensvej 40, 1871 Frederiskberg C, Copenhagen Denmark; ∇ 636987 Novo Nordisk Pharmatech, Ko̷benhavnsvej 216, 4600 Ko̷ge, Copenhagen Denmark; ○ Professorship for Analytical Chemistry, 9184 Technical University of Munich, Lichtenbergstr. 4, 85748 Garching, Germany; ◆ Chair of Crop Plant Genetics, Faculty of Life Sciences: Food, Nutrition and Health, 26523 University of Bayreuth, Fritz-Hornschuch-Straße 13, 95326 Kulmbach, Germany; ¶ Chair of Plant Breeding, Martin-Luther-University Halle-Wittenberg, Betty-Heimann-Str. 3, 06120 Halle (Saale), Germany; & Leibniz Institute for Food Systems Biology at the Technical University of Munich, Lise-Meitner-Str. 34, 85354 Freising, Germany

**Keywords:** barley, *Bipolaris sorokiniana*, lipids, metabolomics, untargeted LC−MS, MS imaging

## Abstract

Spot blotch of barley (*Hordeum vulgare* L.), caused by *Bipolaris sorokiniana*, is responsible for major losses in crop yield. Breeding-resistant barley varieties have proven to be an effective countermeasure for protecting agricultural production. Plants react to pathogen attacks by up-regulating secondary metabolites. Marker compounds for a *B. sorokiniana* infection are examined by untargeted UPLC-TOF-MS metabolomics and lipidomics techniques. Through the analysis of nine quantitatively resistant and susceptible barley genotypes, derived from the nested association mapping population HEB-25, followed by structure identification experiments and spore germination assays, 57 metabolites are identified. In addition to previously known metabolites, the unknown compounds 5-carboxydidehydroblumenol C-9-*O*-ß-d-glucoside (**46**) and grasshopper ketone 3-sulfate (**47**) were elucidated. 5-Carboxyblumenol C-9-*O*-ß-d-glucoside (**45**) was described for the first time in barley leaves. Pheophytin derivatives, oxylipins, linolenate-conjugated lipids, and flavone glycosides were described for the first time in connection with infections by phytopathogenic fungi or resistance in barley.

## Graphical abstract

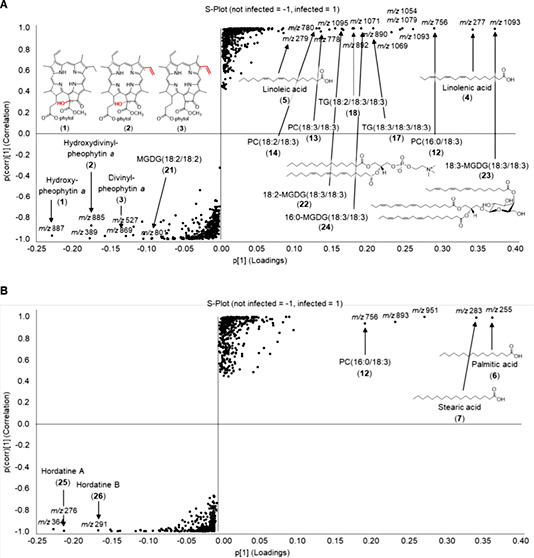

## Introduction

Plants respond to environmental stresses through the accumulation of secondary metabolites. Abiotic stresses, such as drought, heat, salinity stress, or heavy metals, and biotic stresses, like insects, bacteria, fungi, or viruses, activate defense mechanisms in plants. Cultivated barley (*Hordeum vulgare* L. ssp. *vulgare*) is one of the world’s oldest and the fourth most important cereal crop in the world, following wheat, maize, and rice.^1^ The harvested grain is used mainly as animal feed, but barley is also used in the food and beverages industry, especially in the production of beer and whisky.^2^

*Bipolaris sorokiniana* (Sacc.) Shoemaker, is the causal agent of spot blotch, one of the most common foliar diseases of barley worldwide. High temperatures and humidity favor the outbreak of the disease, which can cause significant yield losses.^3,4^

In order to avoid crop losses, preventive measures such as the use of healthy seeds, seed cleaning, crop rotation, fungicide application, and breeding for resistance have been applied.^5^ Fungicide application and breeding for resistance through genetically modified plants are not acceptable to many consumers. Therefore, resistant barley varieties have been generated by means of conventional breeding.^6,7^ Resistance in barley against *B. sorokiniana* has been reported on molecular biological and genetic levels. Barley cultivars partially resistant to *B. sorokiniana* infection have been identified, and several genes and quantitative trait loci (QTLs) causing resistance have been located on different chromosomes.^7–14^ Many studies have focused on the leaf transcriptome and proteome^15–17^ by investigating the molecular responses of barley varieties resistant to *B. sorokiniana* infection, but research on the plant metabolome is limited. Single compound classes were found to accumulate in *B. sorokiniana*-infected barley leaves, which possess antifungal activity, using HPLC-UV analysis.^18,19^ Metabolomics studies picturing a wide structural diversity of metabolites involved in the defense reactions of barley against *B. sorokiniana* are rare.

We hypothesize that biotic stress alters the metabolic profile in barley leaves and that resistant barley lines respond to fungal infection with an upregulated biosynthesis of metabolic defense compounds. To examine this hypothesis, the susceptible barley cultivar Golden Promise and selected lines of the nested association mapping (NAM) population HEB-25 were studied. HEB-25 was developed by crossing 25 diverse wild barley accessions with the German elite spring barley cultivar Barke to capture a representative part of the genetic diversity present in the barley gene pool.^20,21^ Our study compares the metabolomes of (1) infected barley leaves with noninfected controls to find marker metabolites for fungal infection and (2) different resistant or susceptible barley genotypes to find resistance-related metabolites. This work presents a simultaneous analysis of secondary metabolites and lipids in barley leaves infected with *B. sorokiniana*, with marker metabolite identification based upon spectral similarity with reference substances, UPLC-TOF-MS, and NMR spectroscopy.

## Methods

### Chemicals

The reference substances α-linolenic acid (≥99%), linoleic acid (≥99%), palmitic acid (≥99%), stearic acid (≥98.5%), l-glutathione oxidized (≥98%), L-tryptophan (≥98%), tryptamine hydrochloride (99%), DL-malic acid (≥99%), citric acid monohydrate (≥99%), glyceryl trilinolenate (≥97%), 1-palmitoyl-2-linolenoyl-*sn*-glycero-3-phosphatidylcholine (≥97%), isoschaftoside (≥90%), *N,N*′-dicyclohexylcarbodiimide (for synthesis), *N*-hydroxysuccinimide (98%), and agmatine sulfate (≥97%) were obtained from Sigma-Aldrich (Steinheim, Germany). (*E*)-4-hydroxycinnamic acid (98%) from Alfa Aesar (Karlsruhe, Germany) was used. Saponarin (≥98%), schaftoside (≥90%), and isovitexin (≥99%) were purchased from Extrasynthese (Genay, France). Meloside A (>98%) and indole-3-methanamine (>95%) were obtained from MedChemExpress (Sollentuna, Sweden), and trisodium isocitric acid (95%) from Toronto Research Chemicals (Toronto, Canada). 9­(*S*)-Hydroxy-10­(*E*),12­(*Z*),15­(*Z*)-octadecatrienoic acid (>98%), 13-hydroxy-9­(*Z*),11­(*E*)-octadecadienoic acid (≥98%), 9-oxo-10­(*E*),12­(*Z*),15­(*Z*)-octadecatrienoic acid (>98%), 9­(*S*),12­(*S*),13­(*S*)-trihydroxy-10­(*E*),15­(*Z*)-octadecadienoic acid (>98%), 1,2-dilinolenoyl-*sn*-glycero-3-phosphatidylcholine (>98%), 1,2-dilinoleoyl-*sn*-glycero-3-phosphatidylcholine (>98%), 1,2-dilinoleate-3-linolenate-glycerol (>98%), and 1-linoleoyl-2-hydroxy-*sn*-glycero-3-phosphatidylcholine (>99%) were purchased from Larodan (Solna, Sweden). A mixture of mono- and digalactosyldiacylglycerides differing in fatty acids were purchased from Avanti Polar Lipids (Alabaster, Alabama, USA). Grashopper ketone (95%) from Naturewill biotechnology (Chengdu, China) was used.

The cyanoglucosides epiheteroendrin (**38**), sutherlandin (**39**), osmaronin (**40**), dihydroosmaronin (**41**), and epidermin (**42**) were synthesized as previously described.^22^ The acetonitrile, methanol, 2-propanol (Fisher Scientific, Schwerte, Germany), formic acid, and acetic acid (≥99%, VWR, Darmstadt, Germany) used were of LC–MS grade. The water used for LC–MS was purified using an AQUA-Lab-B30-Integrity system (Ransbach-Baubach, Germany). Deuterated solvents D_2_O, DMSO-*d*_6_, and methanol-*d*_4_ were obtained from Sigma-Aldrich (Steinheim, Germany).

### Plant Material and Infection

For the analysis of stress marker compounds, barley *cv*. Golden Promise were grown in the greenhouse as described previously.^23^ Barley seeds were sterilized in 1.2% sodium hypochlorite (3 min, 25 rpm), rinsed 3 times with water (10 min, 25 rpm), and sown (Einheitserde classic CL-T, Bayerische Gärtnereigenossenschaft). Plants were grown in a greenhouse with additional lights HQI-TS 400W/D (Osram) using a day-night cycle of 12 h (24 °C during day, 20 °C during night). A field isolate of *B. sorokiniana*, donated by Corina Vlot-Schuster, was grown on oat plates (10 g rolled oats (Alnatura), Germany), 7.5 g agar–agar (Roth, Karlsruhe, Germany), 500 mL H_2_O) for 1 week at room temperature in the dark and transferred to light for at least 2 weeks. 2 mL of infection solution (0.85 g KH_2_PO_4_ (Merck, Darmstadt, Germany), 0.1 g glucose (Roth, Karlsruhe, Germany), 1 μL Tween 20 in 100 mL H_2_O, pH 6.0) were pipetted onto the plates and spores were scratched off using an inoculation loop. The spore suspension was pipetted into a 5 mL tube and vortexed. After determining the spore concentration under a binocular, the spore suspension was diluted to 100 spores/mL. Barley leaves were spray-inoculated with *B. sorokiniana* until runoff. As controls, uninfected plants were grown under the same conditions and sprayed with demineralized water. The leaves were harvested after the symptoms of spot blotch appeared on the leaf surface and frozen in liquid N_2_ directly after harvesting.

For the analysis of resistance marker compounds, 29 genotypes of the barley nested association mapping (NAM) population HEB-25 ^20^ were selected that genetically differ at a QTL candidate locus for *Drechslera teres* resistance. Respective Barke and HID parents were tested. Plants were cultivated under controlled conditions in the greenhouse (18–20 °C heating temperature, 19–21 °C ventilation temperature, humidity 60–80%, 16 h/day daylight exposure). Eight pots of each genotype were planted, of which four biological replicates were infected with *B. sorokiniana* by spray inoculation (10,000 spores/mL until runoff). Infection occurred at the latest in the BBCH 30 developmental stage. After inoculation, the plants were incubated for 3 days in a climate cabin (18 °C, 80% humidity, darkness) and sprayed several times with demineralized water to keep the leaf wet and promote spore germination. For differentiation, the plants were brought back to the greenhouse (16–18 °C heating temperature, 17–19 °C ventilation temperature, 60–80% humidity, daily accumulation irrigation). Ten, 14, and 17 days after inoculation, the symptoms on the leaves were visually characterized on a scale of 1–9 (Figure S1). A rating of 1 corresponds to a fully healthy plant with no disease symptoms, and a rating of 9 corresponds to the worst possible infestation before the death of the plant. At all time points, leaf samples were taken and immediately frozen.

### Sample Preparation, UPLC-TOF-MS Measurement, and Data Evaluation

Sample preparation, UPLC-TOF-MS analysis, and data evaluation have been described previously.^24^ Details are described in the Supporting Information.

### Isolation of Hordatine Glucosides^28–30^ from Barley Grains

Commercial barley grains (Davert, Ascheberg, Germany) were ground and extracted with 2-propanol/water 80/20 (v/v) for 10 min in an ultrasonic bath. The extract was decanted, and the residue was extracted again two times with 2-propanol/water 80/20 (v/v). The combined supernatants were filtered and concentrated on a rotary evaporator at 30 °C under reduced pressure. The extract was fractionated via medium-pressure liquid chromatography (MPLC) using a Sepacore system (Büchi, Flawil, Switzerland) consisting of two C-605 pumps, a C-620 control unit, a C-660 fraction collector, and a C-635 UV detector. The stationary phase was a PP cartridge (40 × 150 mm) filled with 25–40 μm LiChroprep RP18 material (Merck KGaA, Darmstadt, Germany). The mobile phase consisted of 0.1% formic acid in water (eluent A) and methanol (eluent B). Separation was achieved using a flow rate of 40 mL/min and the following gradient: hold 5% B for 3 min, from 5% B to 28% B in 10 min, hold 28% B for 5 min, from 28% B to 40% B in 5 min, from 40% B to 100% B in 3 min, hold 100% B for 4 min. The effluent was monitored at 280 nm; data were recorded using Sepacore Control Chromatography software (version 1.0, Büchi, Flawil, Switzerland).

Seven MPLC fractions (M1 to M7) were collected and freeze-dried. Fraction M6 was further subfractionated via semipreparative high-performance liquid chromatography (HPLC) on a Jasco HPLC system (Groß-Umstadt, Germany) consisting of two PU-2087 Plus pumps, a DG-2080-53 degaser, and a MD-2010 Plus diode array detector monitoring the effluent at 280 nm using Chrompass 1.8.6.1 (Jasco, Groß-Umstadt, Germany) as software. A 7725i type Rheodyne injection valve (Bensheim, Germany) and a Luna PFP(2) 100 Å column (250 × 10 mm, 5 μm, Phenomenex, Aschaffenburg, Germany) were used. The mobile phase consisted of 30 mmol/L phosphate buffer (pH 2.4, eluent A) and methanol (eluent B). Separation was achieved using a flow rate of 4.4 mL/min and the following gradient: hold 23% B for 4 min, from 23 to 40% B in 30 min, from 40 to 23% B in 1 min.

Seven HPLC fractions (M6H1 to M6H7) were collected and freeze-dried. The fractions M6H5, M6H6, and M6H7 containing hordatine glucosides B (**28**), A (**29**), and C (**30**), respectively, were dissolved in alkalized water (pH 11). Solid phase extraction (SPE) was carried out for phosphate removal using Chromabond C_18_ec columns (45 μm, 70 mL/10,000 mg, Macherey-Nagel, Düren, Germany), which were conditioned with 30 mL methanol and 30 mL alkalized water (pH 11). HPLC fractions were applied, phosphate buffer was eluted with 30 mL alkalized water (pH 11), and hordatine glucosides (**28**–**30**) were eluted with 30 mL acidified methanol (pH 2.8). Hordatine aglycones (**25**–**27**) were obtained by acid hydrolysis of hordatine glucosides. Approximately 1 mg was dissolved in 1 mL of 6 M HCl and left in the dark at room temperature for 24 h. After neutralization with 5 M NaOH, methanol was added to improve solubility.

### Isolation of Marker Compounds from Barley Leaves

500 g leave tissue was ground with a knife mill (Grindomix GM 200, Retsch, Haan, Germany) under liquid nitrogen. 10 × 50 g frozen leaves were extracted with 100 mL methanol each in an ultrasonic bath for 15 min. The extract was filtrated, the residue was extracted again two times with 50 mL methanol each, combined, and concentrated to a volume of 250 mL using a rotary evaporator at 40 °C under reduced pressure. The extract was separated using the same MPLC system as described above. The stationary phase was a Chromabond Flash RS 120 C18ec cartridge (Macherey-Nagel, Düren, Germany) with 0.1% formic acid in water (eluent A) and methanol (eluent B) as the mobile phase. Separation was achieved using a flow rate of 40 mL/min and the following linear gradient: hold 5% B for 3 min, from 5% B to 100% B in 20 min, hold 100% B for 4 min. The effluent was monitored at 280 nm. Eight MPLC fractions (M1 to M8) were collected and freeze-dried.

### Isolation of *p*-CHA (**32**) and *p*-CHDA (**33**)

Fraction M4 (239.8 mg) was dissolved in methanol/water 50/50 (v/v) and separated on the same HPLC system as described above. As stationary phase, a preparative Nucleodur 300-5 C_18_ec column (250 × 21 mm, 5 μm, Macherey-Nagel, Düren, Germany) was used. The mobile phase consisted of 0.1% formic acid in water (eluent A) and acetonitrile (eluent B). Separation was achieved using a flow rate of 15 mL/min and the following linear gradient: hold 5% B for 4 min, from 5 to 30% B in 20 min, from 30 to 5% B in 1 min, hold 5% B for 1 min. The effluent was monitored at 280 nm. Ten fractions (M4H1 to M4H10) were collected and freeze-dried. Fraction M4H8 (15.6 mg) was dissolved in water and subfractionated on a semipreparative Luna Phenyl-Hexyl column (250 × 10 mm, 5 μm, Phenomenex, Aschaffenburg, Germany) using 0.1% formic acid in water (eluent A) and acetonitrile (eluent B) as mobile phase. Separation was achieved using a flow rate of 4.7 mL/min and the following linear gradient: hold 8% B for 4 min, from 8 to 10% B in 1 min, hold 10% B for 10 min, from 10 to 20% B in 9.5 min, from 20% B to 8% B in 0.5 min, hold 8% B for 1 min. The effluent was monitored at 280 nm. Eight fractions (M4H8-1 to M4H8-8) were collected and freeze-dried. *p*-CHA (**32**) and *p*-CHDA (**33**) were isolated from fractions M4H8_–_1 and M4H8-2, respectively. Fraction M4H8-2 (2.1 mg) was dissolved in methanol-*d*_4_ and directly used for NMR spectroscopy. Fraction M4H8-1 (2.4 mg) was further purified by semipreparative HPLC. It was dissolved in 1 mL water and separated on a Kinetex C_18_ column (150 × 10 mm, 5 μm, Phenomenex, Aschaffenburg, Germany) using 0.1% formic acid in water (eluent A) and acetonitrile (eluent B) as mobile phase. Separation was achieved using a flow rate of 4.7 mL/min and the following gradient: hold 5% B for 4 min, from 5 to 10% B in 10 min, from 10 to 5% B in 1 min, hold 5% B for 1 min. The effluent was monitored at 280 nm. The most intense signal was collected, freeze-dried, dissolved in methanol-*d*_4_ and used for NMR spectroscopy.

### Isolation of Apocarotenoids (**45**–**48**)

Fraction M5 (765.7 mg) was dissolved in methanol/water 70/30 (v/v) and separated on a preparative Nucleodur 300-5 C_18_ec column (250 × 21 mm, 5 μm, Macherey-Nagel, Düren, Germany). The mobile phase consisted of 0.1% formic acid in water (eluent A) and acetonitrile (eluent B). Separation was achieved using a flow rate of 20 mL/min and the following gradient: hold 15% B for 4 min, from 15 to 28% B in 20 min, from 28 to 15% B in 1 min. A total of 14 fractions (M5H1 to M5H14) were collected and freeze-dried. Fraction M5H8 (23.2 mg) was dissolved in methanol/water 70/30 (v/v) and separated on a semipreparative Kinetex C_18_ column (150 × 10 mm, 5 μm, Phenomenex, Aschaffenburg, Germany) with 0.1% formic acid in water (eluent A) and acetonitrile (eluent B) as mobile phase, a flow rate of 4.7 mL/min, and the following gradient: hold 10% B for 3 min, from 10 to 15% B in 27 min, from 15 to 30% B in 1 min, hold 30% B for 2 min, from 30 to 10% B in 1 min, hold 10% B for 3 min. The effluent was monitored at 280 nm. A total of 12 fractions (M5H8-0 to M5H8-11) were collected and freeze-dried. Fraction M5H8-9 (8.49 mg) containing 5-carboxyblumenol C glucoside (**45**) was dissolved in DMSO-*d*_6_ and used for NMR spectroscopy. Fraction M5H9 (23.4 mg) was dissolved in methanol/water 70/30 (v/v) and separated on a semipreparative Kinetex C_18_ column (150 × 10 mm, 5 μm, Phenomenex, Aschaffenburg, Germany) with 0.1% formic acid in water (eluent A) and acetonitrile (eluent B) as mobile phase, a flow rate of 4.7 mL/min and the following gradient: hold 10% B for 4 min, from 10 to 15% B in 1 min, hold 15% B for 5 min, from 15 to 17% B in 18 min, from 17 to 100% B in 1 min, hold 100% B for 3 min, from 100 to 10% B in 1 min. The effluent was monitored at 220 nm. Twelve fractions (M5H9-1 to M5H9-12) were collected and freeze-dried. Fraction M5H9-5 containing 5-carboxydidehydroblumenol C glucoside (**46**) was dissolved in DMSO-*d*_6_ and used for NMR spectroscopy. Fraction M5H4 (28.4 mg) was separated on a semipreparative Nucleodur C_18_ Pyramid column (250 × 10 mm, 5 μm, Macherey-Nagel, Düren, Germany) with 0.1% formic acid in water (eluent A) and acetonitrile (eluent B) as mobile phase, a flow rate of 4.7 mL/min and the following gradient: hold 15% B for 4 min, from 15 to 30% B in 20 min, from 30 to 15% B in 1 min. The effluent was monitored at 230 nm. Three fractions (M5H4-1 to M5H4-3) were collected and freeze-dried. M5H4-3 (3.8 mg) and M5H4-2 (2.6 mg) were dissolved in DMSO-*d*_6_ and used for NMR spectroscopy.

### Synthesis of *p*-CA (**31**)

*N*-Hydroxysuccinimide ester of *p*-coumaric acid was synthesized according to Stökigt and Zenk.^25^ *p*-Coumaric acid (79.6 mg, 0.48 mmol) was dissolved in ethyl acetate (20 mL). *N*-hydroxysuccinimide (55.2 mg, 0.48 mmol) and *N*,*N*′-dicyclohexylcarbodiimide (111 mg, 0.54 mmol) were added, and stirred for 24 h at room temperature. The precipitated dicyclohexylurea was filtered off, the filtrate was extracted with 1 M sodium bicarbonate, and the solvent was evaporated.

*N*-Hydroxysuccinimide esters were converted to *p*-CA (**31**) according to Negrel and Smith.^26^ Sodium bicarbonate (13.4 mg, 0.16 mmol) was added to an aqueous solution (20 mL) of agmatine sulfate (36.3 mg, 0.16 mmol). *p*-Coumaroyl-*N*-hydroxy-succinimide ester (41.5 mg, 0.16 mmol) dissolved in acetone (20 mL) was added. The mixture was stirred for 24 h and acidified with 0.5 mL of acetic acid (100%). After removal of acetone by evaporation, the aqueous solution was extracted with ethyl acetate (3 × 20 mL), evaporated, and subjected to preparative HPLC using a 250 × 21 mm Nucleodur 300-5 C_18_ec column (Macherey-Nagel, Düren, Germany), 0.1% formic acid in water (solvent A) and acetonitrile (solvent B) as solvents with a flow rate of 15 mL/min. The effluent was monitored at 280 nm. Separation of *p*-CA (**31**) was achieved using the following linear gradient: hold 20% B for 4 min, from 20 to 40% B in 10 min, from 40 to 100% B in 1 min, hold 100% B for 4 min, from 100 to 20% B in 1 min. The signal eluting at 33% B was collected, the solvent evaporated, and the product characterized by UPLC-TOF-MS and NMR.

### NMR Spectroscopy

The isolated structures were elucidated by LC-TOF-MS, ^1^H NMR, ^13^C NMR, and 2D-NMR experiments (COSY, HSCQ, HMBC) on a 500 or 600 MHz ultrashield plus Avance III spectrometer, each equipped with a Triple Resonance Cryo Probe TCI probehead (Bruker, Rheinstetten, Germany). Chemical shifts were quoted in parts per million (ppm) relative to the solvent signal. The pulse sequences for recording 2D-NMR experiments (COSY, HSQC, and HMBC) were taken from the Bruker software library. Data were processed using Topspin (version 3.1, Bruker, Rheinstetten, Germany) and MestReNova (version 14.2.3-29241, Mestrelab Research, Santiago de Compostela, Spain).

### Desorption Electrospray Ionization Mass Spectrometry Imaging (DESI-MSI)

Thawed leaves were air-dried and mounted onto a Superfrost glass slide using double-sided sticky tape. Desorption electrospray ionization mass spectrometry imaging (DESI-MSI) was performed on a Q-Exactive Plus mass spectrometer equipped with a custom-built 2D automated DESI stage and sprayer assembly. The geometrical parameters were as follows: a sample-to-sprayer distance of 1.5 mm, a sample-to-MS inlet distance of 6 mm, and an inlet-to-sample distance of 0.1 mm. The spray angle was 75°, and the collection angle was 10°. The spray solvent was methanol/water 95/5 (v/v), delivered using a Harvard Apparatus 11 Elite syringe pump at 1.5 μL/min (1 mL syringe volume). The spray voltage was ±4.5 kV, and the nebulization gas was nitrogen (N_2_ 5.0, 7 bar). Imaging was performed in negative and positive ion modes at 75 μm spatial resolution. The instrument settings were 320 °C capillary temperature, a resolution of 70,000 at *m*/*z* 200, an AGC target of 6E5, and an S-Lens setting of 75. In positive ion mode, data was acquired from *m*/*z* 150–800 with a 150 ms injection time, while in negative ion mode, the mass range was *m*/*z* 100–600 with a 250 ms injection time. Individual line scans were converted to.mzML using MSConvert (Proteowizard toolkit v3.0.4043;^27^ and compiled into a single imzML using imzML converter 1.3.^28^ Data visualization and annotation were performed using MSiReader 1.3 ^29^ and Metaspace^30^ as well as comparison with LC-MS data from the same samples.

### Antifungal Bioassay against *B. sorokiniana*

Antifungal activity of preselected marker compounds was tested in a microbroth dilution assay according to Troskie et al.^31^ The activity of the marker compounds (**4**, **5**, **16**, **25**–**30**, **35**, **49**, **51**–**56**) and structural analogues was determined against *B. sorokiniana* using microbroth dilution assays in sterile 96-well microtiter plates.^31^ The broth suspension consisted of fungal spores suspended in 14 PDB (potato dextrose broth). 100 μL spore suspension were added to each well containing 2000 spores per well. Stock solutions (1100 mmol/L) for each antifungal substance were prepared. Coumaric acid, ferulic acid, sinapic acid, tryptophan (**52**), malic acid (**51**), and citric acid (**49**) were dissolved in water, linoleic acid (**5**), linolenic acid (**4**), oleic acid, palmitoleic acid, tryptamine (**53**), indole-3-methanamine (**54**), and hordatine (glucoside) mixture (**25**–**30**) in ethanol/water 70/30 (v/v) and saponarin (**56**), schaftoside (**35**), isovitexin (**55**), isoorientin, and apigenin in DMSO/water 5/95 (v/v). Ten μL of each antifungal stock solution were added to each well resulting in a final concentration of 10 mmol/L of each substance. Negative controls received 10 μL of pure solvent (water, DMSO/water 5/95 or ethanol/water 70/30). Positive control was 10 μL of hexanoic acid in ethanol/water 70/30 (v/v) with a final concentration of 10 mmol/L in the wells. Microtiter plates were covered and incubated on a shaking plate (5 rpm) at 18–20 °C for 72 h. Light dispersion of each well was spectrophotometrically determined at 595 nm using a Tecan Infinite M200 (Männedorf, Switzerland) microtiter plate reader. The data from the quadruplicate determination were evaluated using *t*-test.

## Results

Marker compounds for biotic stress responses in barley infected with *B. sorokiniana* were evaluated using the barley cultivar Golden Promise. The leaves of infected plants and noninfected control plants were analyzed using a combined lipid and metabolomics approach. Metabolites were analyzed in samples from both entire leaves and isolated leaf areas displaying chlorotic symptoms, and compared to those in uninfected controls.

The measurements of secondary metabolites and lipids in both positive and negative ionization modes yielded a total of 8,000 detected mass-to-charge ratio retention time pairs (*m*/*z*-*t*_R_ pair). PCA and OPLS-DA were used for data reduction and finding compounds of interest. Each *m*/*z*-*t*_R_ pair is shown as a dot in the *S*-plot (Figures 1 and 2). The *x*-axis of the *S*-plot describes the influence of a compound based on the difference between treated and control samples, whereas the *y*-axis represents the statistical significance. Substances occurring at one of the edges of the *S*-plot were present in a higher amount in the respective group.^32^ The upregulation of a metabolite in the control plants corresponds to a downregulation in the infected leaves. Of all MS features, sixty-nine MS features were statistically significantly different in the compared samples (ANOVA *p* ≤ 0.05, fold change ≥2). The most relevant marker compounds were identified by cochromatography using commercially available reference standards, isolation, synthesis, or MS^2^ experiments (Table 1). As a control, fungal spores of *B. sorokiniana* were extracted and analyzed in the same way to identify metabolites of fungal origin.

**Figure 1:**
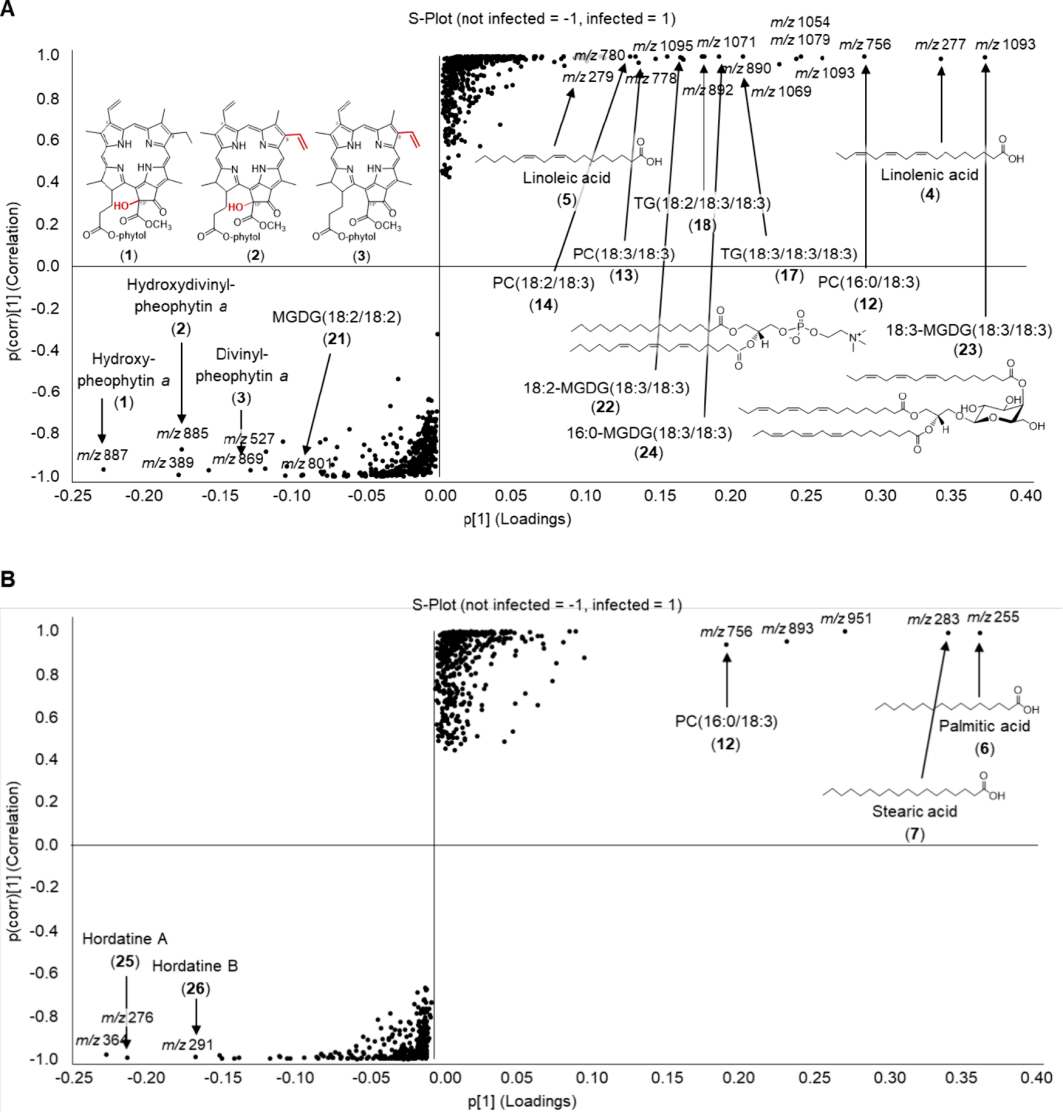
*S*-plots of the lipidomics analysis of (A) whole barley leaves infected with *B. sorokiniana* and (B) excised symptomatic spots. Each ● describes a certain *m*/*z* at a certain retention time. Features were filtered by an ANOVA *p*-value ≤ 0.05 and a fold-change ≥ 2. The *x*-axis describes the degree of the contribution of a metabolite to the group difference, whereas the *y*-axis represents the significance between the groups.

**Table 1:** Up- (**▲**) and Downregulated (**▼**) Compounds Detected in Barley Leaves *cv* ^a^

no	substance name	*m*/*z*	adduct	*m*/*z* fragments	*t*_R_ (min)	method	up-/down-regulated	ID level
Lipids and Fatty Acids								
**1**	hydroxypheophytin *a*	887.5645	[M+H]^+^	869.5607	9.46	li	**▼**	2
		909.5463	[M+Na]^+^	609.2706				
		925.5203	[M+K]^+^	591.2601				
				577.2426				
				549.2481				
				531.2422				
**2**	hydroxydivinyl-pheophytin *a*	885.5504	[M+H]^+^	607.2549	9.19	li	**▼**	2
				589.2426				
				575.2272				
				547.2372				
**3**	divinylpheophytin *a*	869.5592	[M+H]^+^	591.2607	9.33	li	**▼**	2
		891.5395	[M+Na]^+^	559.2345				
				531.2396				
**4**	linolenic acid	277.2171	[M–H]^−^		3.51	li	**▲**	1
**5**	linoleic acid	279.2321	[M-H]^−^		4.1	li	**▲**	1
**6**	palmitic acid	255.2324	[M-H]^−^		4.41	li	**▲**	1
**7**	stearic acid	283.2636	[M-H]^−^		5.42	li	**▲**	1
**8**	13-HODE	295.2262	[M-H]^−^	195.1366	9.65	aq	**▲**	1
		277.2153	[M–H-H_2_O]^−^	113.0954				
**9**	9-HOTrE	293.2108	[M-H]^−^	171.1025	9.47	aq	**▲**	1
		275.2002	[M–H-H_2_O]^−^	121.1007				
		277.2166	[M+H–H_2_O]^+^					
**10**	9-OxoOTrE	291.1974	[M–H]^−^	185.1193	9.55	aq	**▲**	1
				125.0971				
				121.0983				
**11**	9,12,13-TriHODE	327.2175	[M–H]^−^	211.1335	8.43	aq	**▲**	1
				171.1022				
				152.9958				
**12**	PC(16:0/18:3)	756.5554	[M+H]^+^	184.0739	7.37	li	**▲**	1
		778.5357	[M+Na]^+^	86.0972				
		794.5106	[M+K]^+^					
**13**	PC(18:3/18:3)	778.5397	[M+H]^+^	741.4473	6.41	li	**▲**	1
		800.5209	[M+Na]^+^	617.4545				
				595.4731				
				184.0738				
				146.9817				
				104.1070				
				86.0965				
**14**	PC(18:2/18:3)	780.5544	[M+H]^+^	743.4603	6.95	li	**▲**	2
		802.5357	[M+Na]^+^	619.4711				
				597.4886				
				184.0736				
				146.9833				
				104.1084				
				86.0965				
				184.0736				
				104.1070				
				86.0965				
**15**	LysoPC(18:3)	518.3250	[M+H]^+^	335.2581	9.36	aq	**▲**	2
		540.3060	[M+Na]^+^	184.0734				
				86.0965				
		562.3148	[M+FA-H]^−^	277.2163				
				242.0818				
				152.9949				
				78.9588				
**16**	LysoPC(18:2)	520.3469	[M+H]^+^	337.2752	9.59	aq	**▲**	1
		542.3255	[M+Na]^+^	184.0747				
				86.0965				
		564.3305	[M+FA-H]^−^	504.3110				
				279.2332				
				242.0794				
				224.0690				
				152.9953				
				78.9570				
**17**	TG(18:3/18:3/18:3)	890.7242	[M+NH_4_]^+^	595.4727	10.01	li	**▲**	1
		895.6788	[M+Na]^+^	335.2585				
		873.6967	[M+H]^+^	261.2216				
		871.6821	[M–H]^−^	773.5215				
				593.3553				
				277.2167				
**18**	TG(18:2/18:3/18:3)	892.7391	[M+NH_4_]^+^	892.7443	10.14	li	**▲**	1
		897.6942	[M+Na]^+^	875.7128				
		875.7124	[M+H]^+^	857.6993				
				597.4882				
				595.4708				
				337.2720				
				335.2563				
				319.2626				
				317.2471				
				263.2371				
				261.2200				
				245.2257				
				243.2109				
**19**	DGDG(18:3/18:3)	959.5713	[M+Na]^+^	797.6251	11.06	aq	**▼**	1
				681.3460				
				613.4836				
				595.4742				
				335.2577				
				261.2216				
**20**	MGDG(18:3/18:3)	797.5183	[M+Na]^+^	792.5621	11.48	aq	**▼**	1
		813.4914	[M+K]^+^	613.4841				
				595.4724				
				519.2931				
				335.2587				
				261.2212				
				241.1938				
**21**	MGDG(18:2/18:2)	801.5493	[M+Na]^+^	617.5135	8.06	li	**▼**	2
		817.5231	[M+K]^+^	599.5034				
		796.5934	[M+NH_4_]^+^	521.3085				
				337.2738				
				263.2386				
**22**	18:2-MGDG(18:3/18:3)	1095.7723	[M+HAc-H]^−^	775.5350	9.76	li	**▲**	2
		1035.7540	[M-H]^−^	773.5154				
				515.3105				
				513.3105				
				279.2306				
				277.2144				
**23**	18:3-MGDG(18:3/18:3)	1093.7572	[M+HAc-H]^−^	773.5215	9.62	li	**▲**	2
		1033.7362	[M-H]^−^	513.3055				
				277.2180				
**24**	16:0-MGDG(18:3/18:3)	1071.7726	[M+HAc-H]^−^	773.5215	9.88	li	**▲**	2
		1011.7524	[M-H]^−^	751.5397				
				513.3055				
				491.3209				
				277.2144				
				255.2324				
Phenolamides								
**25a**	(*Z*)*-h*ordatine A	276.1589	[M+2H]^2+^	291.0667	4.75	aq	**▼**	1
		551.3101	[M+H]^+^	265.0876				
				263.0705				
				247.0772				
				237.0916				
				235.0770				
				219.0818				
				178.0780				
				157.1082				
				131.1312				
				114.1034				
				72.0814				
**26a**	(*Z*)*-h*ordatine B	291.1639	[M+2H]^2+^	564.2929	4.66	aq	**▼**	1
		581.3195	[M+H]^+^	539.2977				
				451.1976				
				295.0958				
				262.0827				
				235.0770				
				222.0677				
				157.1084				
				131.1287				
				129.1023				
				114.1034				
**27a**	(*Z*)*-h*ordatine C	611.3308	[M+2H]^2+^	594.3038	4.81	aq	**▼**	1
		306.1689	[M+H]^+^	569.3087				
				481.2095				
				351.0852				
				325.1071				
				293.0801				
				265.0493				
				131.0861				
**25b**	(*E*)*-h*ordatine A	276.1591	[M+2H]^2+^	s. (*Z*)*-*isomer	4.95	aq	**▼**	2
		551.3102	[M + H]^+^					
**26b**	(*E*)*-h*ordatine B	291.1642	[M+2H]^2+^	s. (*Z*)*-*isomer	4.92	aq	**▼**	2
		581.3195	[M + H]^+^					
**27b**	(*E*)*-h*ordatine C	611.3308	[M+2H]^2+^	s. (*Z*)*-*isomer	5.08	aq	**▼**	2
		306.1689	[M + H]^+^					
**28**	(*Z*)-hordatine A glucoside	357.1849	[M+2H]^2+^	157.1087	4.21	aq	**▼**	1
		713.3618	[M + H]^+^	131.1293				
				114.1031				
				72.0808				
**29**	(*Z*)-hordatine B glucoside	372.1902	[M+2H]^2+^	726.3458	4.13	aq	**▼**	1
		743.3724	[M + H]^+^	701.3500				
				295.0969				
				235.0757				
				189.0547				
				131.1291				
**30**	(*Z*)-hordatine C glucoside	773.3824	[M+2H]^2+^	481.2096	4.23	aq	**▼**	1
		387.1946	[M + H]^+^	325.1065				
				131.1292				
				114.1027				
**31**	(*Z*)*-p*-coumaroyl-agmatine	277.1664	[M + H]^+^	147.0442	4.18	aq	**▼**	1
				119.0486				
				114.1034				
				91.0553				
		275.1508	[M-H]^−^	144.0454				
				119.0502				
				117.0346				
**32**	(*Z*)*-p*-coumaroyl-3-hydroxyagmatine	293.1612	[M + H]^+^	147.0442	3.77	aq	**▲**	1
				130.0972				
				129.1145				
				119.0510				
				113.0717				
				91.0553				
				88.0773				
				70.0659				
**33**	(*Z*)*-p*-coumaroyl-3-hydroxydehydro-agmatine	291.1458	[M + H]^+^	213.1005	3.81	aq	**▲**	1
		273.1350	[M+H–H_2_O]^+^	147.0442				
				127.0987				
				119.0510				
				113.0717				
				91.0553				
				85.0762				
				69.0453				
Flavone glucosides								
**34**	isovitexin 7-*O*-rhamnosylglucoside	741.2256	[M+H]^+^	617.1501	5.06	aq	**▼**	2
		763.2056	[M+Na]^+^	455.0973				
				437.0868				
				397.0918				
				379.0823				
				367.0823				
				337.0716				
				313.0716				
				283.0611				
				271.0601				
		739.2093	[M-H]^−^	619.1636				
				473.1065				
				445.1123				
				431.0995				
				341.0546				
				311.0546				
				283.0604				
				269.0435				
**35**	schaftoside (apigenin 6-*C*-glucoside 8-*C*-arabinoside)	563.1413	[M-H]^−^	473.1065	5.1	aq	**▼**	1
				443.0994				
				383.0768				
				353.0664				
**36**	apigenin 7-*O*-arabinosylglucoside	563.1413	[M-H]^−^	443.0984	6.1	aq	**▼**	3
				431.0995				
				413.0885				
				311.0546				
				269.0450				
**37**	isovitexin 2″-*O*-feruloylglucoside	771.2136	[M+H]^+^	433.1129	5.95	aq	**▲**	2
		793.1950	[M+Na]^+^	415.1026				
		753.2032	[M+H–H_2_O]^+^	397.0923				
				379.0816				
				367.0816				
				337.0716				
				313.0716				
				283.0605				
				177.0551				
		769.1977	[M-H]^−^	473.1065				
				445.1123				
				431.0995				
				341.0666				
				325.0716				
				311.0546				
Cyanoglucosides								
**38**	epiheteroendrin	306.1190	[M+FA-H]^−^	188.0557	4.45	aq	**▼**	1
		260.1134	[M-H]^−^	161.0451				
		284.1149	[M+Na]^+^	113.0230				
				101.0235				
				85.0286				
**39**	sutherlandin	276.1080	[M+H]^+^	180.0652	2.26	aq	**▼**	1
		298.0901	[M+Na]^+^	156.0650				
		314.0639	[M+K]^+^	114.0548				
		258.0966	[M+H–H_2_O]^+^	97.0280				
		320.0973	[M+FA-H]^−^					
**40**	osmaronin	260.1129	[M+H]^+^	230.5589	3.52	aq	**▼**	1
		282.0952	[M+Na]^+^	210.9926				
		299.0746	[M+K]^+^	149.5334				
		242.1023	[M+H–H_2_O]^+^	140.0695				
		304.1017	[M+FA-H]^−^	98.0595				
				96.0610				
**41**	dihydroosmaronin	262.1321	[M+H]^+^	142.0862	3.62	aq	**▼**	1
		284.1108	[M+Na]^+^	124.0754				
		300.0845	[M+K]^+^	100.0757				
		244.1187	[M+H–H_2_O]^+^	97.028				
		306.1185	[M+FA-H]^−^	85.0286				
				73.0281				
				69.0338				
				61.0288				
**42**	epidermin	262.1307	[M+H]^+^	231.5678	3.31	aq	**▼**	1
		284.1104	[M+Na]^+^	204.0855				
		300.0822	[M+K]^+^	180.0859				
		279.1634	[M+H–H_2_O]^+^	163.0594				
		244.1212	[M+FA-H]^−^	145.0492				
		306.1187		127.0381				
				98.0601				
				97.0280				
Other metabolites								
**43**	oxyglutathione	611.1456	[M-H]^−^	307.0748	2.62	aq	**▼**	1
				272.0852				
				254.0744				
				242.4688				
				210.0868				
				179.0437				
				160.0037				
				143.0436				
				128.0331				
				99.0557				
				74.0241				
**44**	dihydroxybenzoic acid hexoside	315.0717	[M-H]^−^	153.0175	3.96	aq	**▼**	3
				152.0106				
				109.0276				
				108.0212				
				81.0336				
Apocarotenoids								
**45**	5-carboxyblumenol C 9-*O*-*ß*-glucoside	401.1803	[M-H]^−^	221.1168	5.87	aq	**▲**	1
**46**	5-carboxydidehydro-blumenol C 9-*O*-*ß*-glucoside	399.1657	[M-H]^−^	219.1030	6.05	aq	**▲**	1
				176.1165				
				175.1114				
				160.0894				
				119.0338				
				101.0255				
				89.0229				
				71.0126				
**47**	grasshopper ketone-3-sulfate	303.0907	[M-H]^−^	96.9590	5.04	aq	**▲**	1
**48**	unknown (C_13_H_21_SO_6_)	305.0690	[M-H]^−^	267.03	4.8	aq	**▲**	3
				225.1111				
				118.9414				
				96.9590				

a Golden Promise infected with *B. sorokiniana*. *m*/*z*, mass-to-charge ratio of precursor ion; *m/z* fragments, mass-to-charge ratio of MS^2^ fragment ions; *t*_R_, retention time; li, lipidomics, aq, metabolomics; ID level, Identification level according to Metabolomics Standards Initiative:^171^ (1) identified compound using reference substances, (2) putatively annotated compound based on physicochemical properties and spectral similarity with public spectral libraries, (3) putatively characterized compound class based on characteristic physicochemical properties of a chemical compound class, or by spectral similarity to known compounds of a chemical class; ^f^FA, formic acid; ^g^Hac, acetic acid.

**Figure 2:**
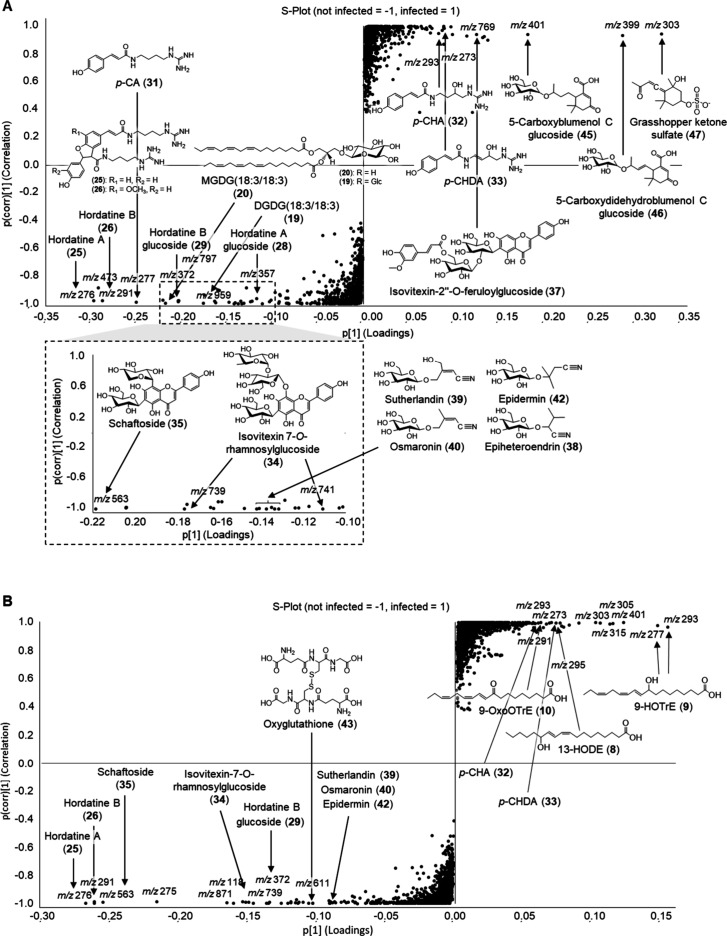
*S*-plots of the metabolomics analysis of (A) whole barley leaves infected with *B. sorokiniana* and (B) excised symptomatic spots. Features were filtered by an ANOVA *p*-value ≤ 0.05 and a fold change ≥2.

### Lipids in *B. sorokiniana*-Infected Barley

Infection of barley *cv.* Golden Promise with *B. sorokiniana* resulted in the metabolic regulation of several lipid compound classes including free fatty acids (**4**–**11**), linolenate-conjugated lipids (**12**–**24**), and pheophytine derivatives (**1**–**3**) (Figure 1).

### Identification of Fatty Acids (**4**–**7**) and Oxylipins (**8**–**11**)

In the infected whole leaves, the unsaturated fatty acids linolenic acid (**4**) and linoleic acid (**5**) were more abundant compared to uninfected controls, whereas the saturated palmitic (**6**) and stearic acids (**7**) were upregulated in chlorotic leaf spots. Moreover, fatty acid oxidation products (**8**–**11**) were identified in the spots. Hydroxy fatty acids (**8**,**9**) occurred in negative ionization mode as [M-H]^−^ and [M-H_2_O–H]^−^ adduct ions. Lipoxygenases 9-LOX and 13-LOX, both present in barley,^33,34^ metabolize C_18_ unsaturated fatty acids, such as linoleic and linolenic acid, into the corresponding 9- or 13-hydroperoxy fatty acids. The hydroxylation at positions 9 and 13 can be distinguished by the characteristic fragmentation between the hydroxy group and neighboring (*E*)-double bond, resulting in either a fragment ion with *m*/*z* 171 specific for the 9-isomer, or *m*/*z* 195 for the 13-isomer.^35,36^ By co-chromatography with reference substances, 13-hydroxy-octadecadienoic acid (13-HODE, **8**), 9-hydroxy-octadecatrienoic acid (9-HOTrE, **9**), and 9-oxo-octadecatrienoic acid (9-OxoOTrE, **10**) were identified in the infected leaf areas. Commercial 13-OxoOTrE had a higher retention time on the C_18_ column compared to 9-OxoOTrE (**10**).

### Identification of Linolenate-Conjugated Lipids (**12**–**24**)

Lipids containing linolenic acid showed characteristic fragments with *m*/*z* 263 in ESI^+^ (C_18_H_31_O) and *m*/*z* 277 in ESI^–^ (C_18_H_31_O_2_) mode. Triglycerides and phosphocholines with linoleic and linolenic acid side chains were identified (**12**–**18**). Phosphocholines (**12**–**16**) indicated characteristic fragmentation in ESI^+^ mode, including neutral losses of 183 and 59 Da, the fragment ions *m*/*z* 184, 104, and 86 representing the phosphocholine headgroup, and *m*/*z* 147 corresponding to the sodiated five-member cyclophosphane.^37^ The observed fragment ions were in agreement with the calculated *m*/*z* values due to the elemental composition or predicted by LIPID-MAPS (mass error <10 ppm, Tables S1 and S2). The annotated structures were verified using reference substances. Although the positional isomers PC­(16:0/18:3) and PC­(18:3/16:0) were not distinguishable, it can be assumed that palmitic acid is bound at position sn1 and linolenic acid on position sn2, representing the naturally occurring structure of phospholipids with saturated sn1 and unsaturated sn2 fatty acids.^38^

Moreover, 18:3-fatty acid residues could be observed in polar lipid components such as monogalactosyldiacylglycerol (MGDG, **20**,**21**) and digalactosyldiacylglycerol (DGDG, **19**). The proposed metabolites were identified using surrogate standards as described previously.^39^

In addition, enzymatically modified MGDG species with esterification at the 6′-hydroxyl group of galactose with another fatty acid were postulated.^40^ The features *m*/*z* 1095, 1093, and 1071 were assigned as the acetate adducts of the acylated MGDG species 18:2-MGDG­(18:3/18:3) (**22**), 18:3-MGDG­(18:3/18:3) (**23**), and 16:0-MGDG­(18:3/18:3) (**24**), respectively. The UPLC-TOF-MS^2^ data were in agreement with the accurate masses calculated due to the elemental composition (mass error <5 ppm, Table S3). The galactose-conjugated fatty acid was determined due to characteristic neutral losses of the acylated galactose headgroup., and 18:2-MGDG­(18:3/18:3) (**22**) highlights a neutral loss of of 441 Da, 18:3-MGDG­(18:3/18:3) (**23**) of 439 Da, and 16:0-MGDG­(18:3/18:3) (**24**) of 417 Da, resulting in the ESI^+^ fragments *m*/*z* 613 and 423, 401, and 425, respectively. Nilsson et al. (2015) determined the fatty acid composition of acyl-MGDG in different plant species, including barley, and observed 18:3, 16:0, and 18:2 in descending order esterified to the headgroup.^41^ This corresponded to the relative peak areas in the analyzed samples.

### Identification of Pheophytine *a* Derivatives (**1**–**3**)

Hydroxypheophytin *a* (**1**), hydroxydivinylpheophytin *a* (**2**), and divinylpheophytin *a* (**3**) were identified based on their specific MS^2^ fragmentation patterns. In general, pheophytins reveal fragment ions with [(M+H)–278]^+^, [(M+H)–278–32]^+^, and [(M+H)–278–60]^+^, indicating the cleavage of the phytyl chain and the loss of a carboxymethyl group from the precursor ion.^42^ Similar fragment ions of hydroxydivinylpheophytin *a* (**2**) and hydroxypheophytin *a* (**3**) with a mass difference of 2 Da as well as the retention time order *m*/*z* 885 > *m*/*z* 869 > *m*/*z* 887, underline the presence of hydroxydivinylpheophytin *a* (**2**) instead of its isomer pheophytin *b* (*m*/*z* 885). The MS^2^ data were in agreement with the accurate masses calculated due to the elemental composition (mass error <5 ppm, Table S4), as well as data reported previously.^42–45^ Whereas the relative peak areas of pheophytin *a* were the same in healthy and infected plants, the metabolites **1**–**3** decreased after infection.

### Secondary Metabolites in *B. sorokiniana* Infected Barley

The metabolomics analysis of infected barley *cv.*3– Golden Promise showed the up- or downregulation of defense-related compounds, such as hordatines (**25**–**30**), phenolamides (**31**–**33**), flavone glucosides (**34**–**37**), cyanoglucosides (**38**–**42**), and apocarotenoids (**45**–**47**) (Figure 2A). In pathogen-induced local lesions, fatty acid oxidation products (**8**–**10**) were additionally upregulated (Figure 2B).

### Identification of Hordatines (**25**–**30**) and Coumaroylagmatines (**31**–**33**)

Hordatines (**25**–**27**) and the corresponding glucosides (**28**–**30**), observed as [M+H]^+^ and [M+2H]^2+^ adducts appearing at the same retention time, showed characteristic neutral fragment losses of 17 Da (ammonia), 42 Da (CH_2_N_2_ moiety of guanidine), and 130 Da (agmatine), which were accompanied by the subsequent losses of CO (28 Da) and CO_2_ (44 Da). Additionally, hordatine glucosides (**28**–**30**) revealed a neutral loss of 162 Da, indicating the cleavage of the hexose moiety. For both hordatine glucosides and aglycones (**25**–**30**) the fragment ions *m*/*z* 157, 131, 114, and 72 (specific for agmatine) and *m*/*z* 235 (phenylindole substructure) were perceived.

Hordatine glucosides (**28**–**30**) were isolated from barley grains and structurally confirmed by UPLC-TOF-MS and NMR spectroscopy (Figure S2, Tables S5 and S6). Two isomeric structures of hordatine glucosides A, B, and C were separated. The earlier eluting (*Z*)-isomers showed a coupling constant of the protons H–C(7) and H–C(8) with *J* ≈ 12 Hz, the (*E*)-isomers of *J* ≈ 16 Hz. The coupling constant of the anomeric proton at C(1″″) with *J* = 7.2 Hz is typical for β-d-glycosides. The position of the glucose moiety was determined by the correlation of the proton H–C(1″″) to C(4′). The position of the agmatine residue was identified based on the protons H-C(7) and H-C(8) correlating to C(3). The additional methoxy group of hordatine B glucoside(29) was located at C(5) due to the correlation of the singlet protons at H_3_-C­(10).

After acidic hydrolysis, the structures of hordatine aglycones (**25**–**27**) were confirmed. The UPLC-TOF-MS^2^ data of hordatines and hordatine glucosides were in agreement with the accurate masses calculated due to the elemental composition (mass error <5 ppm, Table S7). The MS and NMR data were also in agreement with the literature.^46–48^

The MS features *m*/*z* 277, 293, and 273 were annotated as *p*-coumaroylagmatine (*p*-CA, **31**), and its oxidation products *p*-coumaroyl-3-hydroxyagmatine (*p*-CHA, **32**) and *p*-coumaroyl-3-hydroxydehydroagmatine (*p*-CHDA, **33**). All compounds indicated specific fragments at *m*/*z* 147, 119, and 91 originating from the coumaroyl moiety (Figure S3). The accurate masses of precursor and fragment ions were in agreement with those calculated due to the elemental composition (mass error <5 ppm, Table S8), as well as with data reported previously.^49–51^ *p*-CA (**31**) was synthesized through the amidation of (*E*)-*p*-coumaric acid with agmatine. The observed NMR data was in agreement with literature data (Figure S4, Table S9).^52,53^ The signals at 7.4 and 6.8 ppm (*J* = 8.7 Hz) indicated a *para*-substituted benzene. The coupling constant of the protons H-C(7) and H-C(8) with *J* ≈ 16 Hz was indicative of an (*E*)-double bond. The product contained approximately 12% (*Z*)-*p*-CA. The isomers were separated chromatographically with the (*Z*)-isomer eluting earlier. Light exposition increased the first eluting peak and decreased the second peak in the same ratio.

*p*-CHDA (**33**) appeared with the fragment *m*/*z* 273 as the most abundant ion in positive ionization mode. MS^2^ experiments of the [M + H]^+^ precursor ion *m*/*z* 291 and the [M+H–H_2_O]^+^ adduct *m*/*z* 273 revealed the same fragment ions. To verify that *m*/*z* 273 is an in-source fragmentation product of *m*/*z* 291, collision energy was varied. The intensity of *m*/*z* 273 increased with enhancing collision energy, whereas the intensity of *m*/*z* 291 decreased in the same ratio (Figure S5).

To confirm these observations, *p*-CHA (**32**) and *p*-CHDA (**33**) were isolated from barley leaves and structurally characterized using NMR and mass spectroscopy (Figure S4, Table S9). The position of the hydroxy group at C(3′) was determined in the HSQC as well as the COSY spectrum. Because of the asymmetric C(3′), the geminal protons of the neighboring C-atoms showed two diastereotropic signals, referred to as α and β proton. The proton at C(3′) correlated with the two protons at C(2‘) (1.64 and 1.80 ppm) and the two protons on C(4′) (3.29 and 3.43 ppm). In contrast, *p*-CHDA (**33**) highlighted CH groups instead of CH_2_ on positions 1′ and 2′ in the HSQC spectrum and higher chemical shifts compared to the saturated compound, suggesting a double bond between C(1′) and C(2′). The observed NMR data of *p*-CHA (**32**) were in agreement with literature data.^52,54,55^ For *p*-CHDA (**33**) no NMR data has been published so far. Thus, in this study, *p*-CHDA (**33**) was isolated from barley and fully characterized for the first time.

### Identification of Cyanoglucosides (**38**–**42**)

Five cyanoglucosides (**38**–**42**) were identified in the control leaves of barley *cv.* Golden Promise (Figure S6, Table S10). For all substances, the [M+Na]^+^ adduct ion was the most abundant, except for epiheteroendrin (**38**), where the formic acid adduct [M+FA–H]^−^ was more relevant. In positive ESI mode, neutral fragment losses of 162 Da resulting from the cleavage of the hexose unit and 10 Da corresponding to the cross-ring cleavage of the glucose were observed.

### Identification of Flavone Glucosides (**34**–**37**, **55**–**57**)

Several conjugates of isovitexin (apigenin 6-*C*-β-d-glucopyranoside) were annotated as marker compounds (**34**–**37**, **55**–**57**). All compounds revealed specific fragment ions at *m*/*z* 431, 341, 311, and 283 in negative ionization mode (Table S11). These ions were also found in the spectrum of the aglycone isovitexin (**55**). Isomeric saponarin (isovitexin 7-*O*-glucoside, **56**) and meloside A (isovitexin 2″-*O*-glucoside, **57**) were verified using reference standards that showed different specific MS fragmentation patterns in negative ESI mode (Figure S7A). Meloside A (**57**) showed a neutral loss of 180 Da (C_6_H_12_O_6_), which indicated the cleavage of the aliphatic *O*-glucose. In contrast, saponarin (**56**) indicated a neutral loss of 162 Da (C_6_H_10_O_5_), which states that the phenolic *O*-glucose is split off without the C(1′) hydroxyl group. In positive ESI mode, both substances showed different intensities of certain fragment ions (Figure S7B). Saponarin (**56**) indicated a higher abundance of *m*/*z* 283 compared to *m*/*z* 313, whereas meloside A (**57**) showed *m*/*z* 313 as the most intensive fragment ion. The observed MS data is in alignment with the literature.^47,56–58^

The infection with *B. sorokiniana* evoked an upregulation of HCA-conjugated flavone glucosides. Isovitexin 2″-*O*-feruloylglucoside (**34**) was significantly elevated in infected barley *cv.* Golden Promise (Figure 2A) and in the more susceptible barley genotypes (Figure 6). The ESI^+^ fragment ion at *m*/*z* 177 and the neutral loss of 176 Da in ESI^–^ indicated the cleavage of the ferulic acid subunit. The neutral loss of 338 Da from the precursor ion indicated the loss of the feruloylglucose moiety and resulted in the base peak of the aglycone at *m*/*z* 431 in negative ionization mode. In isovitexin 2″-*O*-feruloylglucoside (**34**), the cross-ring cleavage occurs after the cleavage of the feruloylglucose unit, whereas for isovitexin 7-*O*-feruloylglucoside, both cleavages happen simultaneously.^47^ Therefore, the fragments [(M–H)–90]^−^ and [(M–H)–120]^−^ originating from the cross-ring cleavage of the hexose at C5 are characteristic of 7-*O*-glucosides.^57^ The absence of the fragments with [(M-H)-90]^−^ (*m*/*z* 680) and [(M-H)-120]^−^ (*m*/*z* 650) as well as the high abundance of [(M-H)-338–90]^−^ (*m*/*z* 431) and [(M-H)-338–120]^−^ (*m*/*z* 311) in the MS^2^ spectrum indicated the presence of a 2″-*O*-glucoside.

The presence of schaftoside (apigenin 6-*C*-glucoside-8-*C*-arabinoside, **35**) was described in barley leaves.^47^ The absence of the ESI^–^ fragment ion *m*/*z* 431, which is characteristic of flavone *O*-glucosides, indicated a C-linkage between aglycone and sugars. The cross-ring cleavage of di-*C*-glycosides within the sugar at C6 is preferred compared to C8.^59,60^ The position of the C–C-linkage and the distinction of schaftoside (**35**) from isoschaftoside (apigenin 6-*C*-arabinoside-8-*C*-glucoside) were determined due to the particular intensities of the MS^2^ fragments originating from the cross-ring cleavage. 6-C-glucosides show a higher abundance of [(M-H)-120]^−^ at *m*/*z* 443 in contrast to [(M-H)­90]^−^ at *m*/*z* 473 which differentiates them from 8-*C*-glucosides.^61^ The fragment ions at [(M–H)–120–60]^−^ (*m*/*z* 383) and [(M–H)–120–90]^−^ (*m*/*z* 353) indicated the cleavage of the glucose at C6, followed by the fragmentation of the arabinose unit at C8.^62^ Moreover, the fragment at [(M-H)-120–60]^−^ (*m*/*z* 383) as well as the very low intensity of [(M–H)–60]^−^ at *m*/*z* 503 confirmed the presence of an 8-*C*-arabinoside instead of an 8-*C*-glucoside.^61,63,64^ To confirm the presence of schaftoside (**35**) instead of isoschaftoside in the analyzed barley samples, reference standards of both substances were separated chromatographically, with schaftoside (**35**) eluting earlier from the C_18_ column.

### Identification of Apocarotenoids (**45**–**47**)

The marker compounds **45** and **46** showed [M-H]^−^ adducts of *m*/*z* 401 and 399 in negative ESI mode, [M+Na]^+^ adducts of *m*/*z* 425 and 423, and [M+K]^+^ adducts of *m*/*z* 441 and 439 in positive ESI mode. The loss of a hexose unit was observed through the neutral losses of 180 and 162 Da in ESI^–^ and ESI^+^ modes, respectively. Additional neutral losses of 44 and 46 Da indicated the loss of CO_2_.

The exact structures of **45** and **46** were determined by NMR spectroscopy after isolation from barley leaves (Figure S8, Table S12). The protons of the methylene group H-C­(2α) and H-C­(2β) form a spin system, resulting in a doublet with a coupling constant of ^2^ *J* = 17.4 Hz, which is a typical value for geminal coupling in methylene groups with diastereotopic protons.^65^ In addition, the methylene group on C2 did not show any further coupling, indicating that it is surrounded by quaternary carbon atoms. The configuration of the O-glucoside was determined by the coupling constant of the doublet of H-C(1′) with *J* = 7.8 Hz, which indicates a *ß*-D configuration.

The HMBC spectrum reveals the key correlation between the glycosidic and the aliphatic region (Figure S9A). Here, the protons H-C(1′) and H-C(9) couple with C9 or C1′, respectively. Thus, the *O*-glucoside is bound at position C9. In addition, correlation signals of three methyl groups were observed (Figure S9B). Since the two methyl groups, H_3_C­(11) and H_3_C­(12), were attached to the same quaternary carbon atom, they highlighted the same correlations to the remaining carbons in the heterocyclic ring. The ^3^ *J*_C,H_ coupling of H-C(12) and H-C(11) to C6 and C2 as well as the ^3^ *J*_C,H_ coupling of H-C(11) and H-C(12) to each other clearly confirmed the positions of the quaternary carbon atom C1 and the two methyl groups. The methyl group at position C10 was determined by the correlation signals from H-C(10) to C8 and C9. The correlation signals of H-C(6) and H-C(4) to C13 and C5 indicated the position of the carboxylic acid at C5 (Figure S9A). Compound **45** corresponds to the proposed structure of 5-carboxyblumenol C 9-*O*-glucoside. The MS and NMR data (Figure S10) were in agreement with the literature.^66–68^ Compound **46** differed from 5-carboxyblumenol C 9-*O*-glucoside (**45**) by an additional double bond between C7 and C8. The olefinic protons were shifted toward higher ppm values compared to the saturated compound (Figure 3). The integral of the doublets of doublets and the HSQC indicated one proton each at C7 and C8, whereas 5-carboxyblumenol C 9-*O*-glucoside (**45**) showed two split signals of the geminal methylene protons H-C­(7α) and H-C­(7β). The coupling constant of H-C(7) and H-C(8) of ^3^ *J* ≈ 16 Hz demonstrated the presence of a trans double bond. Therefore, compound **46** was identified as 5-carboxydidehydroblumenol C 9-*O*-glucoside, which is described for the first time here.

**Figure 3:**
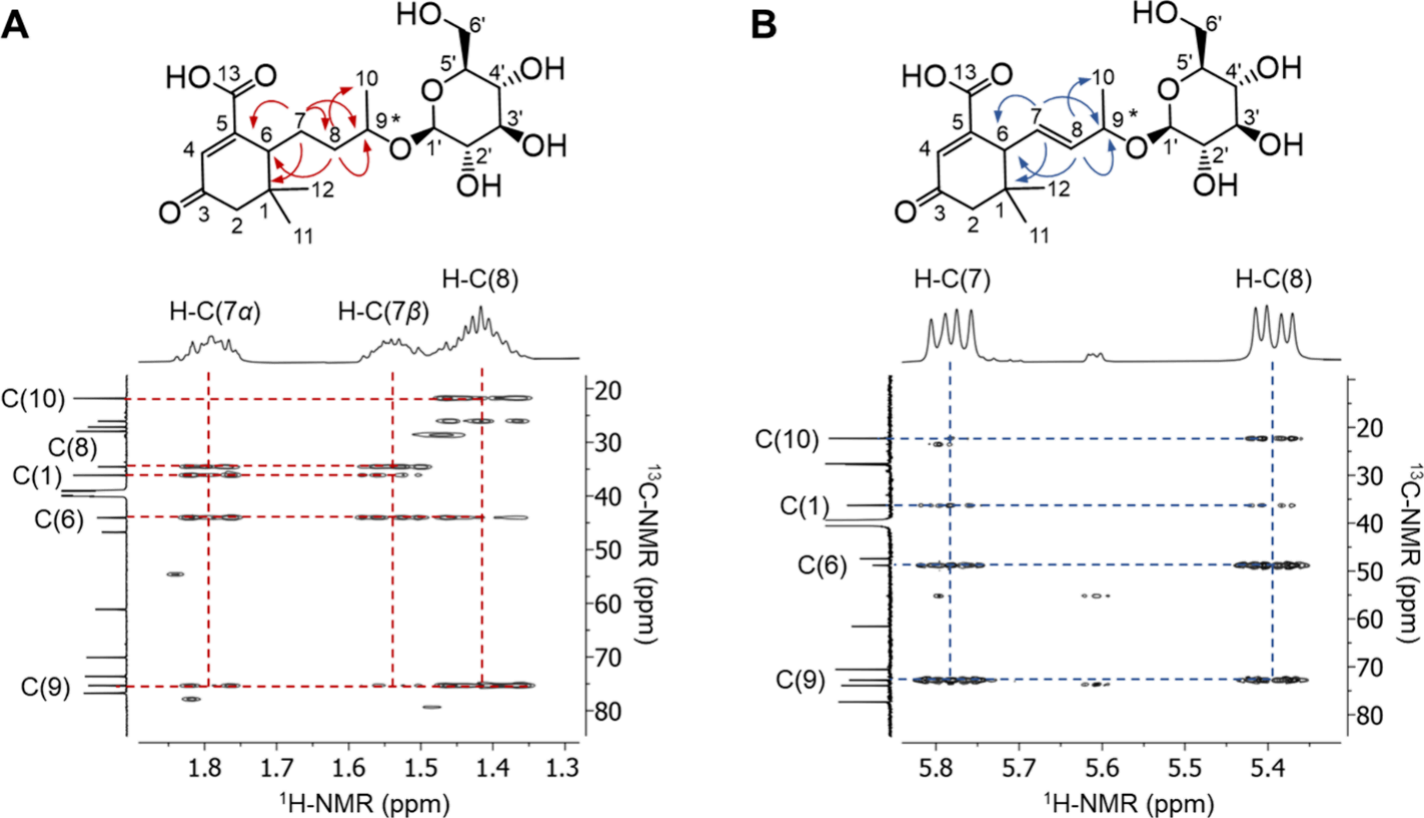
Excerpts of the HMBC (500/126 MHz, DMSO-*d*_6_, 300 K) spectra of (A) 5-carboxyblumenol C 9-*O*-glucoside (**45**) and (B) 5-carboxydidehydroblumenol C 9-*O*-glucoside (**46**). *Stereochemistry not defined.

Additionally, grasshopper ketone-3-sulfate (**47**) was isolated from barley leaves and fully characterized using UPLC-MS/MS and NMR spectroscopy. The compound ionized exclusively in negative ESI mode, showing an [M–H]^−^ ion of 303.0923 and the fragment ion *m/z of* 96.9590, indicating the cleavage of a sulfate group. Sulfate conjugation versus phosphorylation was assumed based on the accurate masses of the parent and fragment ions. The calculated exact mass of grasshopper ketone sulfate was 303.0902 (C_13_H_19_O_6_S), whereas that of grasshopper ketone phosphate was 303.0998 (C_13_H_20_O_6_P). The sulfate ion had a calculated mass of 96.9596 (HSO_4_^–^) and a phosphate mass of 96.9691 (H_2_PO_4_^–^). The ^1^H NMR spectra showed three singlets with an integral of three at 1.07, 1.27, and 1.32 ppm (isolated methyl groups), a three-proton singlet at 2.12 ppm (methyl ketone), a one-proton singlet at 5.75 ppm (olefinic proton), one hydroxy proton at 5.03 ppm, and two doublets of doublets of doublets (ddd) at 2.07 and 2.28 ppm (methylene groups). The ddd signals revealed coupling constants of 12 Hz corresponding to the geminal ^2^ *J*-coupling, 4 Hz, and 2 Hz, indicating the ^4^ *J*-coupling between the methylene protons at C2 and C4 (Table S12, Figure S11). The ^13^C NMR signal at 210 ppm is characteristic of the sp-hybridized carbon atom in allenes.^69^ The NMR data is in agreement with literature data on grasshopper ketone.^70–72^ Grasshopper ketone was first discovered in the defense secretion of the large flightless grasshopper^71^ and isolated from various plant species, including the *Poaceae* family (rice; refs 70,73–75 ). This is the first report of the presence of grasshopper ketone in barley and grasshopper ketone sulfate (**47**) in plants in general.

### Mass Spectrometry Imaging

Excising symptomatic leaf spots for LC-MS analysis leads to the destruction of cell compartmentalization and the loss of the heterogeneous distribution of stress metabolites within the leaf tissue. Desorption electrospray ionization mass spectrometry imaging (DESI-MSI) is a spatially resolved MS technique used to map the relative abundance of biomolecules in intact tissues. In contrast, DESI-MSI extracts metabolites exclusively on the leaf surface and has poorer selectivity due to its lack of chromatographic separation compared to LC-MS. Therefore, the combination of LC-MS and MSI represents a complementary solution to get a precise picture.

In the pathogen-induced local lesions, an upregulation of fatty acids, such as linoleic acid (**5**), and fatty acid oxidation products, such as hydroxy-, oxo-, and hydroperoxy derivatives of mainly C_18_ unsaturated fatty acids (**8**,**9**) were observed (Figure 4A). Downregulated substances were indicated by dark sections overlying the infection foci (Figure 4B), which revealed a reduction of saturated long-chain fatty acids in the pathogen-damaged areas. In addition, *p*-CA (**31**) and its oxidation products (**32**, **33**) showed higher abundances in the symptomatic spots (Figure S12). These findings were in agreement with the LC-MS analysis.

**Figure 4:**
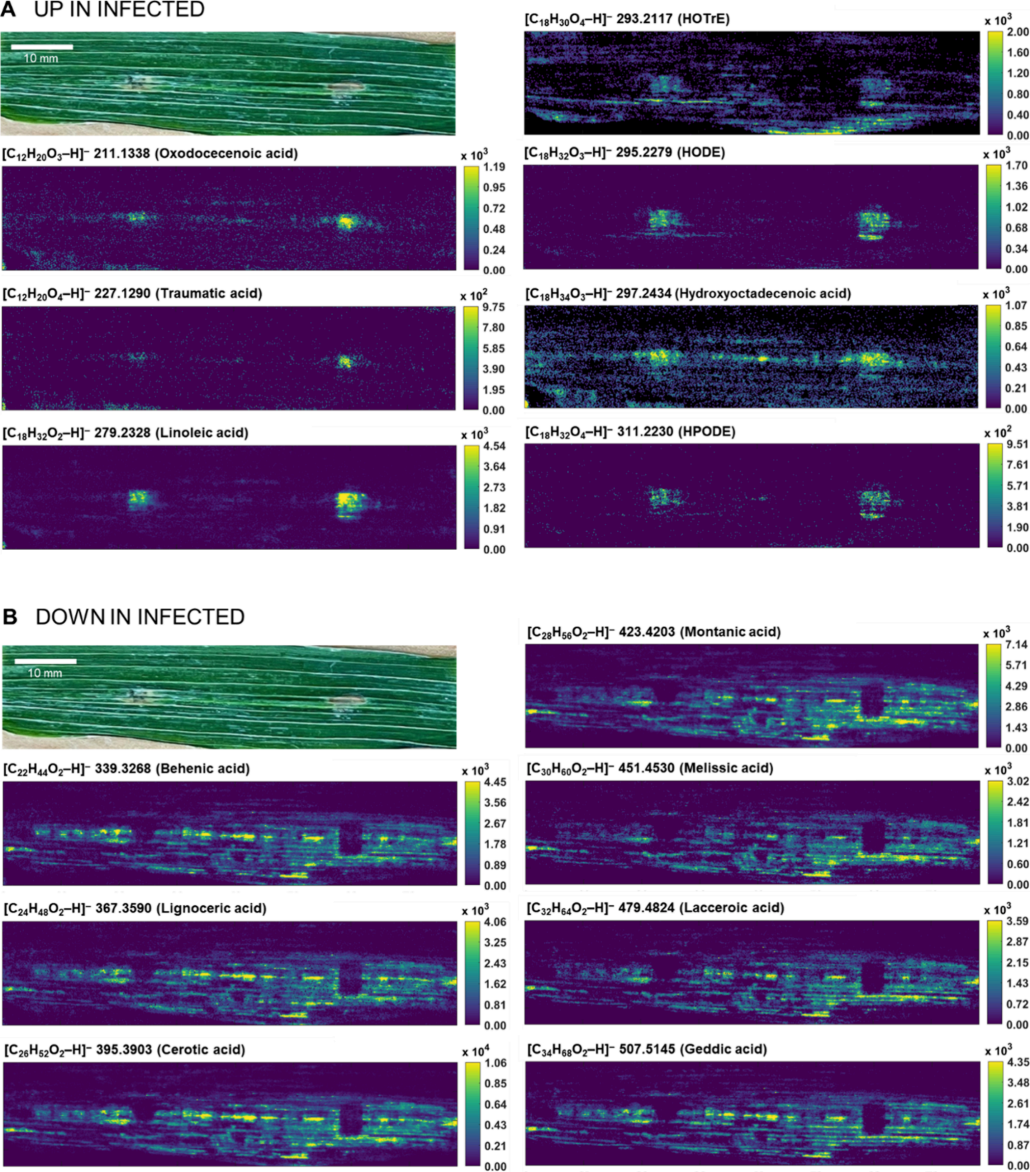
Optical image and DESI-MSI spectra of barley cultivar Golden Promise leaves with symptoms of spot blotch 7 days after infection with *B. sorokiniana* revealing (A) upregulated and (B) downregulated marker compounds.

### Genotype-Specific Regulation of Stress and Resistance Metabolites

Marker compounds in barley leaves related to spot blotch resistance were evaluated using untargeted metabolomics of HEB-25 lines previously found to be quantitatively resistant or susceptible to the leaf-infecting net blotch pathogen *Drechslera teres*,^76^ which follows a similar lifestyle as *B. sorokiniana* and is from the same family of ascomycete pathogens, the *Pleosporaceae*. Of the 29 genotypes that indicated quantitative resistance against *D. teres*, two highly resistant, two medium-resistant and three susceptible lines were selected to evaluate resistance marker compounds against *B. sorokiniana* (Figure S1). The metabolomes of quantitatively resistant (HEB_16_114, HID-219) and medium-resistant (HEB_06_049, parent line Barke) barley genotypes were compared to more susceptible lines (HID-069, HEB_06_154, and HID-386). PCA score plots clearly showed the distinction between resistant and susceptible genotypes, with the medium-resistant lines partially clustering between from both groups (Figure S13). The separation shows that the impact of the barley variety on the metabolome composition is greater than the inoculation with *B. sorokiniana*. Metabolites upregulated in the more resistant lines were associated with resistance (Table 2, Figure 5, substances **10**, **11**, **25**–**27**, **49**, **50**, **52**, **56**, **57**), whereas the substances with higher abundances in the susceptible lines were annotated as stress marker compounds (Figure 5, substances **4**–**9**, **15**, **16**, **32**, **33**, **35**, **45**–**47**, **51**, **53**–**55**).

**Figure 5:**
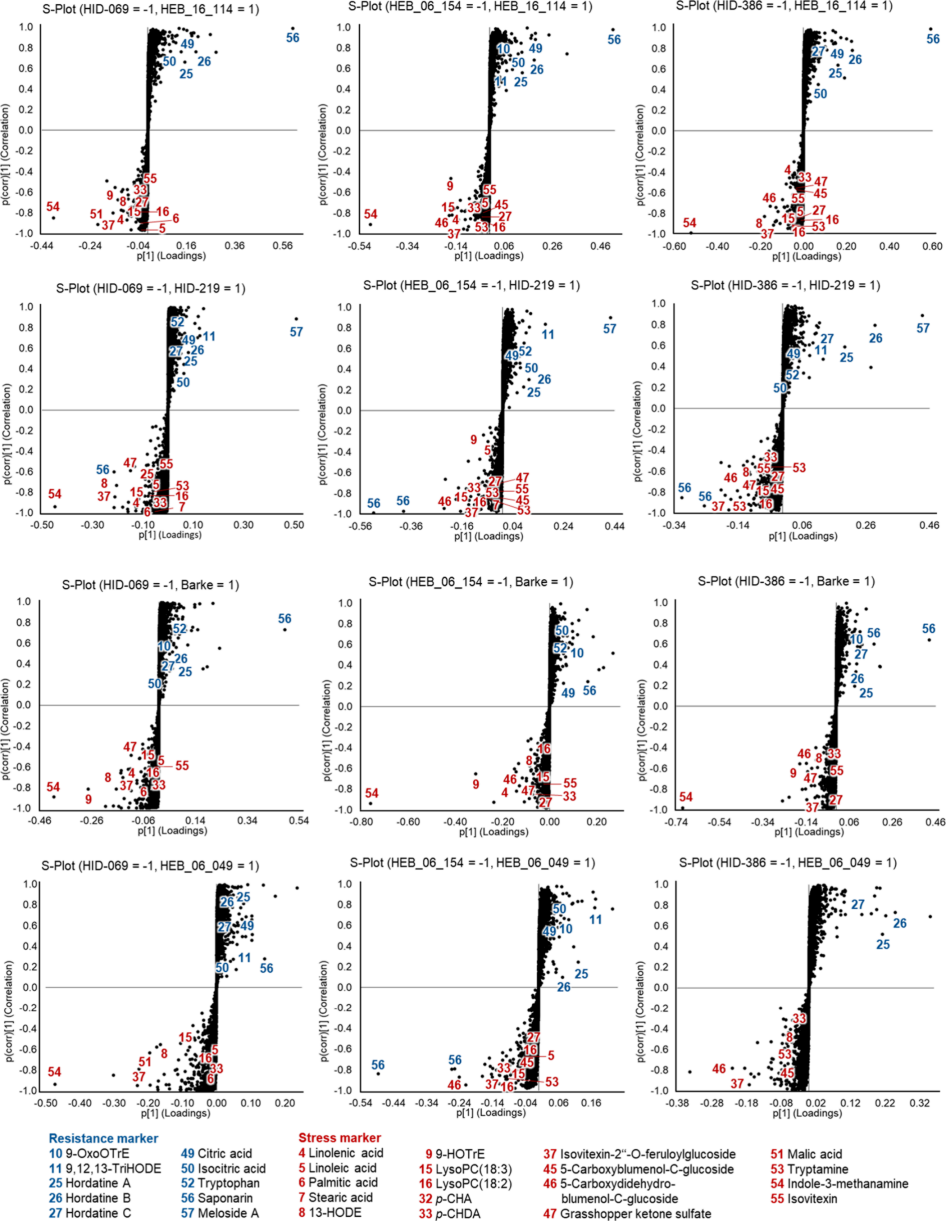
*S*-plots of different resistant (1) and susceptible (−1) barley genotypes of population HEB-25 and selected parents infected with *B. sorokiniana* and annotated resistance-related compounds (blue) and stress metabolites (red). Features were filtered by an ANOVA *p*-value ≤ 0.05 and a fold-change ≥ 2.

**Table 2:** Resistance- (**▲**) and Stress-Associated (**▼**) Metabolites in Barley Leaves Infected with *B. sorokiniana* ^a^

no	substance name	*m*/*z*	adduct	*m*/*z* fragments	*t*_R_ (min)	resistance/stress metabolite	ID level
Organic Acid							
**49**	citric acid	191.0191	[M-H]^−^	111.0088	1.37	**▲**	1
**50**	isocitric acid	191.0193	[M-H]^−^	173.0098	1	**▲**	1
				111.0088			
**51**	malic acid	133.0138	[M-H]^−^	115.0032	0.99	**▼**	1
				71.0134			
Indole derivatives							
**52**	tryptophan	188.0704	[M+H-NH_3_]^+^	166.0875	3.93	**▲**	1
		205.0971	[M+H]^+^	143.0715			
				130.0649			
				120.0803			
				103.0531			
				77.0386			
				70.0655			
**53**	tryptamine	144.0804	[M+H-NH_3_]^+^	143.0720	4.33	**▼**	1
		161.1073	[M+H]^+^	115.0542			
				77.0388			
**54**	indole-3-methanamine	130.0650	[M+H-NH_3_]^+^	118.0656	4.24	**▼**	1
				103.0535			
				77.0386			
Flavone glucosides							
**55**	isovitexin	431.1920	[M-H]^−^	341.0641	5.62	**▼**	1
				311.0554			
				283.0596			
**56**	saponarin (isovitexin 7-*O*-glucoside)	595.1664	[M+H]^+^	577.1555	5.07	**▲**	1
				433.1128			
				415.1022			
				397.0913			
				379.0812			
				337.0706			
				313.0704			
				283.0598			
				271.06			
				165.0178			
		593.1510	[M-H]^−^	473.1093			
				431.0983			
				311.0557			
				297.0395			
				282.0522			
				269.0443			
**57**	meloside A (isovitexin 2″-*O*-glucoside)	595.1670	[M+H]^+^	433.1136	5.34	**▲**	1
				415.1021			
				379.1981			
				337.0708			
				313.0709			
				283.0602			
				271.0610			
				165.0181			
		593.1507	[M-H]^−^	473.1073			
				413.0878			
				311.0553			
				293.0454			

a *m/z,* mass-to-charge ratio of precursor ion; *m/z* fragments, mass-to-charge ratio of MS^*2*^ fragment ions; *t*_R_, retention time; ID level, Identification level according to Metabolomics Standards Initiative:^171^ (1) identified compound using reference substances.

Several metabolites upregulated in infected HEB lines and parent HID or Barke were higher in the more susceptible genotypes. For example, malic acid (**51**) and indole-3-methanamine (**54**) were identified as stress marker compounds in less resistant lines. In the more resistant lines, hordatines (**25**–**27**), flavone glucosides (**56**, **57**), tryptophan (**52**) oxo and trihydroxy fatty acids (**10**, **11**) citric acid (**49**), and isocitric acid (**50**) were upregulated (Figure 5).

### Antifungal Activity of Marker Compounds

The antifungal activity of selected marker metabolites (**4**, **5**, **16**, **25**–**30**, **35**, **49**, **51**–**56**) and structural analogues that were available in sufficient quantities was tested in a 96-well-plate-based bioassay against *B. sorokiniana*. The tested flavone glucosides as well as their aglycone apigenin promoted fungal growth of *B. sorokiniana* (Figure 6). A mixture of hordatine A, B, and C (**25**–**27**) as well as a mixture of their corresponding glucosides (**28**–**30**) was isolated from barley leaves, tested in their natural composition and revealed a minor growth-inhibiting effect on *B. sorokiniana*. The other tested compounds, like hydroxycinnamic acids, fatty acids (**4**, **5**) organic acids (**49**, **51**) and tryptophan metabolites (**52**–**54**) significantly inhibited mycelial growth of *B. sorokiniana*. Grasshopper ketone showed the strongest inhibition compared to the solvent control (67%).

**Figure 6:**
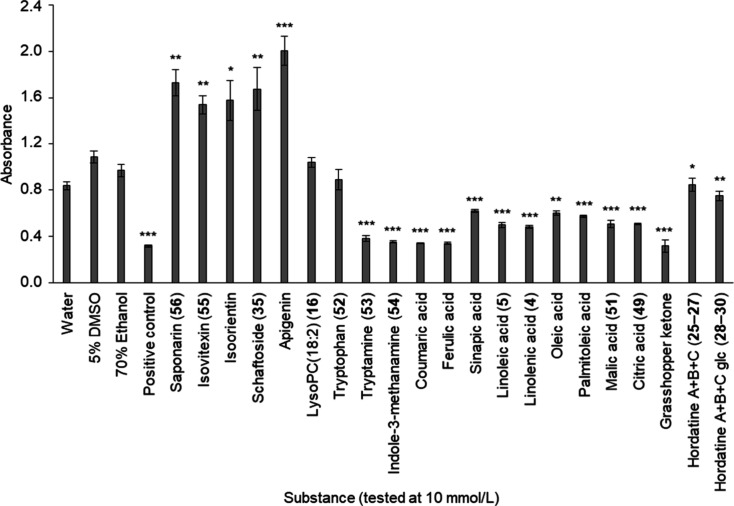
Antifungal activity of selected marker metabolites against *B. sorokiniana*. The inhibition was referred relative to the respective solvent in which the substance was dissolved. Hexanoic acid was used as a positive control (*p < 0,05; **p < 0,01; ***p < 0,001; *n* = 4; unpaired *t*-test).

## Discussion

### Fungal Infection Influences Linolenate Metabolism

The lipidomics analysis of susceptible cv. Golden Promise highlighted the linolenic acid metabolism as a key pathway in the defense response of barley against the spot blotch pathogen *B. sorokiniana*. The differences in the fatty acid composition of the whole infected leaf and the spots indicated local, leaf-systemic responses of the plant against the pathogen. The polyunsaturated fatty acids linolenic acid (**4**) and linoleic acid (**5**) were upregulated in the infected tissue around the spots, whereas fatty acid oxidation products (**8**–**10**) and the saturated fatty acids palmitic acid (**6**) and stearic acid (**7**) were more abundant within the symptomatic areas. In the spots, the presence of fatty acid oxidation products (**8**–**10**) and antioxidants such as glutathione (**43**), hordatines (**25**–**27**) and flavone glucosides (**34**–**37**) could be observed as signs of cell death reactions, characterized by an oxidative stress leading to lipid peroxidation.^77,78^ This response occurs nonenzymatically through the action of reactive oxygen species (ROS) or is catalyzed by enzymes such as lipoxygenases (LOX). Linolenic acid and linoleic acid are the most common substrates of LOX in plants, whereby linolenic acid is converted more efficiently than linoleic acid by chloroplast lipoxygenase.^79^ These are metabolized to the corresponding 9- and 13-hydroxy fatty acids^80^ and further converted into oxo fatty acids by LOX, hydroxy fatty acids through the action of reductases or glutathione, or into volatile aldehydes by hydroperoxide lyase.^34,80,81^ Those volatiles, known as green leaf volatiles, act as signaling molecules in interactions between neighboring plants or distinct plant organs.^82,83^ Pre-exposure of barley to green leaf volatiles triggered immunity against fungal infection by the upregulation of hordatines, unsaturated fatty acids and linolenate-conjugated lipids.^24^ In addition, linolenic acid (**4**) was reported to act as an antifungal against *Rhizoctonia solani* and *Crinipellis perniciosa* ^84^ and to activate NADPH oxidase for the production of ROS,^85,86^ which further elevates hypersensitive defense reactions.

In the green leaf tissue surrounding the symptomatic areas, leaf-systemic reactions were predominant with the accumulation of precursor substances, such as unsaturated fatty acids, and protective secondary metabolites, such as coumaroylagmatine derivatives (**31**–**33**) and blumenol C glucosides (**45**, **46**).

Different linolenate-conjugated lipids (**12**–**18**) were upregulated as a result of the infection of barley with *B. sorokiniana* in both symptomatic spots and the surrounding green leaf tissue. Storage lipids, such as triglycerides (**17**, **18**), are a potential source of energy, provided by β-oxidation, that is needed for stress survival or recovery. To enter β-oxidation, fatty acids released from membrane lipids are initially deposited in triglycerides to protect the plant from cytotoxic free fatty acids.^87^ In addition, unsaturated fatty acids can be released for the biosynthesis of signaling molecules such as jasmonates.^88^ In contrast, polar lipid components with linolenate side chains (**19**–**21**) were downregulated by the infection. MGDG and DGDG, the polar lipid constituents of the thylakoid membrane, are exclusively biosynthesized in chloroplasts and play an essential role in numerous biochemical pathways facilitating photosynthesis, light reactions, and chloroplast morphology.^89–92^ Structures with two linolenic acid side chains (18:3/18:3) are most common.^93^ The depletion of linolenate-conjugated lipids (**12**–**18**) coupled with the upregulation of free linolenic acid (**4**) could explain the release of unsaturated fatty acids from membrane lipids.

Acyl-MGDG (**22**–**24**) are formed by esterification of MGDG at the 6′-position of galactose with another fatty acid, induced by mechanical wounding, sublethal freezing, or bacterial infection.^41,94^ Nilsson et al. reported the accumulation of acyl-MGDG (**22**–**24**) in *N. benthamiana* following hypersensitive cell death triggered by effectors secreted by the bacterium *Pseudomonas syringae* .^41^ There is evidence that MGDG acylation is catalyzed by an acyl transferase during stress^41^ and that DGDG acts as an acyl donor.^94^ One potential biological function of acyl-MGDG (**22**–**24**) could be to act as a reservoir for signaling compounds. However, jasmonic acid production was not linked to acyl-MGDG (**22**–**24**) accumulation. Therefore, acyl-MGDG (**22**–**24**) might sequester potentially harmful fatty acids from the main membrane lipid pool instead.^94^

### Infection with *B. sorokiniana* Triggers the Accumulation of Defense-Associated Metabolites

Hordatine A and B (**25**, **26**) and their corresponding glucosides (**28**, **29**) were downregulated in leaves of barley *cv.* Golden Promise after infection with *B. sorokiniana*. Hordatines are described as antifungal compounds against different pathogenic fungi, such as *Botrytis allii*, *Colletotrichum coccodes*, *Fusarium solani*, *Glomerra cingulate*, *Helminthosporium sativum*, and *Monilinia fructicola*.^95,96^ Their biosynthetic precursor, *p*-CA (**31**) was downregulated in the whole leaf and slightly upregulated in necrotic spots. This observation suggests *p*-CA (**31**) as a marker metabolite for different stages of infection. In contrast, the oxidation products *p*-CHA (**32**) and *p*-CHDA (**33**) were upregulated. *p-*CHA (**32**) is induced in barley by phytohormones, such as jasmonic acid and abscisic acid, and during osmotic stress.^52^ *p*-CHDA (**33**) appears to be a nonenzymatic oxidation product of *p*-CHA (**32**).^97^ Both compounds (**32**, **33**) are described acting as antifungals in barley against the powdery mildew fungus *Blumeria hordei* potentially lowering its penetration success. *p*-CHDA (**33**) was shown to be more potent than *p*-CHA,^32^ while *p*-CA (**31**) showed no inhibitory effect.^51,97^ *p*-CA, p-CHA, and *p*-CHDA (**31**–**33**) belong to the substance class of hydroxycinnamic acid amides (HCAA). HCAAs contribute to stress tolerance in numerous plant species due to their high antioxidant activity,^98^ cross-linking of cell wall structures,^99^ or direct antifungal activity.^54,100^ The exact biological function of the barley-specific hordatines (**25**–**27**) whether they are directly involved in pathogen defense, or if effects are mediated by the oxidation products of their precursors or integration into the cell wall, remains unknown.

The cyanoglucosides epiheteroendrin (**38**), sutherlandin (**39**), osmaronin (**40**), dihydroosmaronin (**41**), and epidermin (**42**) are derived from leucine and are present in a specific ratio in almost every barley line.^22^ Epiheterodendrin (**38**) the only α-hydroxynitrile glucoside, has the potential to release toxic hydrogen cyanide after enzymatic cleavage.^101^ However, no β-d-glucosidase is present in the *Hordeum vulgare* leaf epidermis, where the cyanoglucosides specifically accumulate.^102^ The noncyanogenic β- and γ-hydroxynitrile glucosides (**39**–**42**) have been reported to act defensively against pathogens in barley leaves.^22^ As toxic HCN was not released, their protective effect cannot be explained via this mechanism. Alternatively, the intact glucosides may inhibit fungal growth. The exact biological function of noncyanogenic glucosides (**39**–**42**) remains unknown, but their role in nitrogen storage has been discussed.^101,103^

Several flavone di- and triglucosides (**34**–**36**) were downregulated, whereas conjugates with hydroxycinnamic acids (HCA, **37**) were upregulated in the infected leaves. HCA-conjugated flavones are known stress-induced metabolites that increase in barley under drought stress^104^ or nutrient deficiency.^105^ They possess antioxidant effects, show radical-scavenging activity, and prevent the photooxidation of vitamins.^106^

Apocarotenoids are a class of carotenoid oxidation products biosynthesized enzymatically by the cleavage of carotenoids or by exposure to ROS^107^ and act as stress regulators in plants.^107–111^ Grasshopper ketone (**x**) is a degradation product of neoxanthin and fucoxanthin^112^ with an allene structure. Allene structures are known for their antifungal activity.^113^ Blumenol C glucosides have been reported to accumulate in barley during drought stress^104^ and as fungus-induced metabolites in barley roots colonized by mycorrhizal fungi.^66–68,114–116^ However, 5-carboxyblumenol C glucoside (**45**) has not yet been identified in barley leaves. In rice, blumenol A and grasshopper ketone have been described as allelopathic substances inhibiting weed growth.^70^ The exogenous application of blumenol C 2″-O-glucuronylglucoside (blumenin) to barley roots inhibits fungal colonization and is negatively correlated with the amount of *p*-CA (**31**) and coumaroylputrescine in mycorrhizal barley roots.^117^ Here, we present for the first time the accumulation of this compound class (**45**–**48**) in barley leaves infected with a phytopathogenic fungus, which suggests that they might act as defense substances in the plant’s immune response.

### Site-Specific Regulation of Marker Metabolites

The plant cuticle represents a physical barrier that protects the leaf from biotic and abiotic stresses. It is composed of polymeric cutin and solvent-extractable cuticular waxes.^118^ Naturally occurring cuticular waxes contain long-chain fatty acids with an even number of carbon atoms.^119^ Pathogen invasion leads to the destruction of the protective wax layers on the leaf surface. The biosynthesis of cuticular waxes starts with the de novo synthesis of C_16_ or C_18_ fatty acids, followed by the extension to very-long-chain fatty acids, which are direct precursors for wax synthesis.^120^ The significantly higher amount of C_16_ and C_18_ fatty acids (**6**, **7**) in the symptomatic spots observed by LC-MS/MS lipidomics analysis suggests an upregulation of cuticular wax biosynthesis to compensate for the loss of long-chain fatty acids caused by pathogen invasion.

During environmental stress, unsaturated fatty acids are released from membrane lipids and oxidized by lipoxygenases or nonenzymatically by the action of ROS. The resulting hydroperoxides can undergo a variety of secondary reactions (Figure S14). They are reduced by glutathione peroxidase and react to hydroxy-, epoxy-, oxo-, and divinylether fatty acids, or jasmonates. Hydroperoxide lyase (HPL) cleaves the hydroperoxides into volatile aldehydes (green leaf volatiles) and 12-oxo-(9*Z*)-dodecanoic acid. After isomerization, 12-oxo-(9*Z*)-dodecanic acid is converted into 12-oxo-(10*E*)-dodecanic acid (traumatin), which is subsequently oxidized as a result of nonenzymatic autoxidation into traumatic acid.^121–123^ We found several intermediates and products of the LOX pathway upregulated in the symptomatic spots, which suggests a potential role as a defense pathway against *B. sorokiniana*.

### Resistance-Related Marker Compounds for the *B. sorokiniana*-Barley Interaction

The more resistant HEB-25 genotypes revealed increased levels of saponarin (isovitexin-7-O-glucoside) compared to the more susceptible genotypes. Saponarin (**56**) is the major flavone of barley primary leaves. Its accumulation has been observed in response to different environmental stresses, such as UV radiation,^124^ high temperature,^49^ drought^125^ and mechanical stress.^126^ A protective function of saponarin (**56**) against toxic oxygen radicals generated in stressed plant tissues has been discussed.^55^ In addition, flavone O-glucosides have been associated with the resistance of barley against *Fusarium graminearum*.^127,128^ The wild barley accession HID-219 was the only line examined that exhibited an isomer of saponarin, identified as meloside A (**57**). A similar decrease in saponarin (**56**), along with an increase in meloside A (**57**) was investigated in the developmental process of the first and third leaves of barley seedlings of the high-yield barley cultivar Scarlett.^56^

In contrast, an upregulation of isovitexin (**55**) and HCA-conjugated flavones (**37**) in the susceptible barley lines was observed in this study. Ishihara et al. (2002) described a similar increase in sinapoylsaponarin accompanied by a decrease in saponarin in barley leaves after treatment with the stress-related hormone jasmonic acid.^55^ Abou-Zaid et al. observed an accumulation of isovitexin (**55**) and its HCA-conjugates in *Cucumis sativus* leaves treated with silicon and infected with *Sphaerotheca fuliginea*.^129^ These findings suggest that deglycosylation and the HCA-conjugation of flavone glucosides are major stress response reactions of barley.

Oxo and trihydroxy fatty acids (**10**, **11**) were indicative of resistance, while fatty acids (**4**–**7**) and hydroxy oxylipins (**8**, **9**) demonstrated a stress-related condition. Trihydroxy oxylipins (**11**) were found to reduce fungal spore germination and were associated with resistance against *Uromyces fabae* in bean plants.^84,130^ Oxo-fatty acids (**10**) were reported as resistance metabolites in barley against *Fusarium graminearum*.^131^

L-tryptophan (**52**) was upregulated in the more resistant barley genotypes, whereas L-tryptamine (**53**) and indole-3-methanamine (**54**) were present in significantly higher amounts in the susceptible cultivars. The exogenous application of L-tryptophan (**52**) has been described to increase growth and yield of healthy plants^132,133^ and to enhance abiotic stress tolerance.^134,135^ These protective effects have been attributed to the role of L-tryptophan (**52**) as a precursor of the plant growth-regulating hormone auxin (indole-3-acetic acid;^134^ Tryptamine (**53**) accumulation has been described in UV-irradiated and pathogen-inoculated barley leaves.^136,137^ Indole-3-methanamine (**54**) is a precursor in the biosynthesis of the indole alkaloid gramine.^138^ Gramine is one of the oldest known bioactive metabolites in barley wild types, possessing an allelopathic inhibitory effect on weed growth and a toxic effect on herbivores, insects and pathogens, including *B. sorokiniana*, and is associated with resistance to powdery mildew.^139–144^ During domestication gramine production was lost.^145,146^ Cultivars like Barke are considered as nongramine-producers.^138^

In addition, citric and isocitric acid (**49**,**50**) were more abundant in the resistant genotypes, whereas malic acid was enriched in the susceptible lines. These intermediates of the tricarboxylic acid cycle (TCA) imply the involvement of energy metabolism in the stress response of barley against fungal infection. It has been reported that the ratio of citric (**49**) to malic acid (**51**) is greater in stressed plants.^147^ In barley, there are conflicting observations about TCA cycle up- and downregulation in response to abiotic stress. An upregulation has been observed in barley grain under drought stress conditions.^148,149^ In salt-stressed barley leaves, the TCA cycle was downregulated, which has been explained by decreased respiration/energy usage due to reduced growth.^150^

Lysophosphocholines (LysoPC, **15**,**16**) with unsaturated fatty acids were more abundant in the susceptible barley genotypes compared to the resistant ones. In biological membranes, PCs are the most abundant glycerophospholipids containing unsaturated acyl chains sensitive to oxidation. Oxidized chains are removed from damaged membrane lipids by phospholipase A, which hydrolyzes phospholipids into LysoPC and free fatty acids.^151^ The role of LysoPC as signaling lipids in direct stress signal transduction has been described.^152^ It has been reported that LysoPC levels increased in tobacco leaves after inoculation with *Phytophthora parasitica*, and that pathogen-induced LysoPC enhanced pathogen susceptibility accompanied by ROS formation.^153,154^

Based on the untargeted metabolome analysis of selected lines of the HEB-25 population, it was possible for the first time to identify metabolic marker substances in barley leaves associated with resistance to *B. sorokinana*. To conclusively determine that the metabolites are responsible for the resistance of the barley lines studied, experiments with transgenic mutants are required in which individual biosynthetic pathways leading to the formation of the identified resistance metabolites are silenced or overexpressed. In the future, the marker metabolites could be used in the screening of other barley lines to assess their resistance behavior to *B. sorokiniana*. The marker metabolites can be associated with corresponding resistance-conferring mQTL to enable resistance screening of other barley cultivars at both genetic and molecular level. Once the biosynthesis pathways of the marker metabolites have been elucidated, the genes and QTL encoding the resistance metabolites can be enriched in new, resistant barley lines using genetic engineering techniques, enabling the targeted breeding of genotypes with improved biotic tolerance traits. However, prior to this, follow-up studies should examine various combinations of stressors that could have an additional influence on the resistance traits of the HEB-25 NAM population to rule out potential interactions and side effects.

### Antifungal Activity of Marker Compounds

The tested flavone glucosides and aglycones (**35**, **55**, **56**) promoted fungal growth of *B. sorokiniana*. A similar effect of flavonoids stimulating germination and fungal growth of pathogenic *Fusarium* species has been reported.^155,156^ Furthermore, flavonoids in root exudates of carrot or *Eucalyptus* stimulated hyphal development of mycorrhizal fungi.^157,158^ Therefore, the upregulation of flavone glucosides (**56**, **57**) in the resistant barley cultivars could not be explained by an antifungal activity of these compounds, but might protect the plant due to their antioxidant properties.

Hordatines (**25**–**27**) and hordatine glucosides (**28**–**30**) only had a minor growth-inhibiting effect on *B. sorokiniana*, although they have long been considered as antifungals in barley against various pathogens. The only study to date on the antifungal activity of hordatines (**25**, **26**) investigated their inhibitory effect on spore germination instead of fungal growth.^159^ Therefore, the plant-protective and resistance-mediating effects of hordatines (**25**–**27**) might be due to their ability to strengthen plant cell walls rather than to direct antifungal properties.

Hydroxycinnamic acids, fatty acids (**4**, **5**), organic acids (**49**, **51**), and tryptophan metabolites (**52**–**54**) significantly inhibited mycelial growth of *B. sorokiniana*. Coumaric acid has been reported to inhibit the growth of *Botrytis cinerea* and *Penicillium expansum*.^160,161^ Ferulic acid and sinapic acid have been described as antifungal against *Fusarium graminearum* and *Candida albicans* .^162,163^ The organic acids citric acid (**49**) and malic acid (**51**) showed an inhibitory effect against phytopathogenic fungi such as *Colletotrichum* sp.^164^ and *Monilia fructigena*.^165^ Another study revealed that organic acids suppress the growth of *Aspergillus flavus*, *Penicillium purpurogenium*, *Rhizopus nigricans*, and *Fusarium oxysporum* and reduce mycotoxin production.^166^ Unsaturated fatty acids act antifungal against the plant pathogens *Rhizoctonia solani*, *Pythium ultimum*, *Pyrenophora avenae*, *Crinipellis perniciosa*, *Alternaria solani*, *Colletotrichum lagenarium*, and *Fusarium* sp.^84,167^ Tryptamine (**53**) showed fungicidal activity against *Aspergillus* sp.,^168,169^ and the soybean pathogens *Cercospora kikuchii*, *Cercospora sojina*, *Septoria glycines*, and *Sclerotium rolfsii*.^170^ Therefore, the upregulation of fatty acids (**4**, **5**), organic acids (**49**–**51**), and tryptamine (**53**) in the analyzed resistant barley lines might contribute to plant defense.

The results of the untargeted metabolome and lipidome analyses provide insights into the metabolic pathways that are up- or downregulated by *B. sorokiniana* infection in barley leaves. The identification of marker metabolites associated with biotic stress could be used to detect fungal infections in barley leaves at the molecular level. This could be supplemented by genetic markers after identifying the gene segments and QTL encoding these metabolites. These markers can be used by farmers and researchers to detect fungal infections early, before visible damage occurs. This enables rapid response and targeted measures to contain the spread of the disease. In addition, the understanding of metabolic changes can be used in breeding programs to develop barley varieties with increased resistance to fungal infections. By specifically upregulating plant defense mechanisms at the metabolic level, strategies can be developed to strengthen plants’ natural resistance and limit or the use of synthetic fungicide.

However, it remains to be clarified whether the metabolites were endogenously present in the plant and up-/downregulated by the infection, or whether their origin was partly fungal. This could be achieved using labeling experiments. The functional role of individual lipids and metabolites in the defense response can be investigated in experiments with transgenic barley varieties with modified biosynthetic pathways, for example, by overexpressing inhibitors or biosynthetic enzymes.

## Conclusions

In this study, the metabolic responses of barley leaves to infection with *B. sorokiniana* were analyzed using untargeted metabolomics and lipidomics. Marker metabolites were selected using PCA and OPLS-DA and identified using different techniques such as cochromatography with reference substances, preceding isolation or synthesis, NMR experiments, and UPLC-TOF-MS^2^. The local distribution of marker metabolites within the leaf tissue was determined using mass spectrometry imaging. The analysis of different quantitatively resistant and susceptible barley genotypes was used to find potential resistance-related marker compounds. Our study confirmed the hypothesis that fungal infection induces alterations in the plant metabolome and that resistant barley varieties react to biotic stress with an upregulation of defense-related secondary metabolites. These results could provide insight into the metabolic pathways involved in the defense reactions of plants to biotic stress challenges. The identified marker metabolites may serve as biomarker molecules in targeted studies to detect pathogen attacks. Moreover, these naturally occurring likely protective chemicals could be genetically enriched in breeding programs for disease resistance to replace or complement the use of synthetic fungicides in the prevention of crop losses.

## Supplementary Material



## Notes

Detailed method description of UHPLC-TOF-MS-based metabolomics analysis; disease score rating of the barley genotypes of the NAM population; ESI-TOF-MS^2^, 1D- and 2D NMR data of identified compounds; DESI-MSI spectra of phenolamides in infected barley leaves; PCA score plots of different resistant and susceptible barley genotypes infected with *B. sorokiniana*; and scheme of the lipoxygenase pathway (DOCX)

## Abbreviations

COSY: correlation spectroscopy
DESI-MSI: desorption electrospray ionization mass spectrometry imaging
DGDG: digalactosyldiacylglycerol
FA: formic acid
glc: glucoside
Hac: acetic acid
HCA: hydroxycinnamic acid
HCAA: hydroxycinnamic acid amides
HEB-25: halle exotic barley
HMBC: heteronuclear multiple-bond correlation
HODE: hydroxyoctadecadienoic acid
HPODE: hydroperoxyoctadecadienoic acid
HPOTE: hydroperoxyoctadecatrienoic acid
HPLC: high-performance liquid chromatography
HR: hypersensitive response
HSQC: heteronuclear single quantum coherence
LC-MS: liquid chromatography coupled to mass spectrometry
LOX: lipoxygenase
MGDG: monogalactosyldiacylglycerol
MPLC: medium-pressure liquid chromatography
MS^2^: tandem mass spectrometry
*m*/*z*: mass to charge ratio
NMR: nuclear magnetic resonance spectroscopy
HOTrE: hydroxyoctadecatrienoic acid
OPLS-DA: orthogonal partial least-squares discriminant analysis
OxoOTrE: oxooctadecatrienoic acid
PC: phosphatidylcholine
PCA: principal component analysis
*p*-CHDA: *p*-coumaroyl-3-hydroxydehydroagmatine
*p*-CHA: *p*-coumaroyl-3-hydroxyagmatine
*p*-CA: *p*-coumaroylagmatine
ppm: parts per million
QTL: quantitative trait loci
TG: triacylglycerol
TriHODE: trihydroxyoctadecadienoic acid
t_R_: retention time
UPLC-TOF-MS: ultra high-performance liquid chromatography coupled to mass spectrometry with time-of-flight mass analyzer
