# Supplementary material for: UPLC-ESI-TOF-MS Profiling of Metabolome Alterations in Barley (Hordeum vulgare L.) Leaves Induced by Bipolaris sorokiniana
Source: J Agric Food Chem. 2025 Sep 18;73(39):24662–87. doi: 10.1021/acs.jafc.5c05419 (PMC12492401; doi:10.1021/acs.jafc.5c05419)
Supplement: Supplementary file 1 [file jf5c05419_si_001.docx]

Supporting information

**UPLC-ESI-TOF-MS profiling of metabolome alterations in barley (*Hordeum vulgare* L.) leaves induced by *Bipolaris sorokiniana***

Lisa Kurzweil,^1^ Timo D. Stark,^2^ Karina Hille,^2^ Felix Hoheneder,^3^ Jana Mrtva,^2^ Hans Hausladen,^4^ Miriam Lenk,^5^ Mohammed S. Motawia,^6^ Nicole Strittmatter,^8^ A. Corina Vlot,^5,9^ Klaus Pillen,^10^ Mette Sørensen,^6,7^ Birger L. Møller,^6^ Ralph Hückelhoven,^3^ Corinna Dawid,^1,2,11^*

^1^ Professorship for Functional Phytometabolomics, TUM School of Life Sciences, Technical University of Munich, Lise-Meitner-Str. 34, 85354 Freising, Germany.

^2^ Chair of Food Chemistry and Molecular Sensory Science, TUM School of Life Sciences, Technical University of Munich, Lise-Meitner-Str. 34, 85354 Freising, Germany.

^3^ Chair of Phytopathology, TUM School of Life Sciences, Technical University of Munich, Emil-Ramann-Str. 2, 85354 Freising, Germany.

^4^ Plant Technology Center, TUM School of Life Sciences, Technical University of Munich, Dürnast 9, 85354 Freising, Germany.

^5^ Institute of Biochemical Plant Pathology, Helmholtz Zentrum München, Ingolstädter Landstraße 1, 85764 Neuherberg, Germany.

^6^ Department of Plant and Environmental Sciences, University of Copenhagen, Thorvaldsensvej 40, 1871 Frederiskberg C, Copenhagen, Denmark.

^7^ Novo Nordisk Pharmatech, Københavnsvej 216, 4600 Køge, Copenhagen, Denmark.

^8^ Professorship for Analytical Chemistry, Technical University of Munich, Lichtenbergstr. 4, 85748 Garching, Germany.

^9^ Chair of Crop Plant Genetics, Faculty of Life Sciences: Food, Nutrition and Health, University of Bayreuth, Fritz-Hornschuch-Straße 13, 95326 Kulmbach, Germany.

^10^ Chair of Plant Breeding, Martin-Luther-University Halle-Wittenberg, Betty-Heimann-Str. 3, 06120 Halle (Saale), Germany.

^11^ Leibniz Institute for Food Systems Biology at the Technical University of Munich, Lise-Meitner-Str. 34, 85354 Freising, Germany.

E-mail: [lisa.kurzweil@tum.de](mailto:lisa.kurzweil@tum.de), [timo.stark@tum.de](mailto:timo.stark@tum.de), [karina.hille@tum.de](mailto:karina.hille@tum.de), [jana.mrtva@tum.de](mailto:jana.mrtva@tum.de), [felix.hoheneder@tum.de](mailto:felix.hoheneder@tum.de), [hans.hausladen@tum.de](mailto:h.hausladen@tum.de), [miriam.lenk@helmholtz-muenchen.de](mailto:marion.wenig@helmholtz-muenchen.de), [corina.vlot@uni-bayreuth.de](mailto:corina.vlot@uni-bayreuth.de), [klaus*.*pillen*@*landw.uni-halle.de](mailto:klaus.pillen@landw.uni-halle.de), [mfqn@novonordisk.com](mailto:mfqn@novonordisk.com), [mosm@plen.ku.dk](mailto:mosm@plen.ku.dk), [nicole.strittmatter@tum.de](mailto:nicole.strittmatter@tum.de), [blm@plen.ku.dk](mailto:blm@plen.ku.dk), [hueckelhoven@tum.de](mailto:hueckelhoven@tum.de), [corinna.dawid@tum.de](mailto:corinna.dawid@tum.de)

* **Corresponding author:**

Phone: +49 8161 712902; Fax: +49 8161 712949

E-mail: [corinna.dawid@tum.de](mailto:corinna.dawid@tum.de)

## **Methods**

The sample preparation and analysis method was modified according to Giavalisco et al. (2011). 100 mg frozen leaf tissue was homogenized in 2 mL bead beater tubes (Bertin Technologies, Montingny-le-Bretonneux, France) filled with ceramic balls (zirconium oxide; mix beads of 1.4 and 2.8 mm) with 1 mL cold methanol/*tert*-butyl methyl ether/water 1/3/1 (v/v/v) in the Precellys^®^ homogenizer (Bertin Technologies, Montingny-le-Bretonneux, France; 6500 rpm, 3 × 30 s, 15 s pause). The supernatant was removed and stored in the fridge, and the extraction was repeated for three cycles. 3250 µL of methanol/water 1/3 (v/v) was added to the combined extract, homogenized, and centrifuged (5 min, 4 °C) to achieve phase separation. The three phases – the upper organic phase containing lipids, the lower aqueous phase containing metabolites, and a pellet of starch and proteins at the bottom – were isolated, dried under nitrogen and dissolved in 150 µL water (aqueous extract) or 500 µL acetonitrile/2-propanol 7/3 (v/v) (lipid extract).

The aqueous extract was used for the analysis of secondary metabolites. Chromatographic separation was achieved on an ACQUITY UPLC^®^ I-Class system (Waters, Eschborn, Germany) with an ACQUITY UPLC-BEH C18 column (150 × 2.1 mm × 1.7 µm, Waters, Eschborn, Germany) as the stationary phase and a mobile phase consisting of 0.1% formic acid in water (eluent A) and 0.1% formic acid in acetonitrile (eluent B). The mobile phase composition changed as follows: hold 1% B for 1 min, 6 min linear gradient from 1% B to 35% B, 1 min linear gradient from 35% B to 70% B, 1 min linear gradient from 70% B to 99% B, hold 99% B for 3 min, 0.5 min linear gradient from 99% B to 1% B, and hold 1% B for 1 min with a flow rate of 0.4 mL/min, autosampler temperature of 10 °C, column temperature of 45 °C, and injection volume of 3 µL.

The lipid extract was used for lipidomics analysis on a C_8_ column (100 × 2.1 mm × 1.7 µm, Phenomenex, Aschaffenburg, Germany) and a mobile phase of water with 1% 1 M ammonium acetate and 0.1% acetic acid (eluent A) and acetonitrile/2-propanol 7/3 (v/v) with 1% 1 M ammonium acetate and 0.1% acetic acid (eluent B). The LC gradient was as follows: hold 55% B for 1 min, 1 min linear gradient from 55% B to 65% B, 6 min linear gradient from 65% B to 89% B, 1 min linear gradient from 89% B to 99% B, hold 99% B for 3 min, 0.3 min linear gradient from 99% B to 55% B, hold 55% B for 0.7 min with a flow rate of 0.4 mL/min, autosampler temperature of 10 °C, column temperature of 45 °C and injection volume of 3 µL.

Mass spectrometry was performed using a Synapt G2-S HDMS mass spectrometer (Waters, Manchester, UK) in high-resolution mode and electrospray ionization (ESI) with a scan time for the MS^E^ method (centroid) of 0.1 s. The instrument was operated in positive and negative ion mode, applying the following source parameters: capillary voltage +2.5 kV (ESI^+^), –3.0 kV (ESI^–^), sampling cone 20 V, source offset 40 V, source temperature 120 °C, desolvation temperature 450 °C, cone gas flow 2 L/h, nebulizer 6.5 bar and desolvation gas 850 L/h, and collision energy ramp 20–40 eV. All data were lock mass corrected on the pentapeptide leucine enkephaline (Tyr-Gly-Gly-Phe-Leu, *m/z* 554.2615 [M-H]^–^) in a solution (1 ng/µL) of acetonitrile/0.1% formic acid 1/1 (v/v). Scan time for the lock mass was set to 0.3 s with an interval of 15 s, and three scans were made on average with a mass window of ±0.3 Da. Calibration of the Synapt G2-S in the range of *m/z* 50 to 1,200 was performed using a solution of sodium formate (5 mmol/L) in 2-propanol/water 9/1 (v/v).

Mass spectrometry data were analyzed using MassLynx software (version 4.1 SCN 901, Waters, Manchester, UK). Principal component analysis (PCA) for untargeted metabolomics and lipidomics was conducted using Progenesis QI software (version 3.0, Waters, Manchester, UK) applying the following peak picking conditions: all runs, limits automatic, sensitivity 3, and retention time limits 0.5–11.5 min. Compounds used for PCA were filtered by means of ANOVA *p* ≤ 0.05 and fold change ≥ 2. The processed data were exported to EZinfo (version 3.0, Waters, Manchester, UK), and the matrix was analyzed by PCA with pareto scaling. Group differences were calculated using orthogonal partial least squares discriminant analysis (OPLS-DA) and visualized in S-plots.

## **Figures and tables**


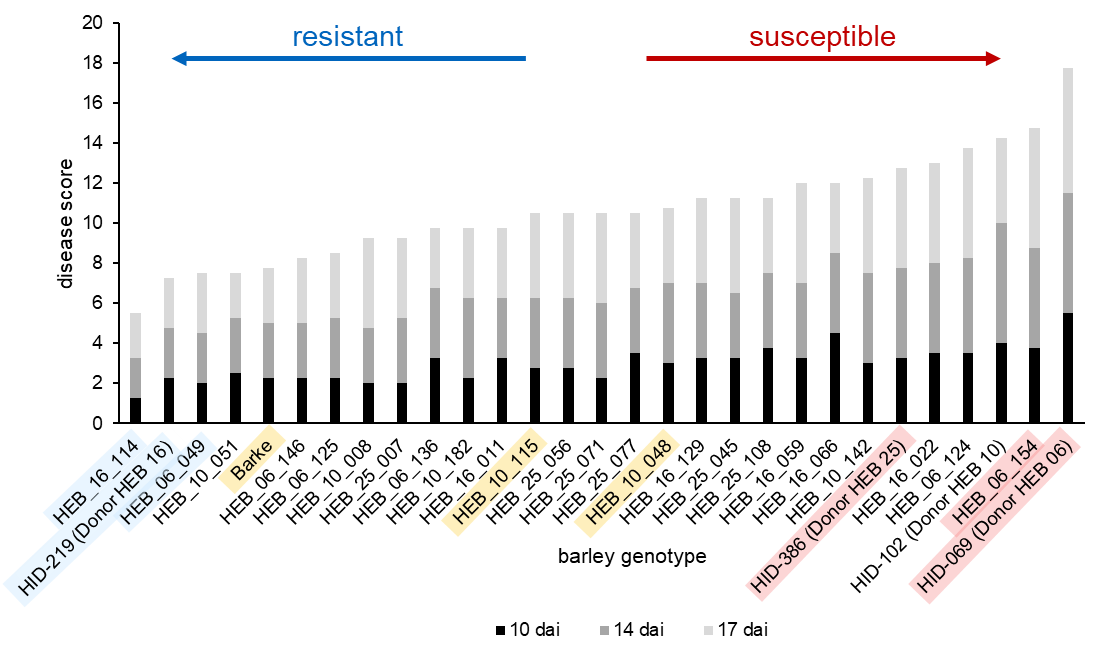


**Figure S1**. Disease score rating of the barley genotypes of the NAM population. Symptoms of spot blotch were evaluated 10, 14 and 17 days after inoculation (dai). Barley lines selected for untargeted metabolomics are highlighted in blue (resistant lines), yellow (medium resistant lines) and red (susceptible lines).

**Table S1.** ESI^+^-TOF-MS² data of triacylglycerols (**17**–**18**) found in barley infected with *B. sorokiniana*. Both substances were verified using reference substances.

| **TG(18:3/18:3/18:3)** (**17**) | | | | **TG(18:2/18:3/18:3)** (**18**) | | | |
| --- | --- | --- | --- | --- | --- | --- | --- |
| Formula | measured *m/z* | calculated *m/z* | deviation (ppm) | Formula | measured *m/z* | calculated *m/z* | deviation (ppm) |
| C_57_H_92_O_6_+NH_4_ | 890.7247 | 890.7232 | 1.68 | C_57_H_93_O_6_+NH_4_ | 892.7391 | 892.7389 | 0.22 |
| C_57_H_92_O_6_+H | 873.7009 | 873.6967 | 4.81 | C_57_H_93_O_6_+H | 875.7128 | 875.7123 | 0.57 |
| C_57_H_91_O_5_ | 855.6870 | 855.6861 | 1.05 | C_57_H_93_O_5_ | 857.6993 | 857.7017 | -2.80 |
| C_39_H_63_O_4_ | 595.4733 | 595.4721 | 2.02 | C_39_H_65_O_4_ | 597.4882 | 597.4877 | 0.84 |
| C_21_H_35_O_3_ | 335.2581 | 335.2581 | 0.00 | C_39_H_63_O_4_ | 595.4708 | 595.4721 | -2.18 |
| C_21_H_33_O_2_ | 317.2474 | 317.2475 | -0.32 | C_21_H_37_O_3_ | 337.2720 | 337.2737 | -5.04 |
| C_18_H_29_O | 261.2213 | 261.2213 | 0.00 | C_21_H_35_O_3_ | 335.2563 | 335.2581 | -5.37 |
| C_18_H_27_ | 243.2105 | 243.2107 | -0.82 | C_21_H_35_O_2_ | 319.2626 | 319.2631 | -1.57 |
|  |  |  |  | C_21_H_33_O_2_ | 317.2471 | 317.2475 | -1.26 |
|  |  |  |  | C_18_H_31_O | 263.2371 | 263.2369 | 0.76 |
|  |  |  |  | C_18_H_29_O | 261.2200 | 261.2213 | -4.98 |
|  |  |  |  | C_18_H_29_ | 245.2257 | 245.2264 | -2.85 |
|  |  |  |  | C_18_H_27_ | 243.2109 | 243.2107 | 0.82 |

**Table S2.** ESI^+^-TOF-MS² data of phosphatidylcholines (**13**–**14**) found in barley infected with B. sorokiniana. The presence of PC(18:3/18:3) was verified using a reference substance.

| **PC(18:3/18:3)** (**13**) | | | | **PC(18:2/18:3)** (**14**) | | | |
| --- | --- | --- | --- | --- | --- | --- | --- |
| Formula | measured *m/z* | calculated *m/z* | deviation (ppm) | Formula | measured *m/z* | calculated *m/z* | deviation (ppm) |
| C_44_H_76_NO_8_P+H | 778.5397 | 778.5381 | 2.06 | C_44_H_78_NO_8_P+H | 780.5545 | 780.5543 | 0.19 |
| C_44_H_76_NO_8_P+Na | 800.5209 | 800.5206 | 0.34 | C_44_H_78_NO_8_P+Na | 802.5357 | 802.5363 | -0.72 |
| C_41_H_67_O_8_P+Na | 741.4473 | 741.4471 | 0.23 | C_41_H_69_O_8_P+Na | 743.4603 | 743.4628 | -3.33 |
| C_39_H_62_O_4_+Na | 617.4545 | 617.4546 | -0.13 | C_39_H_64_O_4_+Na | 619.4711 | 619.4702 | 1.40 |
| C_39_H_63_O_4_ | 595.4731 | 595.4726 | 0.78 | C_39_H_65_O_4_ | 597.4886 | 597.4883 | 0.53 |
| C_5_H_15_NO_4_P | 184.0738 | 184.0739 | -0.39 | C_5_H_15_NO_4_P | 184.0736 | 184.0739 | -1.48 |
| C_2_H_5_O_4_P+Na | 146.9817 | 146.9823 | -4.20 | C_2_H_5_O_4_P+Na | 146.9833 | 146.9823 | 6.68 |
| C_5_H_14_NO | 104.1070 | 104.1075 | -5.18 | C_5_H_14_NO | 104.1074 | 104.1075 | -1.34 |
| C_5_H_12_N | 86.0965 | 86.0970 | -5.51 | C_5_H_12_N | 86.0965 | 86.0970 | -5.51 |

**Table S3.** UPLC^–^-TOF-MS² data of acyl-MGDG (**22**–**24**).

| **18:2-MGDG(18:3/18:3)** (**22**) | | | | | | | |
| --- | --- | --- | --- | --- | --- | --- | --- |
| **ESI^+^** | | | | **ESI^–^** | | | |
| Formula | measured *m/z* | calculated *m/z* | deviation (ppm) | Formula | measured *m/z* | calculated *m/z* | deviation (ppm) |
| C_63_H_104_O_11_+NH_4_ | 1054.7966 | 1054.7922 | 4.14 | C_63_H_104_O_11_+Hac-H | 1095.7723 | 1095.7712 | 1.03 |
| C_63_H_104_O_11_+Na | 1059.7523 | 1059.7476 | 4.40 | C_63_H_104_O_11_-H | 1035.7540 | 1035.7500 | 3.82 |
| C_63_H_104_O_11_+K | 1075.7264 | 1075.7216 | 4.49 | C_45_H_75_O_10_ | 775.5350 | 775.5360 | -1.32 |
| C_39_H_65_O_5_ | 613.4837 | 613.4832 | 0.82 | C_45_H_73_O_10_ | 773.5215 | 773.5204 | 1.45 |
| C_39_H_63_O_4_ | 595.4746 | 595.4726 | 3.30 | C_27_H_47_O_9_ | 515.3230 | 515.3220 | 1.92 |
| C_24_H_41_O_6_ | 425.2901 | 425.2903 | -0.51 | C_27_H_45_O_9_ | 513.3055 | 513.3064 | -1.68 |
| C_21_H_35_O_3_ | 335.2585 | 335.2581 | 1.19 | C_18_H_31_O_2_ | 279.2316 | 279.2324 | -2.88 |
| C_18_H_29_O | 261.2217 | 261.2218 | -0.54 | C_18_H_29_O_2_ | 277.2155 | 277.2168 | -4.69 |

| **18:3-MGDG(18:3/18:3)** (**23**) | | | | | | | |
| --- | --- | --- | --- | --- | --- | --- | --- |
| **ESI^+^** | | | | **ESI^–^** | | | |
| Formula | measured *m/z* | calculated *m/z* | deviation (ppm) | Formula | measured *m/z* | calculated *m/z* | deviation (ppm) |
| C_63_H_102_O_11_+NH_4_ | 1052.7782 | 1052.7766 | 1.53 | C_63_H_102_O_11_+Hac-H | 1093.7561 | 1093.7570 | -0.82 |
| C_63_H_102_O_11_+Na | 1057.7402 | 1057.7320 | 7.77 | C_63_H_102_O_11_-H | 1033.7362 | 1033.7344 | 1.75 |
| C_63_H_102_O_11_+K | 1073.7133 | 1073.7059 | 6.87 | C_45_H_73_O_10_ | 773.5215 | 773.5204 | 1.45 |
| C_39_H_65_O_5_ | 613.4852 | 613.4832 | 3.26 | C_27_H_45_O_9_ | 513.3055 | 513.3064 | -1.68 |
| C_39_H_63_O_4_ | 595.4708 | 595.4726 | -3.08 | C_18_H_29_O_2_ | 277.2173 | 277.2168 | 1.80 |
| C_24_H_39_O_6_ | 423.2747 | 423.2747 | 0.08 |  |  |  |  |
| C_21_H_35_O_3_ | 335.2603 | 335.2581 | 6.56 |  |  |  |  |
| C_18_H_29_O | 261.2200 | 261.2218 | -7.04 |  |  |  |  |
| C_18_H_27_ | 243.2109 | 243.2107 | 0.82 |  |  |  |  |

| **16:0-MGDG(18:3/18:3)** (**24**) | | | | | | | |
| --- | --- | --- | --- | --- | --- | --- | --- |
| **ESI^+^** | | | | **ESI^–^** | | | |
| Formula | measured *m/z* | calculated *m/z* | deviation (ppm) | Formula | measured *m/z* | calculated *m/z* | deviation (ppm) |
| C_61_H_104_O_11_+NH_4_ | 1030.7959 | 1030.7922 | 3.55 | C_61_H_104_O_11_+Hac-H | 1071.7760 | 1071.7712 | 4.51 |
| C_61_H_104_O_11_+Na | 1035.7511 | 1035.7476 | 3.35 | C_61_H_104_O_11_-H | 1011.7524 | 1011.7500 | 2.33 |
| C_61_H_104_O_11_+K | 1051.7231 | 1051.7216 | 1.45 | C_45_H_73_O_10_ | 773.5215 | 773.5204 | 1.45 |
| C_39_H_65_O_5_ | 613.4833 | 613.4832 | 0.16 | C_43_H_75_O_10_ | 751.5397 | 751.5360 | 4.89 |
| C_39_H_63_O_4_ | 595.4708 | 595.4726 | -3.08 | C_27_H_45_O_9_ | 513.3055 | 513.3064 | -1.68 |
| C_22_H_41_O_6_ | 401.2904 | 401.2903 | 0.21 | C_25_H_47_O_9_ | 491.3209 | 491.3220 | -2.26 |
| C_21_H_35_O_3_ | 335.2587 | 335.2581 | 1.79 | C_18_H_29_O_2_ | 277.2155 | 277.2168 | -4.69 |
| C_18_H_29_O | 261.2200 | 261.2218 | -7.04 | C_16_H_31_O_2_ | 255.2324 | 255.2324 | -0.02 |
| C_16_H_31_O | 239.2365 | 239.2375 | -4.14 |  |  |  |  |

**Table S4.** ESI^+^-TOF-MS² data of pheophytine a derivatives (**1**–**3**).

| **Hydroxypheophytin *a*** (**1**) | | | | **Divinylpheophytin *a*** (**2**) | | | | | | | |
| --- | --- | --- | --- | --- | --- | --- | --- | --- | --- | --- | --- |
| Formula | measured  *m/z* | calculated  *m/z* | deviation (ppm) | Formula | | measured  *m/z* | | calculated  *m/z* | | | deviation (ppm) |
| C_55_H_74_N_4_O_6_+H | 887.5691 | 887.5687 | 0.45 | C_55_H_72_N_4_O_5_+H | | 869.5592 | | 869.5581 | | | 1.27 |
| C_55_H_74_N_4_O_6_+Na | 909.5517 | 909.5506 | 1.21 | C_55_H_72_N_4_O_5_+Na | | 891.5395 | | 891.5400 | | | -0.56 |
| C_55_H_74_N_4_O_6_+K | 925.5237 | 925.5245 | -0.86 | C_35_H_35_N_4_O_5_ | | 591.2601 | | 591.2607 | | | -1.01 |
| C_55_H_73_N_4_O_5_ | 869.5572 | 869.5581 | -1.04 | C_34_H_31_N_4_O_4_ | | 559.2338 | | 559.2345 | | | -1.25 |
| C_35_H_37_N_4_O_6_ | 609.2706 | 609.2713 | -1.15 | C_33_H_31_N_4_O_3_ | | 531.2375 | | 531.2396 | | | -3.95 |
| C_35_H_35_N_4_O_5_ | 591.2601 | 591.2607 | -1.01 |  | |  |  | | |  | |
| C_34_H_33_N_4_O_5_ | 577.2426 | 577.2451 | -4.33 |  | |  |  | | |  | |
| C_33_H_33_N_4_O_4_ | 549.2481 | 549.2502 | -3.82 |  | |  |  | | |  | |
| C_33_H_31_N_4_O_3_ | 531.2396 | 531.2396 | 0.00 |  | |  |  | | |  | |
| **Hydroxydivinylpheophytin *a*** (**3**) | | | |  |  | | | |  | | |
| Formula | measured  *m/z* | calculated  *m/z* | deviation (ppm) |  | |  |  | | |  | |
| C_55_H_72_N_4_O_6_+H | 885.5504 | 885.553 | -2.94 |  | |  |  | | |  | |
| C_35_H_35_N_4_O_6_ | 607.2549 | 607.2557 | -1.32 |  | |  |  | | |  | |
| C_35_H_33_N_4_O_5_ | 589.2426 | 589.2451 | -4.24 |  | |  |  | | |  | |
| C_34_H_31_N_4_O_5_ | 575.2272 | 575.2294 | -3.82 |  | |  |  | | |  | |
| C_33_H_31_N_4_O_4_ | 547.2372 | 547.2345 | 4.93 |  | |  |  | | |  | |


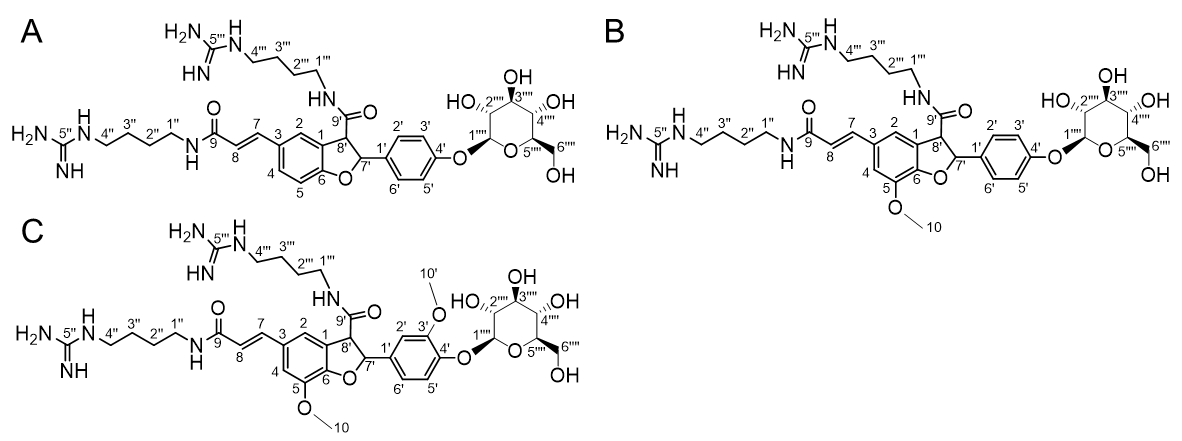


**Figure S2**.Structures of (A) hordatine A glucoside (**28**), (B) hordatine B glucoside (**29**), and (C) hordatine C glucoside (**30**).

**Table S5.** ^1^H-/^13^C-NMR data (500/126 MHz, D_2_O, 300 K) of hordatine glucosides (**28**–**30**) isolated from barley grains.
δ_C_ chemical shift in the ^13^C spectrum, δ_H_ chemical shift in the ^1^H spectrum, M multiplicity of the ^1^H-NMR signals, J coupling constant, s singlet, d doublet, pt pseudo triplet, m multiplet.

|  | **Hordatine A glucoside** (**28**) | | | **Hordatine B glucoside** (**29**) | | | **Hordatine C glucoside** (**30**) | | |
| --- | --- | --- | --- | --- | --- | --- | --- | --- | --- |
|  | *δ*_C_ (ppm) | *δ*_H_ (ppm) | M, *J* (Hz) | *δ*_C_ (ppm) | *δ*_H_ (ppm) | M, *J* (Hz) | *δ*_C_ (ppm) | *δ*_H_ (ppm) | M, *J* (Hz) |
| C1 | 129.3 | - | - | 128.7 | - | - | 129.7 | - | - |
| C2 | 124.3 | 7.17 | s | 116.6 | 7.15 | s | 116.5 | 7.1 | s |
| C3 | 126.5 | - | - | 128.6 | - | - | 129.3 | - | - |
| C4 | 130.9 | 7.48 | d, 8.3 | 113.1 | 7.22 | s | 114.3 | 7.24 | s |
| C5 | 110.3 | 6.87 | d, 8.5 | 147.5 | - | - | 145.4 | - | - |
| C6 | 160.4 | - | - | 149.2 | - | - | 150.3 | - | - |
| C7_cis_ | 136.5 | 6.76 | d, 12.2 | 140.6 | 6.75 | d, 12.2 | 136.8 | 6.71 | d, 12.3 |
| C8_cis_ | 122.5 | 5.92 | d, 12.5 | 123.0 | 5.97 | d, 12.4 | 122.0 | 5.95 | d, 12.0 |
| C7_trans_ | 136.5 | 7.37 | d, 15.8 | 140.6 | 7.34 | d, 15.9 | 140.1 | 7.47 | d, 15.7 |
| C8_trans_ | 118.0 | 6.39 | d, 15.5 | 118.5 | 6.40 | d, 15.7 | 118.3 | 6.56 | d, 15.9 |
| C9 | 168.5 | - | - | 168.5 | - | - | 163.3 | - | - |
| C10 | - | - | - | 56.6 | 3.79 | s | 60.9 | 3.89 | s |
| C1‘ | 133.7 | - | - | 133.7 | - | - | 129.3 | - | - |
| C2‘ | 127.8 | 7.30 | d, 8.8 | 127.7 | 7.30 | d, 8.7 | 109.8 | 7.03 | s |
| C3‘ | 116.8 | 7.07 | dd, 9.0, 2.5 | 116.8 | 7.07 | dd, 9.0, 2.5 | 149.5 | - | - |
| C4‘ | 157.0 | - | - | 156.9 | 5.85 | t, 7.0 | 147.0 | - | - |
| C5‘ | 116.8 | 7.07 | dd, 9.0, 2.5 | 116.8 | 7.07 | dd, 9.0, 2.5 | 118.2 | 6.92 | d, 8.5 |
| C6‘ | 127.8 | 7.30 | d, 8.8 | 127.7 | 7.30 | d, 8.7 | 116.7 | 7.18 | d, 8.5 |
| C7‘ | 87.9 | 5.81 | d, 7.0 | 88.1 | 5.90 | d, 6.0 | 87.8 | 5.97 | d, 8.1 |
| C8‘ | 56.8 | 4.20 | d, 7.3 | 62.0 | 4.22 | d, 7.2 | 57.4 | 4.20 | d, 8.1 |
| C9‘ | 173.2 | - | - | 173.0 | - | - | 171.6 | - | - |
| C10‘ | - | - | - | - | - | - | 60.9 | 3.85 | s |
| C1‘‘, C1‘‘‘ | 39.2, 40.7 | 2.89 | t, 6.6 | 38.9, 39.3 | 2.89 | t, 6.6 | 39.0 | 2.92 | t, 7.8 |
| C2‘‘, C2‘‘‘ | 28.6 | 1.54 | m | 28.6 | 1.54 | m | 26.3 | 1.65 | m |
| C3‘‘, C3‘‘‘ | 18.9 | 1.29 | m | 18.9 | 1.29 | m | 19.5 | 1.43 | m |
| C4‘‘, C4‘‘‘ | 12.8 | 0.91 | t, 7.4 | 12.8 | 0.91 | t, 7.4 | 12.5 | 0.99 | t, 7.0 |
| C5‘‘, C5‘‘‘ | 156.3 | - | - | 156.3 | - | - | 158.4 | - | - |
| C1‘‘‘‘ | 99.9 | 5.05 | dd, 7.3, 2.6 | 100.0 | 5.05 | dd, 7.3, 2.6 | 101.0 | 5.00 | d, 7.0 |
| C2‘‘‘‘ | 72.9 | 3.50-3.58 | m | 72.9 | 3.50-3.58 | m | 73.7 | 3.51 | m |
| C3‘‘‘‘ | 75.6 | 3.50-3.58 | m | 75.6 | 3.50-3.58 | m | 76.5 | 3.48-3.59 | m |
| C4‘‘‘‘ | 69.3 | 3.41 | pt, 9.4 | 69.3 | 3.41 | pt, 9.4 | 70.0 | 3.46 | pt, 5.5 |
| C5‘‘‘‘ | 76.2 | 3.50-3.58 | m | 76.2 | 3.50-3.58 | m | 76.8 | 3.48-3.59 | m |
| C6*α*‘‘‘‘ | 60.5 | 3.67 | m | 60.5 | 3.67 | m | 61.1 | 3.69 | m |
| C6*β*‘‘‘‘ | 60.5 | 3.83 | m | 60.5 | 3.83 | m | 61.1 | 3.89 | m |

**Table S6.** ESI^+^-TOF-MS² data of hordatine glucosides (**28**–**30**) isolated from barley grains.

| **Hordatine A glucoside** (**28**) | | | | **Hordatine B glucoside** (**29**) | | | | **Hordatine C glucoside** (**30**) | | | |
| --- | --- | --- | --- | --- | --- | --- | --- | --- | --- | --- | --- |
| Formula | measured *m/z* | calculated  *m/z* | deviation (ppm) | Formula | measured *m/z* | calculated  *m/z* | deviation (ppm) | Formula | measured *m/z* | calculated  *m/z* | deviation (ppm) |
| C_34_H_48_N_8_O_9_+H | 713.3618 | 713.3617 | 0.14 | C_35_H_50_N_8_O_10_+H | 743.3724 | 743.3723 | 0.13 | C_36_H_52_N_8_O_11_+H | 773.3824 | 773.3828 | -0.52 |
| C_34_H_48_N_8_O_9_+2H | 357.1849 | 357.1845 | 1.12 | C_35_H_50_N_8_O_10_+2H | 372.1894 | 372.1898 | -0.94 | C_36_H_52_N_8_O_11_+2H | 387.1946 | 387.1951 | -1.16 |
| C_34_H_46_N_7_O_9_ | 696.3359 | 696.3352 | 1.01 | C_35_H_48_N_7_O_10_ | 726.3455 | 726.3458 | -0.41 | C_36_H_50_N_7_O_11_ | 756.3550 | 756.3557 | -0.93 |
| C_33_H_47_N_6_O_9_ | 671.3407 | 671.3405 | 0.30 | C_34_H_49_N_6_O_10_ | 701.3500 | 701.3505 | -0.71 | C_35_H_51_N_6_O_11_ | 731.3574 | 731.3616 | -5.74 |
| C_29_H_35_N_4_O_9_ | 583.2422 | 583.2404 | 3.09 | C_30_H_38_N_4_O_10_ | 614.2593 | 614.2582 | 1.79 | C_31_H_40_N_4_O_11_ | 644.2672 | 644.2688 | -2.48 |
| C_28_H_39_N_8_O_4_ | 551.3097 | 551.3089 | 1.45 | C_29_H_41_N_8_O_5_ | 581.3203 | 581.3200 | 0.52 | C_30_H_43_N_8_O_6_ | 611.3314 | 611.3306 | 1.31 |
| C_28_H_36_N_7_O_4_ | 534.2803 | 534.2829 | -4.87 | C_29_H_38_N_7_O_5_ | 564.2938 | 564.2934 | 0.71 | C_30_H_40_N_7_O_6_ | 594.3051 | 594.3035 | 2.69 |
| C_23_H_25_N_4_O_4_ | 421.1848 | 421.1871 | -5.46 | C_24_H_27_N_4_O_5_ | 451.1975 | 451.1981 | -1.33 | C_25_H_29_N_4_O_6_ | 481.2096 | 481.2082 | 2.91 |
| C_22_H_27_N_4_O_3_ | 395.2073 | 395.2083 | -2.53 | C_23_H_29_N_4_O_4_ | 425.2180 | 425.2189 | -2.12 | C_24_H_31_N_4_O_5_ | 455.2300 | 455.2294 | 1.32 |
| C_18_H_11_O_4_ | 291.0653 | 291.0652 | 0.34 | C_19_H_13_O_5_ | 321.0754 | 321.0757 | -0.93 | C_20_H_15_O_6_ | 351.0860 | 351.0869 | -2.56 |
| C_17_H_13_O_3_ | 265.0861 | 265.0859 | 0.75 | C_18_H_15_O_4_ | 295.0960 | 295.0965 | -1.69 | C_19_H_17_O_5_ | 325.1065 | 325.1076 | -3.38 |
| C_17_H_11_O_3_ | 263.0703 | 263.0703 | 0.00 | C_18_H_13_O_4_ | 293.0804 | 293.0808 | -1.36 | C_19_H_15_O_5_ | 323.0905 | 323.0919 | -4.33 |
| C_17_H_11_O_2_ | 247.0752 | 247.0759 | -2.83 | C_18_H_13_O_3_ | 277.0854 | 277.0865 | -3.97 | C_19_H_15_O_4_ | 307.0961 | 307.0970 | -2.93 |
| C_16_H_11_O_2_ | 235.0751 | 235.0759 | -3.40 | C_16_H_11_O_2_ | 235.0748 | 235.0759 | -4.68 | C_16_H_11_O_2_ | 235.0757 | 235.0759 | -0.85 |
| C_6_H_13_N_4_O | 157.1085 | 157.1089 | -2.55 | C_6_H_13_N_4_O | 157.1081 | 157.1089 | -5.09 | C_6_H_13_N_4_O | 157.1086 | 157.1089 | -1.91 |
| C_5_H_15_N_4_ | 131.1292 | 131.1295 | -2.29 | C_5_H_15_N_4_ | 131.1287 | 131.1295 | -6.10 | C_5_H_15_N_4_ | 131.1292 | 131.1295 | -2.29 |
| C_5_H_12_N_3_ | 114.1024 | 114.1031 | -6.13 | C_5_H_12_N_3_ | 114.1025 | 114.1031 | -5.26 | C_5_H_12_N_3_ | 114.1027 | 114.1031 | -3.51 |
| C_4_H_10_N | 72.0810 | 72.0813 | -4.44 | C_4_H_10_N | 72.0811 | 72.0808 | 4.16 | C_4_H_10_N | 72.0810 | 72.0813 | -4.44 |

**Table S7.** ESI^+^-TOF-MS² data of hordatine A, B and C (**25**–**27**) in healthy barley plants.

| **Hordatine A** (**25**) | | | | **Hordatine B** (**26**) | | | | | **Hordatine C** (**27**) | | | |
| --- | --- | --- | --- | --- | --- | --- | --- | --- | --- | --- | --- | --- |
| Formula | measured *m/z* | calculated *m/z* | deviation (ppm) | Formula | measured *m/z* | calculated *m/z* | | deviation (ppm) | Formula | measured *m/z* | calculated *m/z* | deviation (ppm) |
| C_28_H_38_N_8_O_4_+H | 551.3112 | 551.3094 | 3.26 | C_29_H_40_N_8_O_5_+H | 581.3195 | 581.3200 | | -0.86 | C_30_H_42_N_8_O_6_+H | 611.3308 | 611.3306 | 0.33 |
| C_28_H_38_N_8_O_4_+2H | 276.1599 | 276.1587 | 4.53 | C_29_H_40_N_8_O_5_+2H | 291.1639 | | 291.1639 | 0.00 | C_30_H_42_N_8_O_6_+2H | 306.1689 | 306.1692 | -0.98 |
| C_28_H_36_N_7_O_4_ | 534.2844 | 534.2829 | 2.81 | C_29_H_38_N_7_O_5_ | 564.2957 | | 564.2934 | 4.08 | C_30_H_40_N_7_O_6_ | 594.3060 | 594.3040 | 3.37 |
| C_27_H_37_N_6_O_4_ | 509.2890 | 509.2876 | 2.75 | C_28_H_39_N_6_O_5_ | 539.2993 | | 539.2982 | 2.04 | C_29_H_41_N_6_O_6_ | 569.3100 | 569.3088 | 2.11 |
| C_23_H_25_N_4_O_4_ | 421.1891 | 421.1876 | 3.56 | C_24_H_27_N_4_O_5_ | 451.1994 | | 451.1981 | 2.88 | C_25_H_29_N_4_O_6_ | 481.2074 | 481.2087 | -2.70 |
| C_18_H_11_O_4_ | 291.0667 | 291.0657 | 3.44 | C_19_H_13_O_5_ | 321.0770 | | 321.0763 | 2.18 | C_20_H_15_O_6_ | 351.0868 | 351.0869 | -0.28 |
| C_17_H_13_O_3_ | 265.0876 | 265.0865 | 4.15 | C_18_H_15_O_3_ | 295.0958 | | 295.0965 | -2.37 | C_19_H_17_O_5_ | 325.1075 | 325.1076 | -0.31 |
| C_17_H_11_O_3_ | 263.0705 | 263.0708 | -1.14 | C_18_H_13_O_4_ | 293.0813 | | 293.0814 | -0.34 | C_17_H_9_O_5_ | 293.0444 | 293.045 | -2.05 |
| C_17_H_11_O_2_ | 247.0766 | 247.0759 | 2.83 | C_17_H_10_O_3_ | 262.0613 | | 262.0624 | -4.20 | C_16_H_9_O_4_ | 265.0498 | 265.0501 | -1.13 |
| C_16_H_13_O_2_ | 237.0916 | 237.0916 | 0.00 | C_16_H_10_O_3_ | 250.0625 | | 250.0630 | -2.00 | C_16_H_10_O_3_ | 250.0629 | 250.0630 | -0.40 |
| C_16_H_11_O_2_ | 235.0767 | 235.0759 | 3.40 | C_16_H_11_O_2_ | 235.0770 | | 235.0759 | 4.68 | C_16_H_13_O_2_ | 237.0910 | 237.0916 | -2.53 |
| C_16_H_11_O | 219.0818 | 219.0810 | 3.65 | C_15_H_10_O_2_ | 222.0677 | | 222.0681 | -1.80 | C_16_H_11_O_2_ | 235.0748 | 235.0759 | -4.68 |
| C_14_H_10_ | 178.0780 | 178.0783 | -1.68 | C_14_H_10_ | 178.0780 | | 178.0783 | -1.68 | C_15_H_10_O_2_ | 222.0670 | 222.0681 | -4.95 |
| C_6_H_13_N_4_O | 157.1082 | 157.1089 | -4.46 | C_6_H_13_N_4_O | 157.1082 | | 157.1089 | -4.46 | C_6_H_13_N_4_O | 157.1084 | 157.1089 | -3.18 |
| C_5_H_15_N_4_ | 131.1287 | 131.1291 | -3.05 | C_5_H_15_N_4_ | 131.1287 | | 131.1291 | -3.05 | C_5_H_15_N_4_ | 131.1295 | 131.1291 | 3.05 |
| C_5_H_12_N_3_ | 114.1034 | 114.1031 | 2.63 | C_5_H_12_N_3_ | 114.1034 | | 114.1031 | 2.63 | C_5_H_12_N_3_ | 114.1026 | 114.1031 | -4.38 |
| C_4_H_10_N | 72.0814 | 72.0813 | 1.11 | C_4_H_10_N | 72.0814 | | 72.0813 | 1.11 | C_4_H_10_N | 72.0812 | 72.0813 | -1.66 |

**Table S8.** ESI^+^-TOF-MS² data of p-CA (**31**), p-CHA (**32**), and p-CHDA (**33**).

| ***p*-CA** (**31**) | | | | ***p*-CHA** (**32**) | | | | ***p*-CHDA** (**33**) | | | |
| --- | --- | --- | --- | --- | --- | --- | --- | --- | --- | --- | --- |
| Formula | measured *m/z* | calculated  *m/z* | deviation (ppm) | Formula | measured *m/z* | calculated  *m/z* | deviation (ppm) | Formula | measured *m/z* | calculated  *m/z* | deviation (ppm) |
| C_14_H_20_N_4_O_2_+H | 277.1664 | 277.1665 | -0.36 | C_14_H_20_N_4_O_3_+H | 293.1612 | 293.1614 | -0.68 | C_14_H_18_N_4_O_3_+H | 291.1458 | 291.1457 | 0.34 |
| C_14_H_18_N_3_O_2_ | 260.1404 | 260.1399 | 1.92 | C_14_H_19_N_4_O_2_ | 275.1508 | 275.1508 | 0.00 | C_14_H_16_N_4_O_2_ | 273.1350 | 273.1352 | -0.73 |
| C_13_H_16_NO_2_ | 218.1190 | 218.1181 | 4.13 | C_13_H_17_N_2_O_2_ | 233.1279 | 233.1290 | -4.72 | C_14_H_15_N_4_O | 255.1246 | 255.1246 | 0.00 |
| C_9_H_7_O_2_ | 147.0442 | 147.0446 | -2.72 | C_13_H_15_NO_2_ | 216.1033 | 216.1025 | 3.70 | C_13_H_11_NO_2_ | 147.0442 | 147.0446 | -2.72 |
| C_8_H_7_O | 119.0496 | 119.0497 | -0.84 | C_9_H_7_O_2_ | 147.0442 | 147.0446 | -2.72 | C_5_H_11_N_4_ | 127.0987 | 127.0984 | 2.36 |
| C_5_H_12_N_3_ | 114.1034 | 114.1031 | 2.63 | C_5_H_12_N_3_O | 130.0975 | 130.0980 | -3.84 | C_8_H_7_O | 119.0497 | 119.0497 | 0.00 |
| C_7_H_7_ | 91.0547 | 91.0548 | -0.88 | C_5_H_13_N_4_ | 129.1145 | 129.1140 | 3.87 | C_5_H_8_N_3_ | 110.0715 | 110.0718 | -2.73 |
| C_4_H_10_N | 72.0816 | 72.0813 | 3.88 | C_8_H_7_O | 119.0497 | 119.0497 | 0.00 | C_7_H_7_ | 91.0548 | 91.0548 | 0.22 |
|  |  |  |  | C_5_H_10_N_3_ | 112.0870 | 112.0875 | -4.46 | C_4_H_9_N_2_ | 85.0762 | 85.0766 | -4.35 |
|  |  |  |  | C_7_H_7_ | 91.0549 | 91.0548 | 1.32 | C_4_H_7_N_2_ | 83.0605 | 83.0604 | 1.20 |
|  |  |  |  | C_4_H_8_N | 70.0659 | 70.0657 | 3.28 | C_4_H_6_N | 68.0502 | 68.0500 | 2.65 |

**
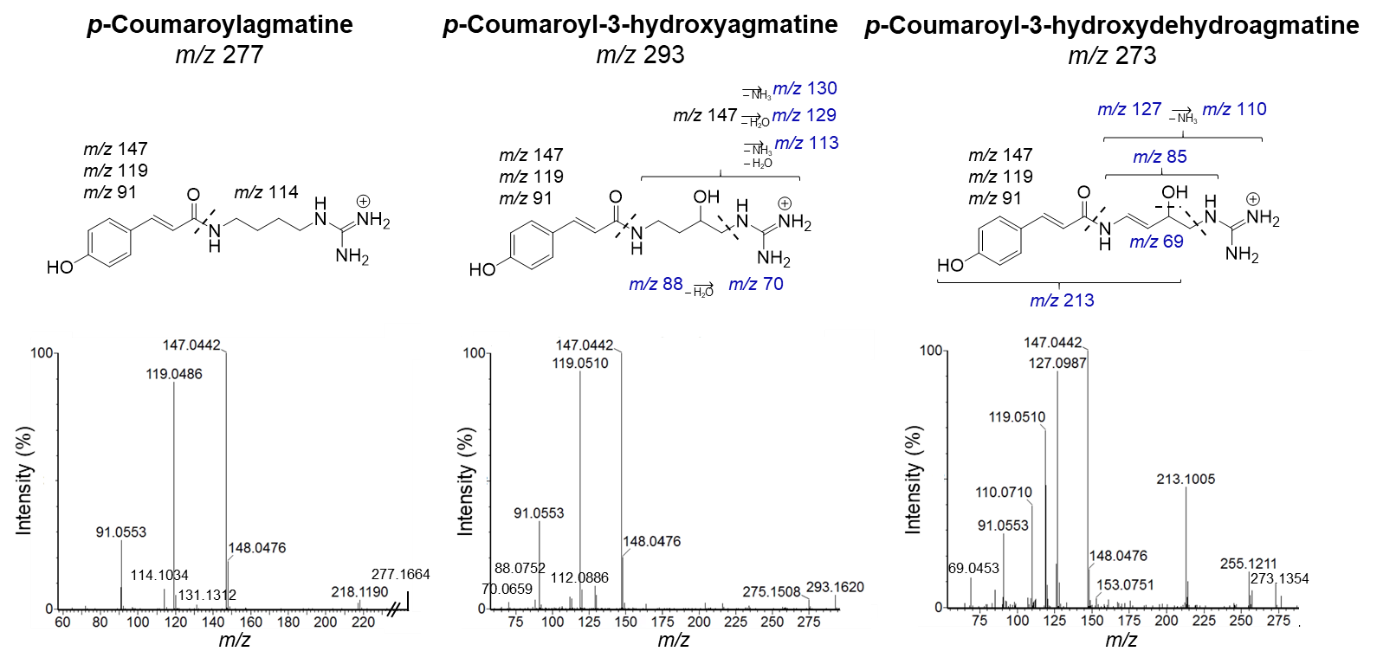
**

**Figure S3.** ESI^+^-TOF-MS² fragmentation patterns of p-coumaroylagmatine derivatives (**31**–**33**) in barley (characteristic m/z values for each compound are marked in blue). In barley leaves two isomers of each compound were detected. This figure shows the later eluting (E)-isomers.


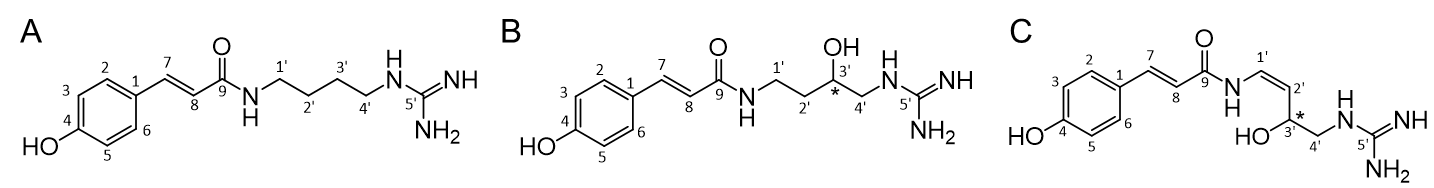


**Figure S4.** Structures of (E)-p-CA (**31**), (E)-p-CHA (**32**), and (7E,1’Z)-p-CHDA (**33**). In barley leaves two isomers of each compound were detected. The later eluting signals were isolated and determined by NMR spectroscopy. * Stereochemistry not defined.

**Table S9.** ^1^H-/^13^C-NMR data (600/151 MHz, methanol-d_4_, 300 K) of (E)-p-CA (**31**), (E)-p-CHA (**32**), and (7E,1’Z)-p-CHDA (**33**). δ_C_ chemical shift in the ^13^C spectrum, δ_H_ chemical shift in the ^1^H spectrum, M multiplicity of the ^1^H-NMR signals, J coupling constant, d doublet, dd double doublet, t triplet, m multiplet.

|  | ***p*-CA** (**31**) | | | ***p*-CHA** (**32**) | | | ***p*-CHDA** (**33**) | | |
| --- | --- | --- | --- | --- | --- | --- | --- | --- | --- |
|  | *δ*_C_ (ppm) | *δ*_H_ (ppm) | M, *J* (Hz) | *δ*_C_ (ppm) | *δ*_H_ (ppm) | M, *J* (Hz) | *δ*_C_ (ppm) | *δ*_H_ (ppm) | M, *J* (Hz) |
| C1 | 129.3 | - | - | 126.5 | - | - | 126.1 | - | - |
| C2, C6 | 129.2 | 7.43 | d, 8.7 | 128.9 | 7.46 | d, 8.9 | 129.8 | 7.45 | dd, 8.9, 2.2 |
| C3, C5 | 115.4 | 6.82 | d, 8.7 | 115.5 | 6.82 | d, 8.8 | 115.3 | 6.82 | dd, 8.8, 2.0 |
| C4 | 157.4 | - | - | 159.1 | - | - | 159.0 | - | - |
| C7 | 140.6 | 7.48 | d, 16.2 | 141.1 | 7.50 | d, 16.4 | 141.8 | 7.55 | d, 16.0 |
| C8 | 116.8 | 6.44 | d, 15.8 | 116.6 | 6.49 | d, 16.2 | 116.1 | 6.52 | d, 16.3 |
| C9 | 168.2 | - | - | 167.8 | - | - | 169.0 | - | - |
| C1’ | 38.3 | 3.35 | t, 6.5 | 38.3 | 3.36 | dd, 14.5, 4.3 | 127.7 | 7.23 | m |
| C2’*α* | 25.8 | 1.65 | m | 33.1 | 1.80 | m | 119.6 | 5.93 | d, 12.5 |
| C2’*β* | - | - | - | 33.1 | 1.64 | m | - | - | - |
| C3’ | 26.4 | 1.65 | m | 68.1 | 3.80 | m | 79.6 | 4.23 | m |
| C4*α*’ | 40.7 | 3.25 | t, 6.5 | 45.3 | 3.29 | dd, 14.0, 6.8 | 47.0 | 3.66 | d, 14.0 |
| C4*β*’ | - | - | - | 45.3 | 3.43 | dd, 14.1, 4.9 | 47.0 | 3.53 | d, 14.0 |
| C5’ | 159.3 | - | - | 169.6 | - | - | 169.1 | - | - |

**
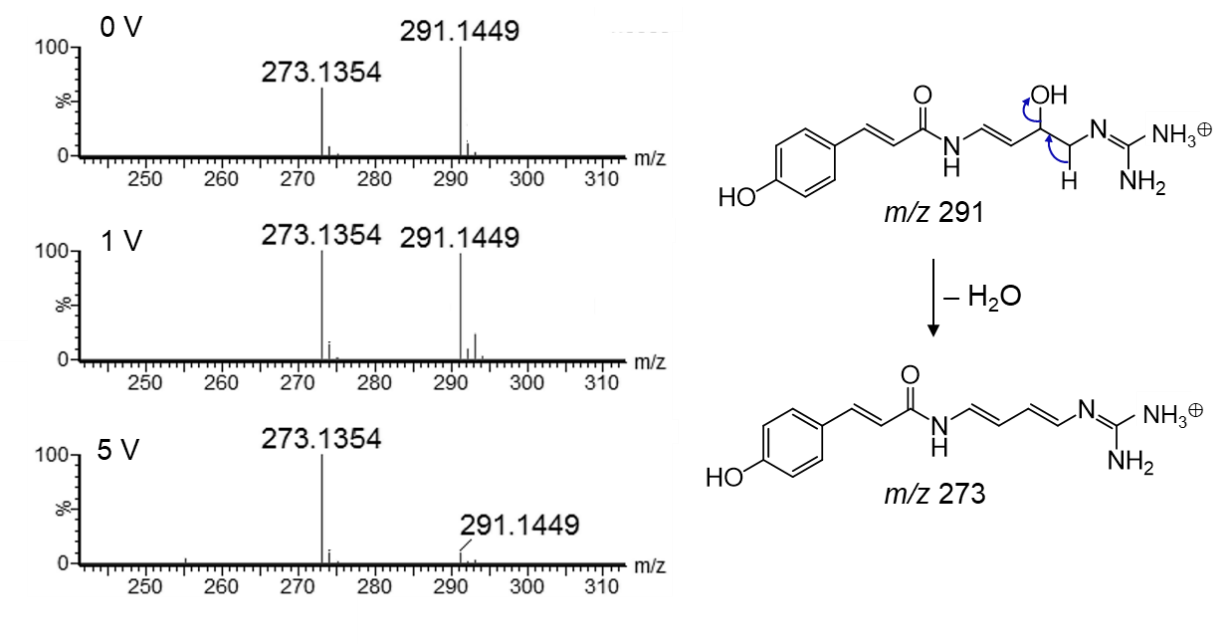
**

**Figure S5.** Relative abundance of the adduct ions of p-CHDA (**33**) measured in ESI^+^-MS² with varying collision energy.


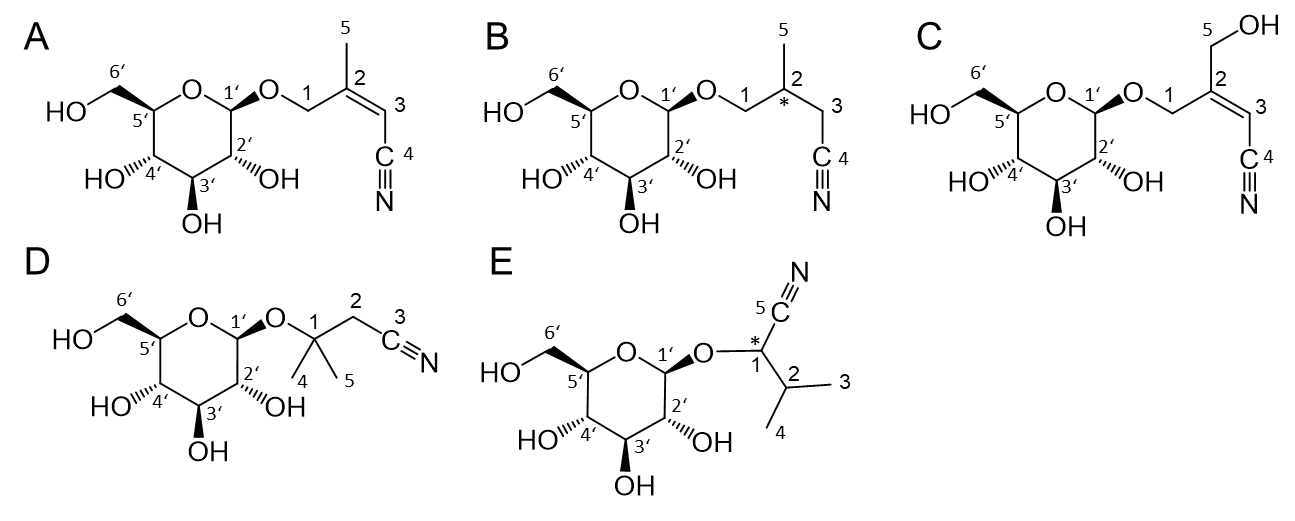


**Figure S6**. Structures of the cyano glucosides (A) osmaronin (**40**), (B) dihydroosmaronin (**41**), (C) sutherlandin (**39**), (D) epidermin (**42**), and (E) epiheteroendrin (**38**). * Stereochemistry not defined.

**Table S10.** ^1^H-/^13^C-NMR data (500/126 MHz, D_2_O, 300 K) of cyano glucosides (**38**–**42**).

δ_C_ chemical shift in the ^13^C spectrum, δ_H_ chemical shift in the ^1^H spectrum, M multiplicity of the ^1^H-NMR signals, J coupling constant, s singlet, d doublet, pt pseudo triplet, m multiplet.

|  | **Osmaronin** (**40**) | | | **Dihydroosmaronin** (**41**) | | | **Sutherlandin** (**39**) | | | **Epidermin** (**42**) | | | **Epiheteroendrin** (**38**) | | |
| --- | --- | --- | --- | --- | --- | --- | --- | --- | --- | --- | --- | --- | --- | --- | --- |
|  | *δ*_C_ (ppm) | *δ*_H_ (ppm) | M, *J* (Hz) | *δ*_C_ (ppm) | *δ*_H_ (ppm) | M, *J* (Hz) | *δ*_C_ (ppm) | *δ*_H_ (ppm) | M, *J* (Hz) | *δ*_C_ (ppm) | *δ*_H_ (ppm) | M, *J* (Hz) | *δ*_C_ (ppm) | *δ*_H_ (ppm) | M, *J* (Hz) |
| C1 | 69.4 | 4.46 | s | 73.4 | 3.83 | m | 66.9 | 4.52 | d, 5.1 | 75.9 | - | - | 74 | 4.47 | d, 5.9 |
| C2 | 161.2 | - | - | 30.3 | 2.15 | o, 6.1 | 162.7 | - | - | 29.9 | 2.74 | dd, 17.2, 5.7 | 31.4 | 2.11 | o, 6.8 |
| C3*α* | 97.4 | 5.45 | s | 20.5 | 2.47 | dd, 16.5, 7.0 | 96.2 | 5.71 | s | 119.3 | - | - | 16.5 | 0.98 | d, 6.8 |
| C3*β* | - | - | - | 20.5 | 2.55 | dd, 16.6, 5.5 | - | - | - | - | - | - | - | - | - |
| C4 | 117.0 | - | - | 120.6 | - | - | 116.8 | - | - | 25.2 | 1.34 | s | 16.9 | 0.98 | d, 6.8 |
| C5 | 20.0 | 1.93 | s | 15.2 | 0.98 | d, 6.9 | 61.4 | 4.27 | s | 25.7 | 1.34 | s | 119.2 | - | - |
| C1‘ | 69.7 | 4.37 | d, 7.9 | 102.7 | 4.36 | d, 8.0 | 101.7 | 4.39 | d, 8.0 | 96.6 | 4.58 | d, 7.9 | 103.4 | 4.51 | d, 8.2 |
| C2‘ | 72.9 | 3.23 | pt, 7.7 | 73.0 | 3.19 | dd, 8.0, 1.1 | 72.9 | 3.22 | t, 8.6 | 73.1 | 3.16 | pt, 7.7 | 72.9 | 3.24 | pt, 7.6 |
| C3‘ | 76.0 | 3.35-3.42 | m | 75.9 | 3.34-3.45 | m | 76.0 | 3.28-3.43 | m | 75.7 | 3.36-3.46 | m | 76.2 | 3.37-3.43 | m |
| C4‘ | 75.7 | 3.35-3.42 | m | 75.6 | 3.34-3.45 | m | 75.6 | 3.28-3.43 | m | 75.6 | 3.36-3.46 | m | 75.5 | 3.37-3.43 | m |
| C5‘ | 69.4 | 3.32 | t, 9.1 | 69.7 | 3.29 | pt, 9.3 | 69.5 | 3.32 | pt, 9.1 | 69.5 | 3.30 | t, 9.2 | 69.4 | 3.33 | pt, 9.2 |
| C6*α*’ | 60.5 | 3.83 | dd, 12.5, 2.2 | 60.6 | 3.83 | m | 60.6 | 3.83 | dd, 12.5, 1.8 | 60.8 | 3.80 | dd, 12.5, 2.0 | 60.4 | 3.85 | dd, 12.4, 1.9 |
| C6*β*‘ | 60.5 | 3.65 | dd, 12.5, 5.5 | 60.6 | 3.63 | dd, 12.3, 5.8 | 60.6 | 3.64 | dd, 12.5, 5.6 | 60.8 | 3.62 | dd, 12.4, 5.5 | 60.4 | 3.66 | dd, 12.5, 5.5 |

**Table S11.** ESI-TOF-MS² data of flavone glucosides (**34**–**37**).

| **Isovitexin 7-*O*-rhamnosylglucoside** (**34**) | | | | | | | |
| --- | --- | --- | --- | --- | --- | --- | --- |
| **ESI^+^** | | | | **ESI^–^** | | | |
| Formula | measured *m/z* | calculated *m/z* | deviation (ppm) | Formula | measured *m/z* | calculated *m/z* | deviation (ppm) |
| C_33_H_40_O_19_+H | 741.2256 | 741.2242 | 1.89 | C_33_H_40_O_19_-H | 739.2093 | 739.2086 | 0.95 |
| C_33_H_40_O_19_+Na | 763.2056 | 763.2061 | -0.66 | C_23_H_21_O_11_ | 473.1065 | 473.1084 | -4.02 |
| C_27_H_31_O_15_ | 595.1669 | 595.1663 | 1.01 | C_22_H_21_O_10_ | 445.1123 | 445.1135 | -2.70 |
| C_27_H_29_O_14_ | 577.1534 | 577.1557 | -3.99 | C_21_H_19_O_10_ | 431.0995 | 431.0978 | 3.94 |
| C_23_H_19_O_10_ | 455.0973 | 455.0978 | -1.10 | C_18_H_13_O_7_ | 341.0667 | 341.0661 | 1.76 |
| C_21_H_21_O_10_ | 433.1131 | 433.1135 | -0.92 | C_17_H_11_O_6_ | 311.0546 | 311.0556 | -3.21 |
| C_21_H_17_O_8_ | 397.0918 | 397.0923 | -1.26 | C_16_H_11_O_5_ | 283.0604 | 283.0606 | -0.71 |
| C_21_H_15_O_7_ | 379.0823 | 379.0818 | 1.32 |  |  |  |  |
| C_20_H_15_O_7_ | 367.0823 | 367.0818 | 1.36 |  |  |  |  |
| C_19_H_13_O_6_ | 337.0716 | 337.0712 | 1.19 |  |  |  |  |
| C_17_H_13_O_6_ | 313.0716 | 313.0712 | 1.28 |  |  |  |  |
| C_16_H_11_O_5_ | 283.0611 | 283.0606 | 1.77 |  |  |  |  |

| **Apigenin 6-*C*-glucoside-8-*C*-arabinoside** (**35**) | | | | | | | |
| --- | --- | --- | --- | --- | --- | --- | --- |
| **ESI^+^** | | | | **ESI^–^** | | | |
| Formula | measured *m/z* | calculated *m/z* | deviation (ppm) | Formula | measured *m/z* | calculated *m/z* | deviation (ppm) |
| C_26_H_28_O_14_+H | 565.1559 | 565.1557 | 0.35 | C_26_H_28_O_14_-H | 563.1413 | 563.1401 | 2.13 |
| C_26_H_28_O_14_+Na | 587.1376 | 587.1377 | -0.17 | C_26_H_25_O_13_ | 545.1310 | 545.1295 | 2.75 |
| C_26_H_27_O_13_ | 547.1452 | 547.1452 | 0.00 | C_24_H_23_O_12_ | 503.1205 | 503.1190 | 2.98 |
| C_22_H_17_O_8_ | 409.0939 | 409.0923 | 3.91 | C_23_H_21_O_11_ | 473.1065 | 473.1084 | -4.02 |
| C_21_H_15_O_7_ | 379.0810 | 379.0818 | -2.11 | C_22_H_19_O_10_ | 443.0994 | 443.0978 | 3.61 |
| C_19_H_15_O_7_ | 355.0833 | 355.0818 | 4.22 | C_20_H_15_O_8_ | 383.0768 | 383.0767 | 0.26 |
| C_19_H_13_O_6_ | 337.0714 | 337.0712 | 0.59 | C_19_H_13_O_7_ | 353.0664 | 353.0661 | 0.85 |
| C_18_H_13_O_6_ | 325.0716 | 325.0712 | 1.23 | C_18_H_13_O_6_ | 325.0716 | 325.0712 | 1.23 |
| C_17_H_11_O_5_ | 295.062 | 295.0606 | 4.74 | C_17_H_13_O_5_ | 297.0752 | 297.0763 | -3.70 |

| **Apigenin 7-*O*-arabinosylglucoside** (**36**) | | | | | | | |
| --- | --- | --- | --- | --- | --- | --- | --- |
| **ESI^+^** | | | | **ESI^–^** | | | |
| Formula | measured *m/z* | calculated *m/z* | deviation (ppm) | Formula | measured *m/z* | calculated *m/z* | deviation (ppm) |
| C_26_H_28_O_14_+H | 565.1560 | 565.1557 | 0.53 | C_26_H_28_O_14_-H | 563.1413 | 563.1401 | 2.13 |
| C_21_H_21_O_10_ | 433.1143 | 433.1135 | 1.85 | C_22_H_19_O_10_ | 443.0984 | 443.0978 | 1.35 |
| C_17_H_13_O_6_ | 313.0703 | 313.0712 | -2.87 | C_21_H_19_O_10_ | 431.0995 | 431.0978 | 3.94 |
| C_15_H_11_O_5_ | 271.0599 | 271.0606 | -2.58 | C_21_H_17_O_9_ | 413.0885 | 413.0873 | 2.90 |
|  |  |  |  | C_17_H_11_O_6_ | 311.0546 | 311.0556 | -3.21 |
|  |  |  |  | C_15_H_9_O_5_ | 269.0450 | 269.0450 | 0.00 |

| **Isovitexin 2''-*O*-feruloylglucoside** (**37**) | | | | | | | |
| --- | --- | --- | --- | --- | --- | --- | --- |
| **ESI^+^** | | | | **ESI^–^** | | | |
| Formula | measured *m/z* | calculated *m/z* | deviation (ppm) | Formula | measured *m/z* | calculated *m/z* | deviation (ppm) |
| C_37_H_38_O_18_+H | 771.2136 | 771.2136 | 0.00 | C_37_H_38_O_18_-H | 769.1977 | 769.1980 | -0.39 |
| C_37_H_38_O_18_+Na | 793.1950 | 793.1956 | -0.76 | C_33_H_29_O_14_ | 649.1545 | 649.1557 | -1.85 |
| C_37_H_37_O_17_ | 753.2032 | 753.2031 | 0.13 | C_27_H_29_O_15_ | 593.1495 | 593.1506 | -1.85 |
| C_21_H_21_O_10_ | 433.1129 | 433.1135 | -1.39 | C_23_H_21_O_11_ | 473.1065 | 473.1084 | -4.02 |
| C_21_H_19_O_9_ | 415.1026 | 415.1029 | -0.72 | C_22_H_21_O_10_ | 445.1123 | 445.1135 | -2.70 |
| C_21_H_17_O_8_ | 397.0923 | 397.0923 | 0.00 | C_21_H_19_O_10_ | 431.0995 | 431.0978 | 3.94 |
| C_21_H_15_O_7_ | 379.0816 | 379.0818 | -0.53 | C_21_H_17_O_9_ | 413.0885 | 413.0873 | 2.90 |
| C_20_H_15_O_7_ | 367.0816 | 367.0818 | -0.54 | C_16_H_19_O_9_ | 355.1029 | 355.1029 | 0.00 |
| C_19_H_13_O_6_ | 337.0710 | 337.0712 | -0.59 | C_18_H_13_O_7_ | 341.0666 | 341.0661 | 1.47 |
| C_17_H_13_O_6_ | 313.0710 | 313.0712 | -0.64 | C_18_H_13_O_6_ | 325.0716 | 325.0712 | 1.23 |
| C_16_H_11_O_5_ | 283.0605 | 283.0606 | -0.35 | C_17_H_11_O_6_ | 311.0546 | 311.0556 | -3.21 |
| C_10_H_9_O_3_ | 177.0551 | 177.0552 | -0.56 | C_16_H_11_O_5_ | 283.0604 | 283.0606 | -0.71 |

| **Isovitexin 2''-*O*-feruloylglucoside** (**37**) | | | | | | | |
| --- | --- | --- | --- | --- | --- | --- | --- |
| **ESI^+^** | | | | **ESI^–^** | | | |
| Formula | measured *m/z* | calculated *m/z* | deviation (ppm) | Formula | measured *m/z* | calculated *m/z* | deviation (ppm) |
| C_37_H_38_O_18_+H | 771.2136 | 771.2136 | 0.00 | C_37_H_38_O_18_-H | 769.1977 | 769.1980 | -0.39 |
| C_37_H_38_O_18_+Na | 793.1950 | 793.1956 | -0.76 | C_33_H_29_O_14_ | 649.1545 | 649.1557 | -1.85 |
| C_37_H_37_O_17_ | 753.2032 | 753.2031 | 0.13 | C_27_H_29_O_15_ | 593.1495 | 593.1506 | -1.85 |
| C_21_H_21_O_10_ | 433.1129 | 433.1135 | -1.39 | C_23_H_21_O_11_ | 473.1065 | 473.1084 | -4.02 |
| C_21_H_19_O_9_ | 415.1026 | 415.1029 | -0.72 | C_22_H_21_O_10_ | 445.1123 | 445.1135 | -2.70 |
| C_21_H_17_O_8_ | 397.0923 | 397.0923 | 0.00 | C_21_H_19_O_10_ | 431.0995 | 431.0978 | 3.94 |
| C_21_H_15_O_7_ | 379.0816 | 379.0818 | -0.53 | C_21_H_17_O_9_ | 413.0885 | 413.0873 | 2.90 |
| C_20_H_15_O_7_ | 367.0816 | 367.0818 | -0.54 | C_16_H_19_O_9_ | 355.1029 | 355.1029 | 0.00 |
| C_19_H_13_O_6_ | 337.0710 | 337.0712 | -0.59 | C_18_H_13_O_7_ | 341.0666 | 341.0661 | 1.47 |
| C_17_H_13_O_6_ | 313.0710 | 313.0712 | -0.64 | C_18_H_13_O_6_ | 325.0716 | 325.0712 | 1.23 |
| C_16_H_11_O_5_ | 283.0605 | 283.0606 | -0.35 | C_17_H_11_O_6_ | 311.0546 | 311.0556 | -3.21 |
| C_10_H_9_O_3_ | 177.0551 | 177.0552 | -0.56 | C_16_H_11_O_5_ | 283.0604 | 283.0606 | -0.71 |

**
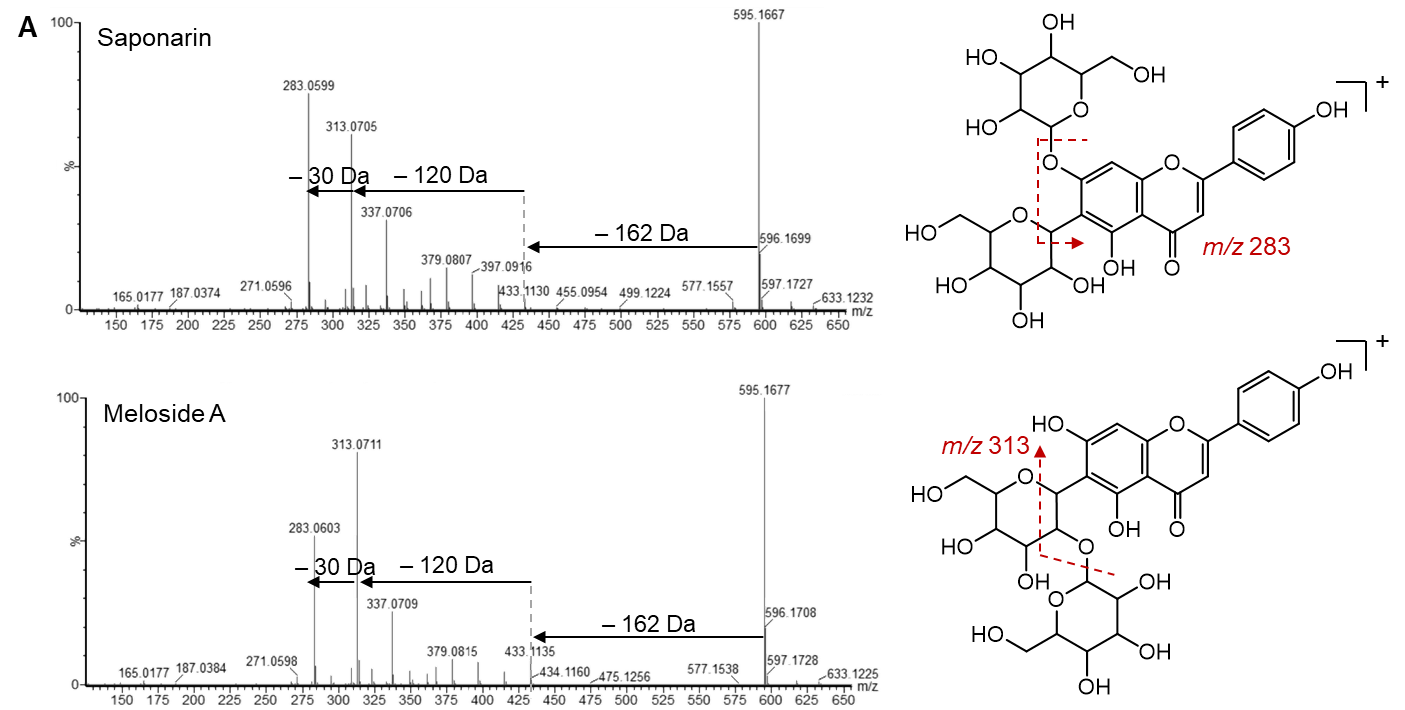
**


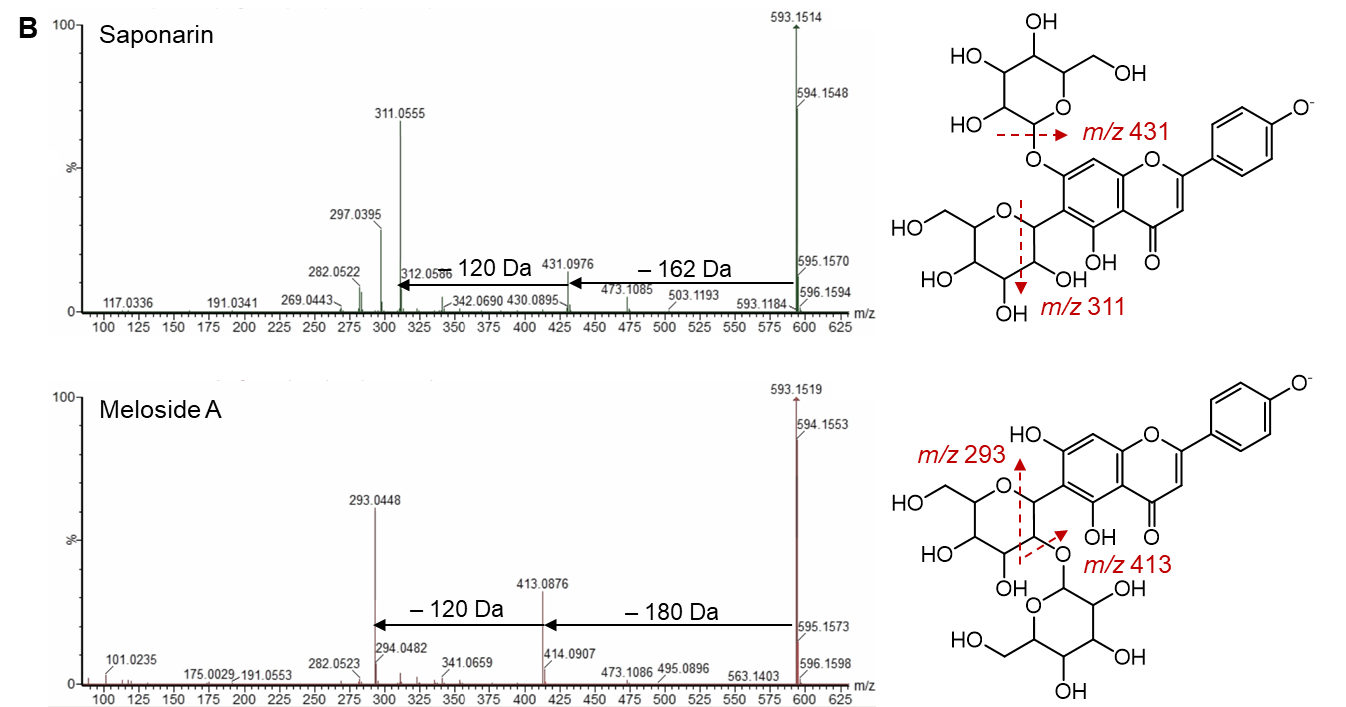


**Figure S7.** MS fragmentation patterns of isomeric flavone glucosides (**56**–**57**) in (A) ESI^+^ and (B) ESI^–^ mode.

**
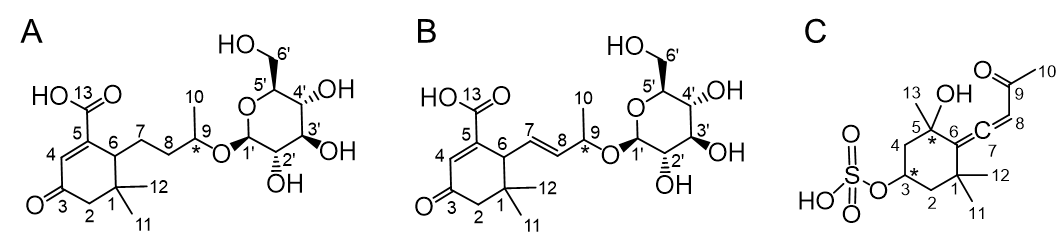
**

**Figure S8.** Structures of (A) 5-carboxyblumenol C 9-O-glucoside (**45**), (B) 5-carboxydidehydroblumenol C 9-O-glucoside (**46**), and (C) grasshopper ketone-3-sulfate (**47**). * Stereochemistry not defined.

**Table S12.** ^1^H-/^13^C-NMR data (500/126 MHz, DMSO-d_6_, 300 K) of 5-carboxyblumenol C 9-O-glucoside (**45**), 5‑carboxydidehydroblumenol C 9-O-glucoside (**46**), and grasshopper ketone-3-sulfate (**47**).

δ_C_ chemical shift in the ^13^C spectrum, δ_H_ chemical shift in the ^1^H spectrum, M multiplicity of the ^1^H-NMR signals, J coupling constant, s singlet, d doublet, dd doublet of doublet, ddd doublet of doublets of doublets, t triplet, q quartet, m multiplet.

|  | **5-carboxyblumenol C**  **9-O-glucoside** (**45**) | | | **5-carboxydidehydroblumenol C**  **9-O-glucoside** (**46**) | | | **grasshopper ketone-3-**  **sulfate** (**47**) | | |
| --- | --- | --- | --- | --- | --- | --- | --- | --- | --- |
|  | *δ*_C_ (ppm) | *δ*_H_ (ppm) | M, *J* (Hz) | *δ*_C_ (ppm) | *δ*_H_ (ppm) | M, *J* (Hz) | *δ*_C_ (ppm) | *δ*_H_ (ppm) | M, *J* (Hz) |
| C1 | 36.2 | - | - | 36.3 | - | - | 36.0 | - | - |
| C2*α* | 46.8 | 1.95 | d, 17.4 | 47.4 | 1.98 | d,16.3 | 46.9 | 2.07 | ddd, 12.3, 4.2, 2.0 |
| C2*β* | 46.8 | 2.57 | d, 17.4 | 47.4 | 2.51 | m | 46.9 | 1.23 | d, 12.0 |
| C3 | 200.0 | - | - | 200.5 | - | - | 69.3 | 4.65 | m |
| C4*α* | 128.2 | 6.33 | s | 128.3 | 6.35 | s | 47.3 | 2.28 | ddd, 12.8, 4.4, 1.9 |
| C4*β* | - | - | - | - | - | - | 47.3 | 1.28 | d, 12.0 |
| C5 | 156.2 | - | - | 154.3 | - | - | 71.0 | - | - |
| OH | - | - | - | - | - | - | - | 5.03 | s |
| C6 | 44.1 | 2.53 | m | 48.8 | 3.20 | d, 8.4 | 119.0 | - | - |
| C7*α* | 26.1 | 1.54 | m | 129.1 | 5.78 | dd, 16.4, 8.8 | 209.6 | - | - |
| C7*β* | 26.1 | 1.79 | m | - | - | - | - | - | - |
| C8 | 34.6 | 1.42 | m | 134.9 | 5.39 | dd, 15.6, 7.1 | 100.1 | 5.75 | s |
| C9 | 75.4 | 3.68 | m | 72.8 | 4.32 | t, 6.5 | 198.4 | - | - |
| C10 | 21.8 | 1.11 | d, 6.3 | 22.3 | 1.13 | d, 6.7 | 26.6 | 2.12 | s |
| C11 | 27.2 | 1.05 | s | 27.7 | 0.98 | s | 32.0 | 1.07 | s |
| C12 | 28.0 | 0.92 | s | 27.6 | 0.96 | s | 29.1 | 1.32 | s |
| C13 | 169.0 | - | - | 168.3 | - | - | 30.7 | 1.27 | s |
| C1’ | 102.9 | 4.13 | d, 7.8 | 100.2 | 4.11 | d, 7.8 | - | - | - |
| C2’ | 73.6 | 2.88 | t, 8.4 | 73.9 | 2.92 | t, 8.2 | - | - | - |
| C3’ | 76.8 | 3.11 | t, 8.8 | 77.3 | 3.11 | t, 8.6 | - | - | - |
| C4’ | 70.1 | 3.01 | m | 70.6 | 3.00 | m | - | - | - |
| C5’ | 76.7 | 3.06 | m | 77.3 | 2.98 | m | - | - | - |
| C6’*α* | 61.2 | 3.41 | q, 5.8 | 61.5 | 3.40 | m | - | - | - |
| C6’*β* | 61.2 | 3.65 | dd, 11.7, 1.9 | 61.5 | 3.64 | dd,11.8, 1.7 | - | - | - |


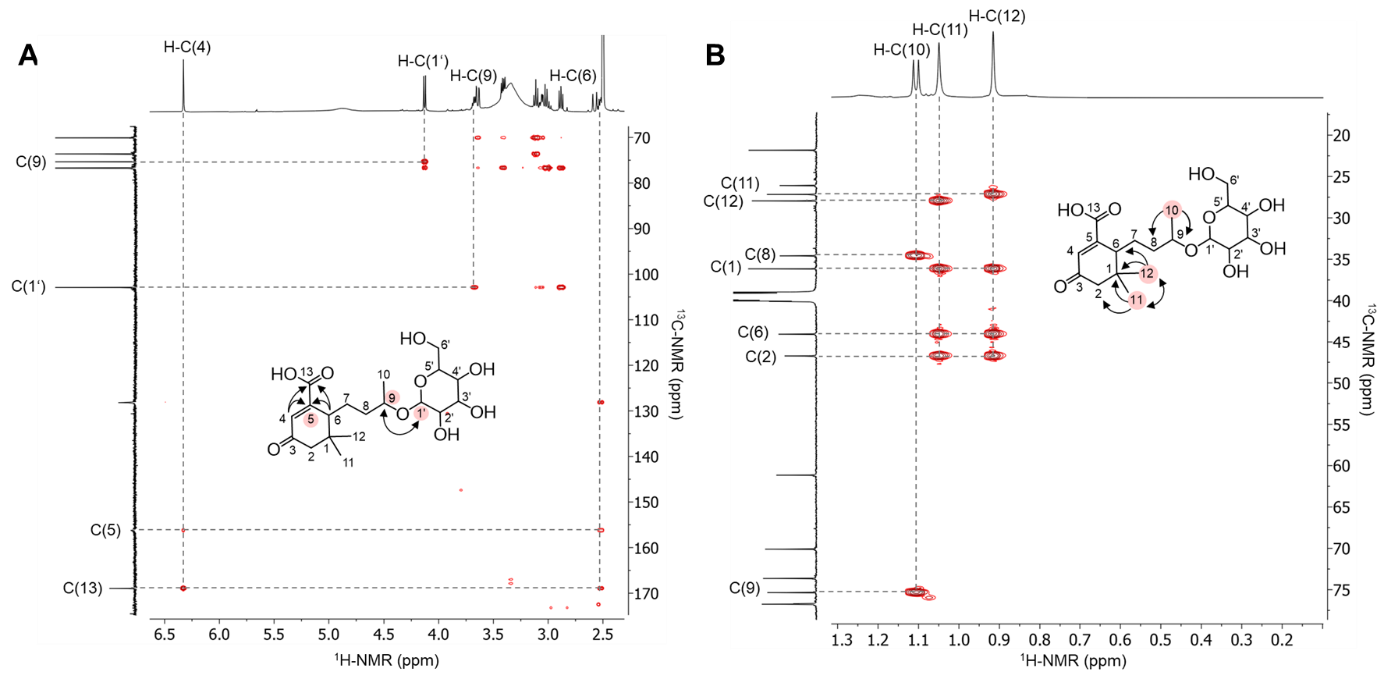


**Figure S9.** HMBC spectra (500/126 MHz, DMSO-d_6_, 300 K) of 5-carboxyblumenol C glucoside (**45**) illustrating (A) the position of the hexose and carboxy group and (B) the position of the methyl groups.

**
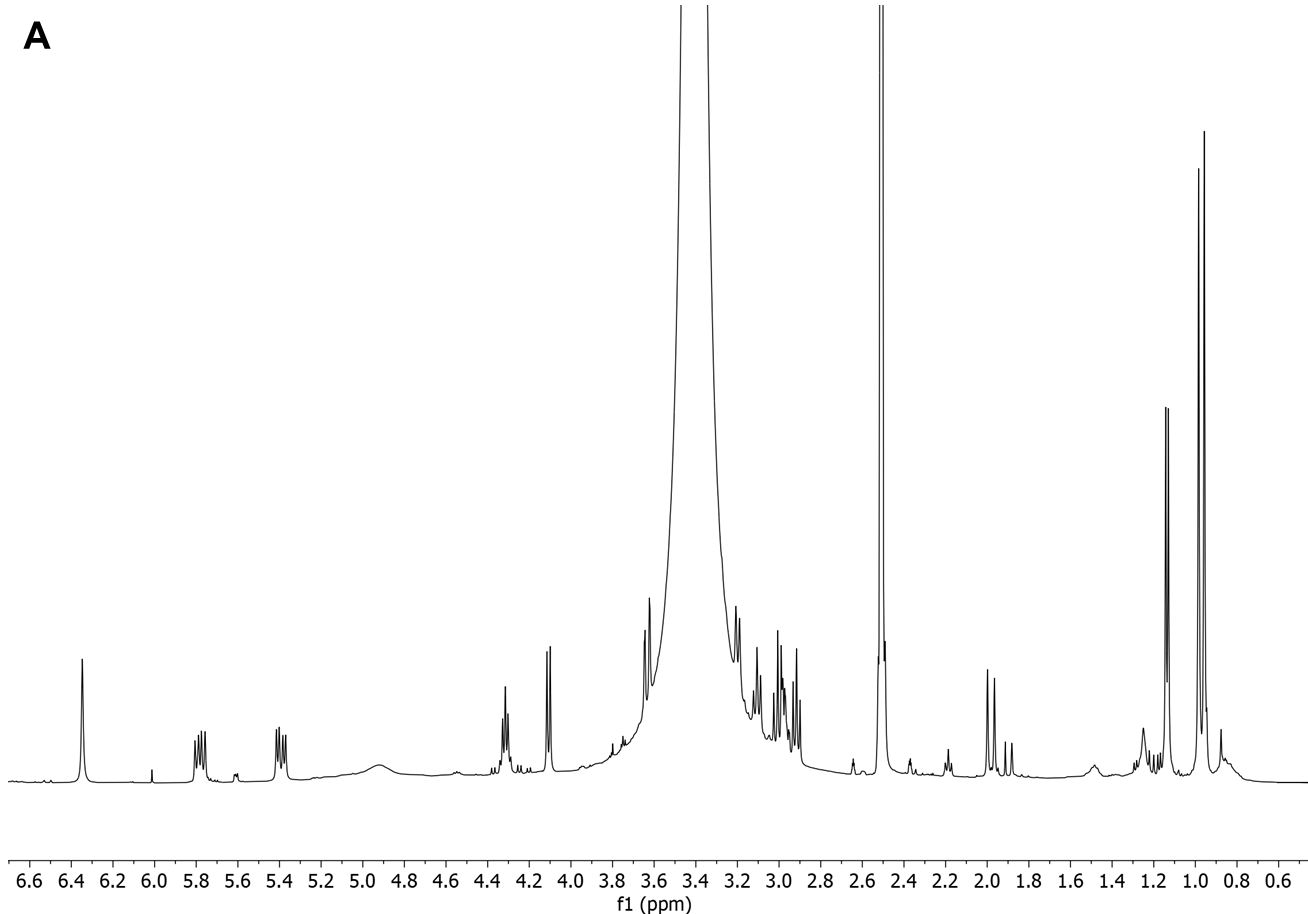
**


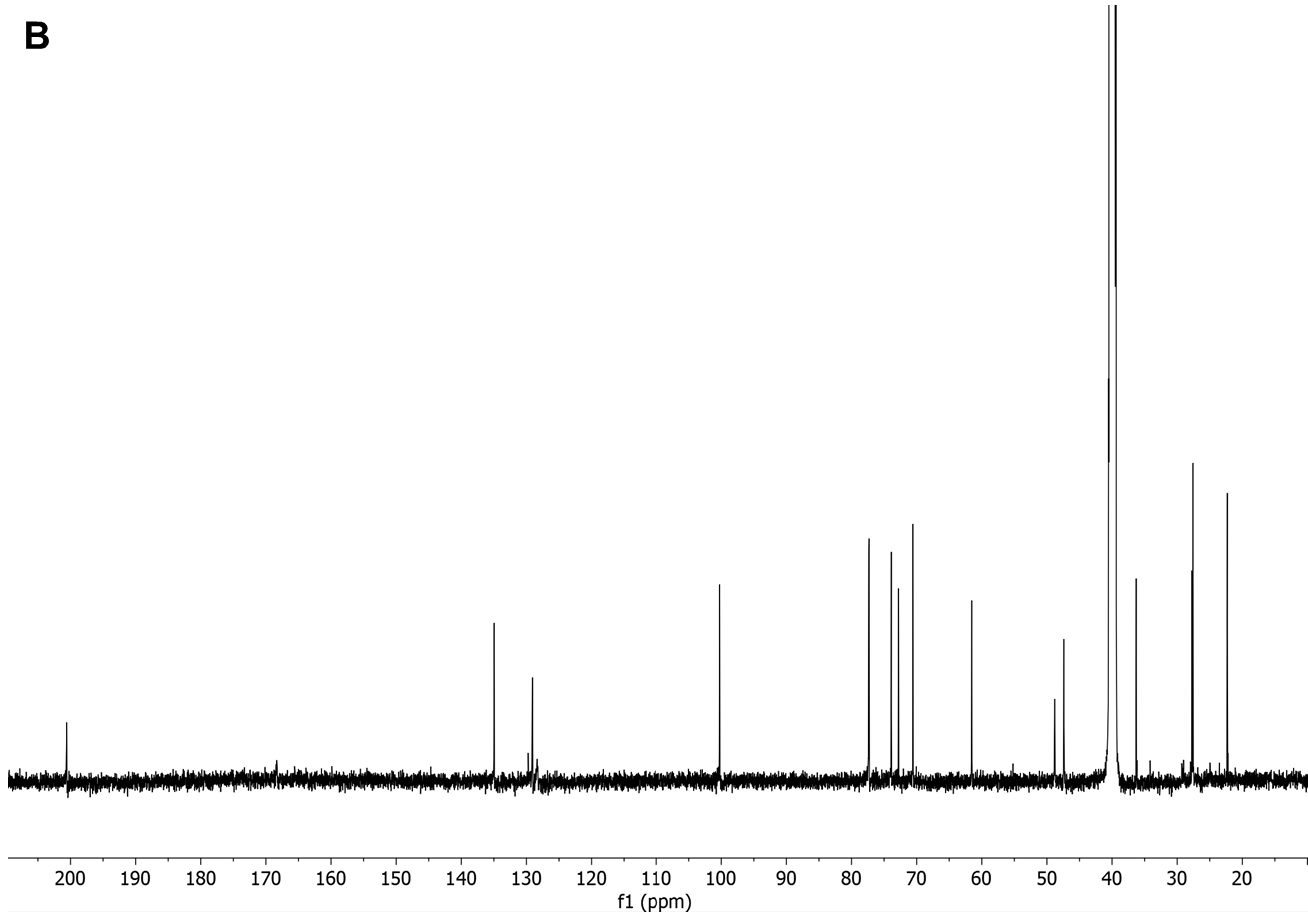


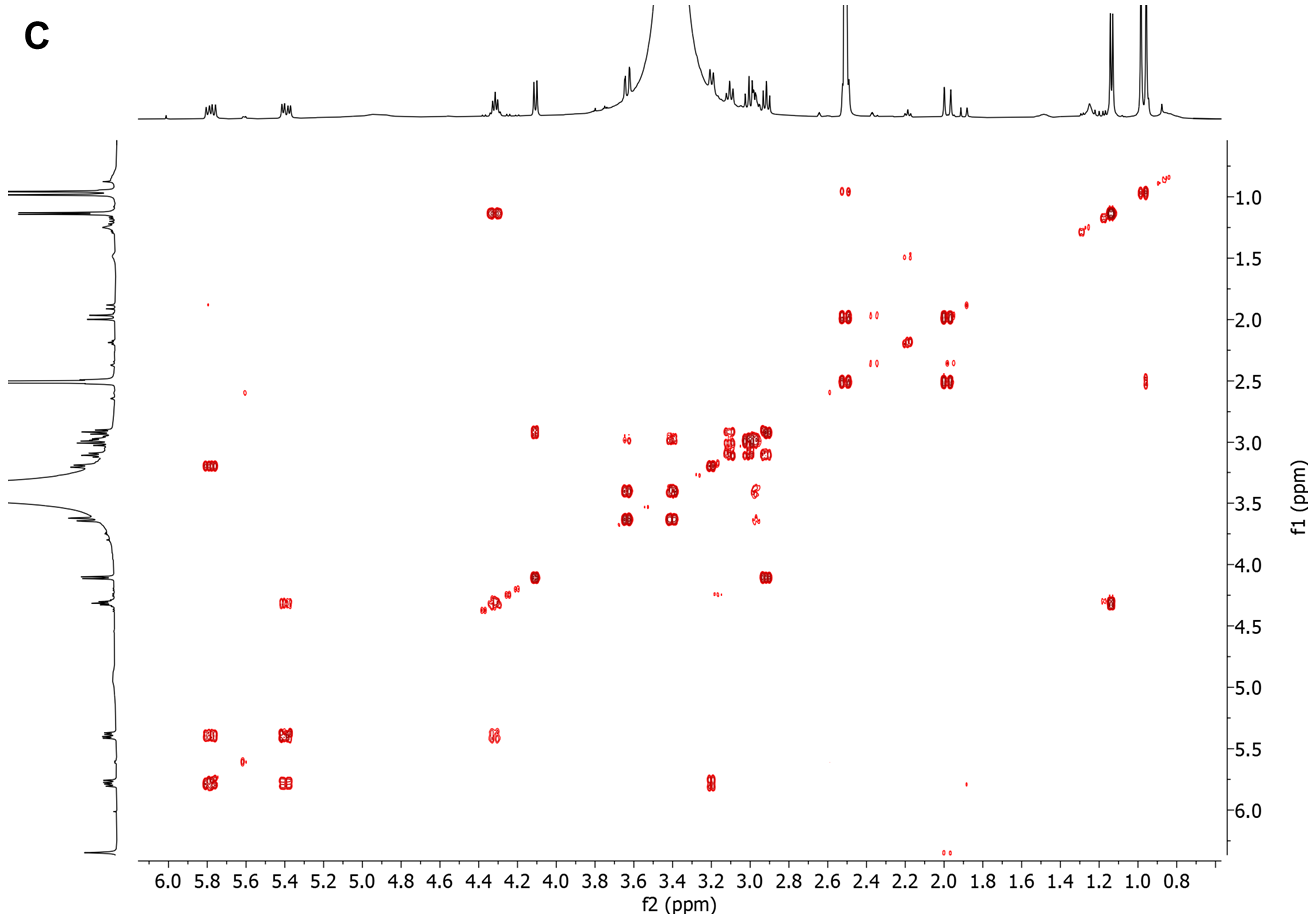


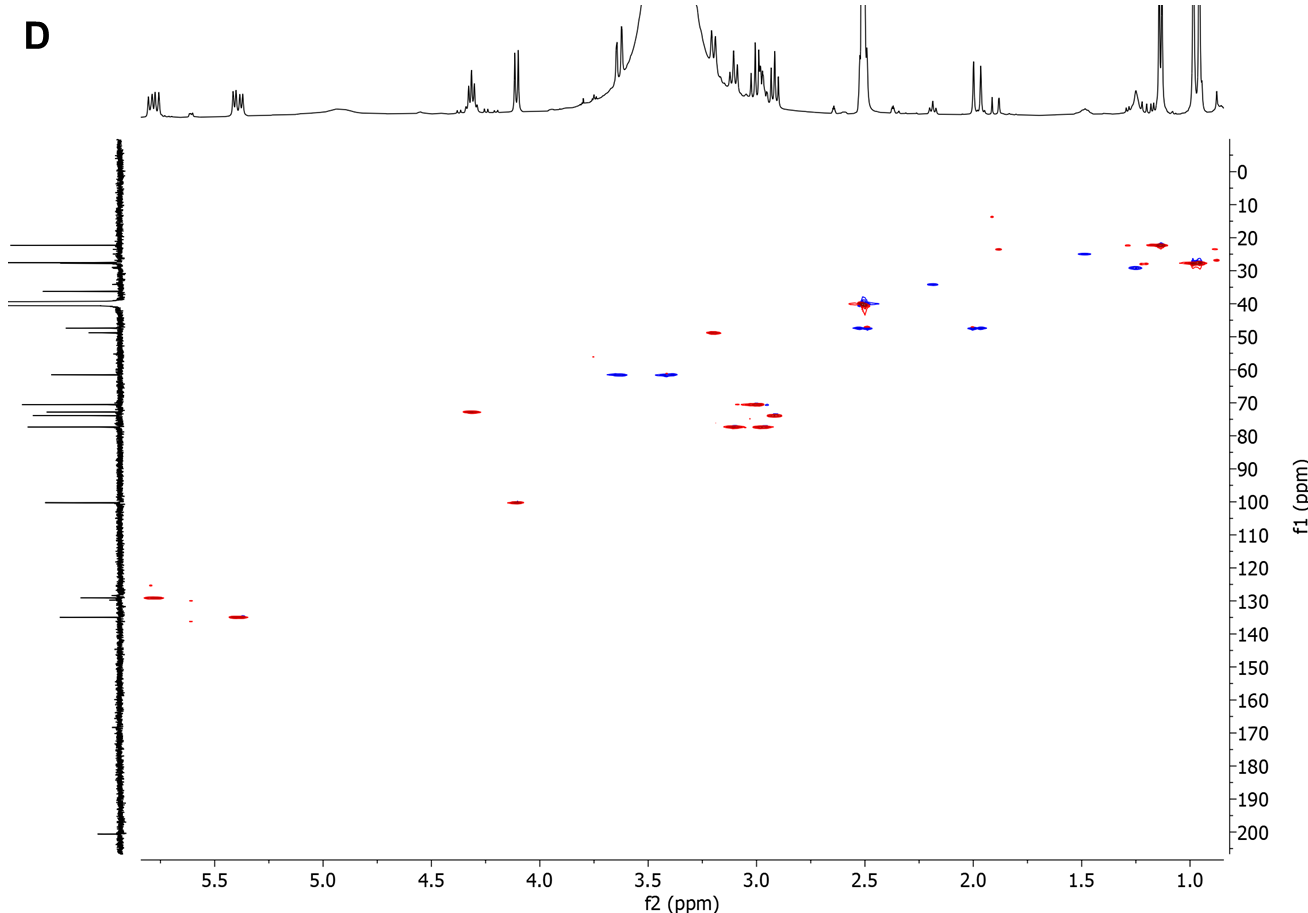

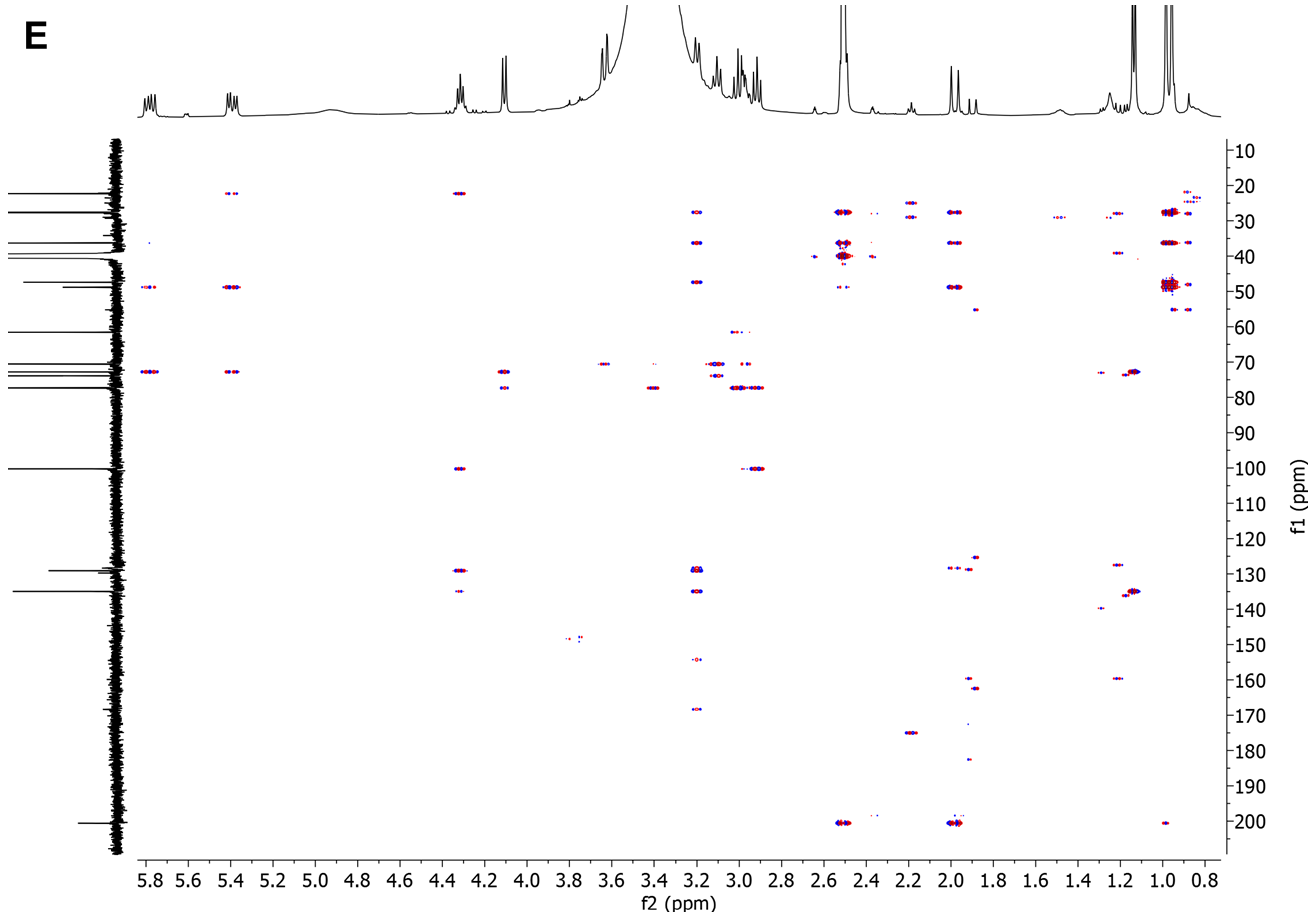


**Figure S10.** (A) ^1^H-, (B) ^13^C-NMR, (C) COSY, (D) HSQC and (E) HMBC spectra (500/126 MHz, DMSO-d_6_, 300 K) of 5-carboxydidehydroblumenol C 9-O-glucoside (**46**).


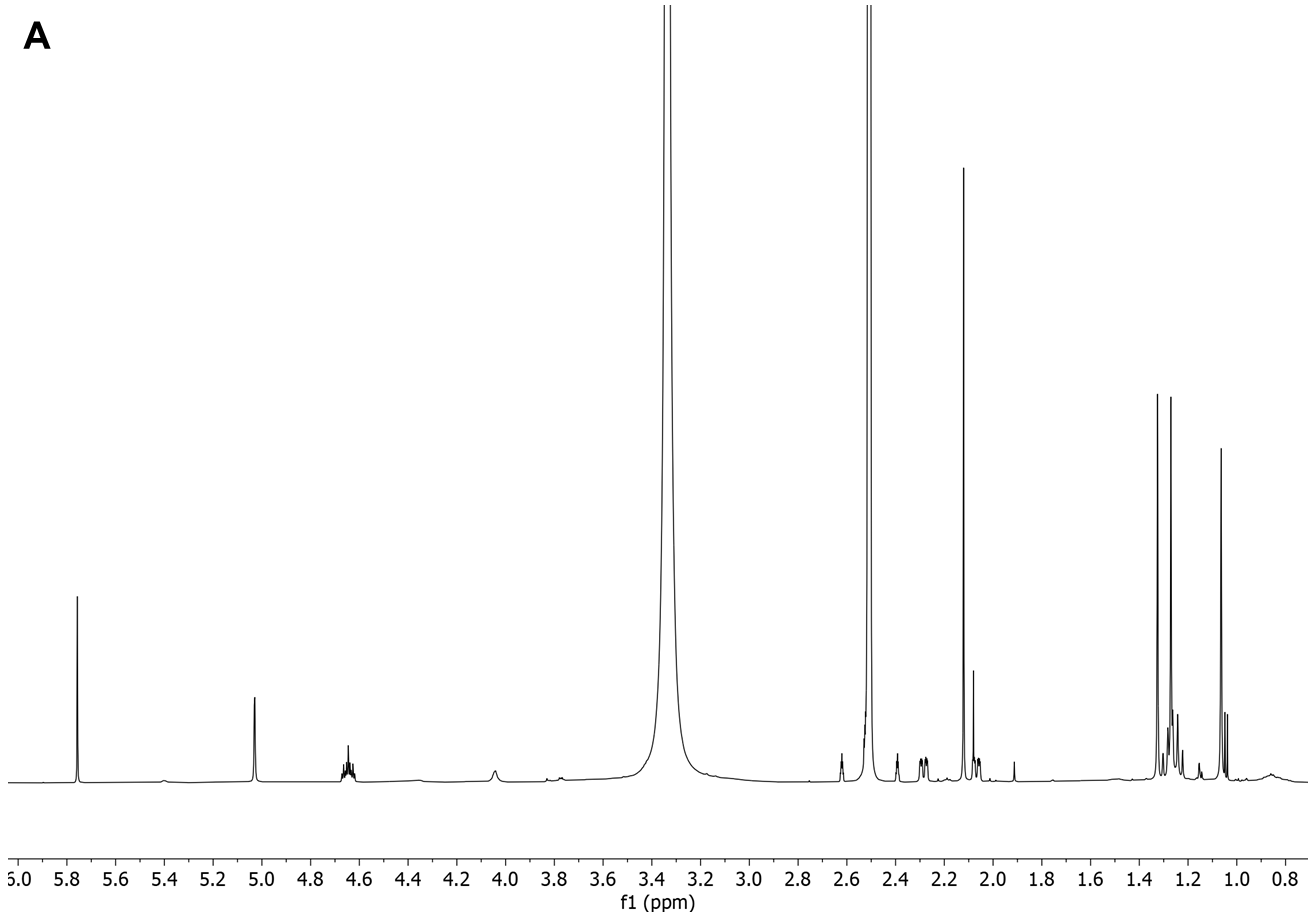


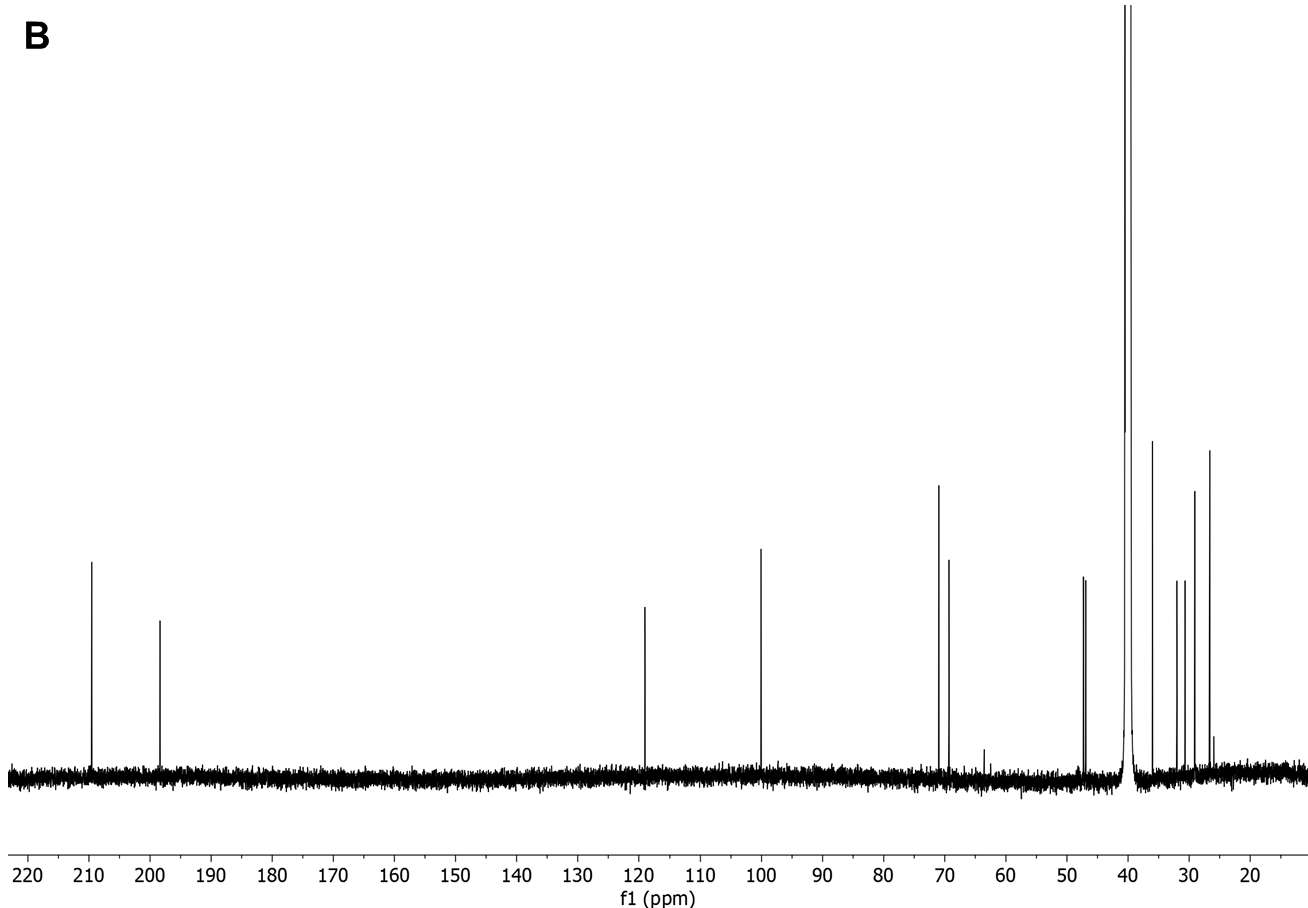


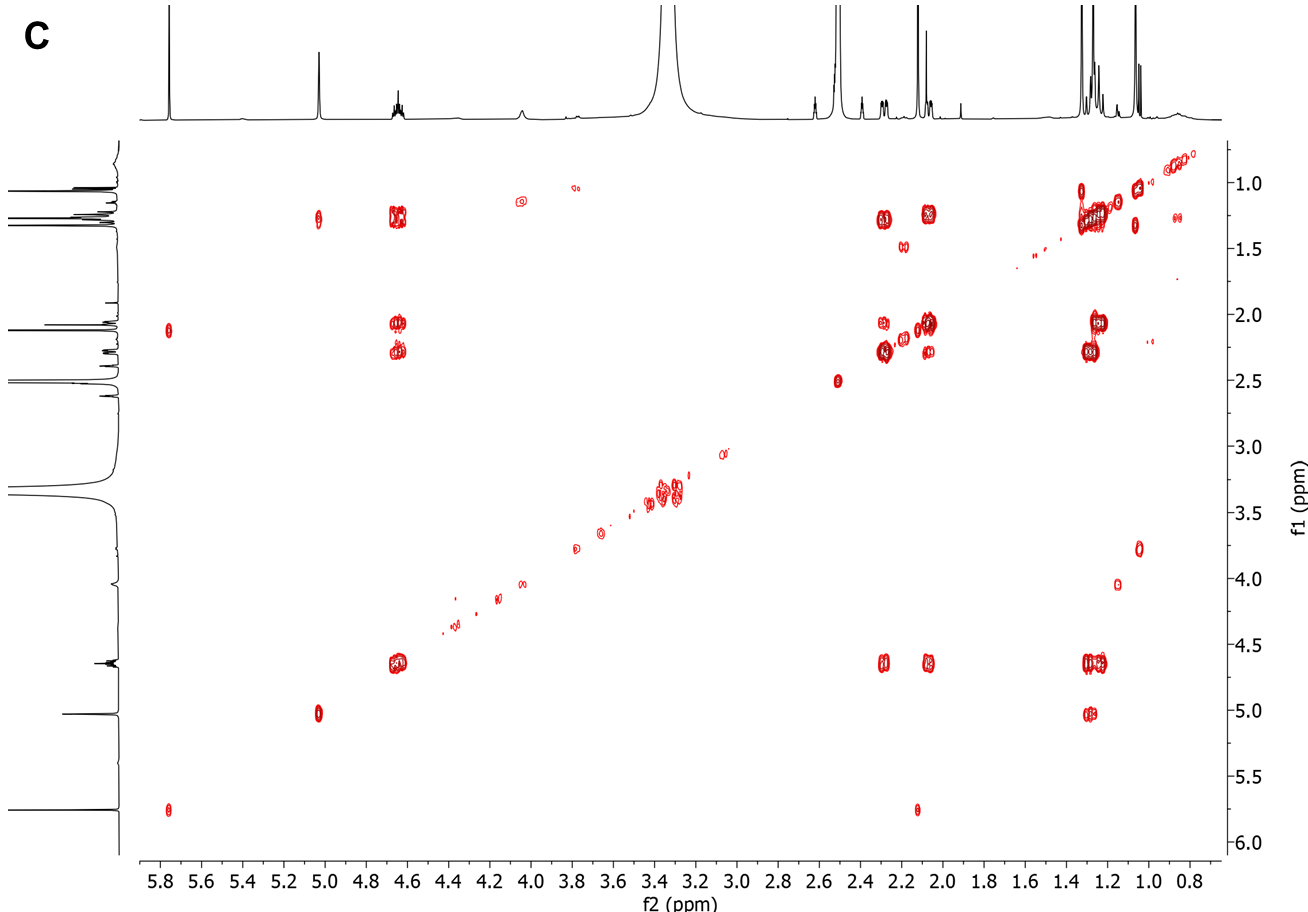


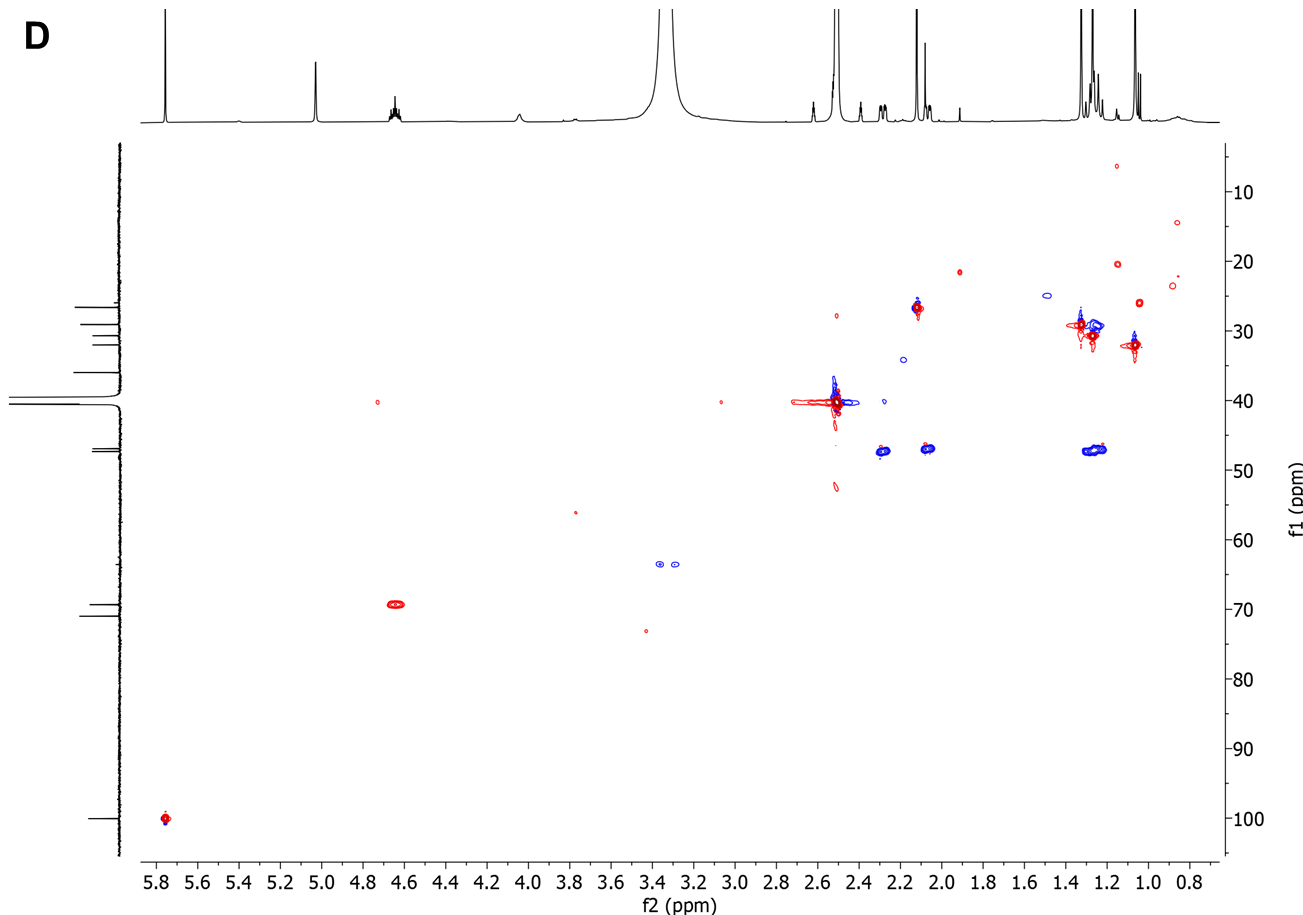


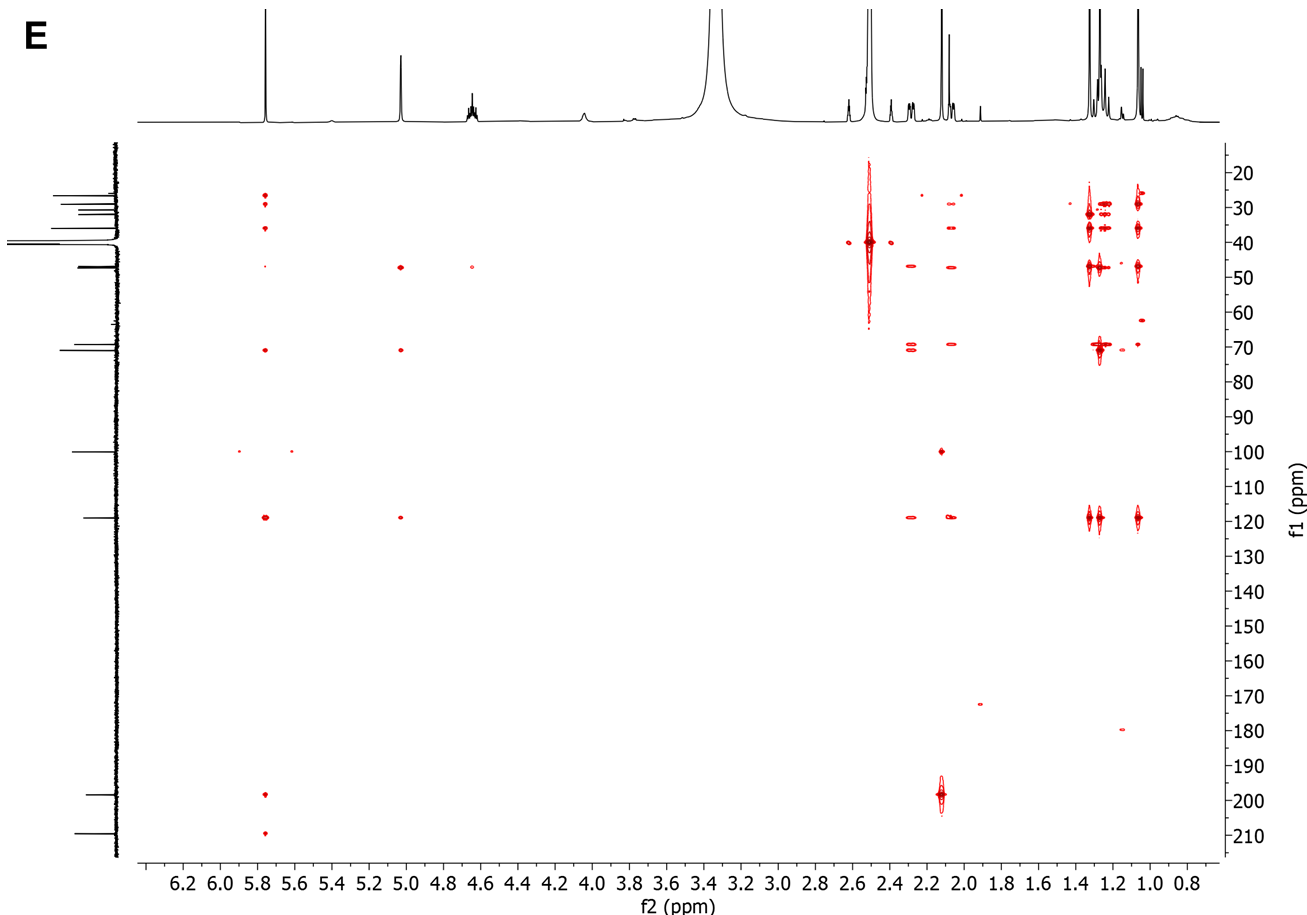


**Figure S11.** (A) ^1^H-, (B) ^13^C-NMR, (C) COSY, (D) HSQC and (E) HMBC spectra (500/126 MHz, DMSO-d_6_, 300 K) of grasshopper ketone-3-sulfate (**47**).


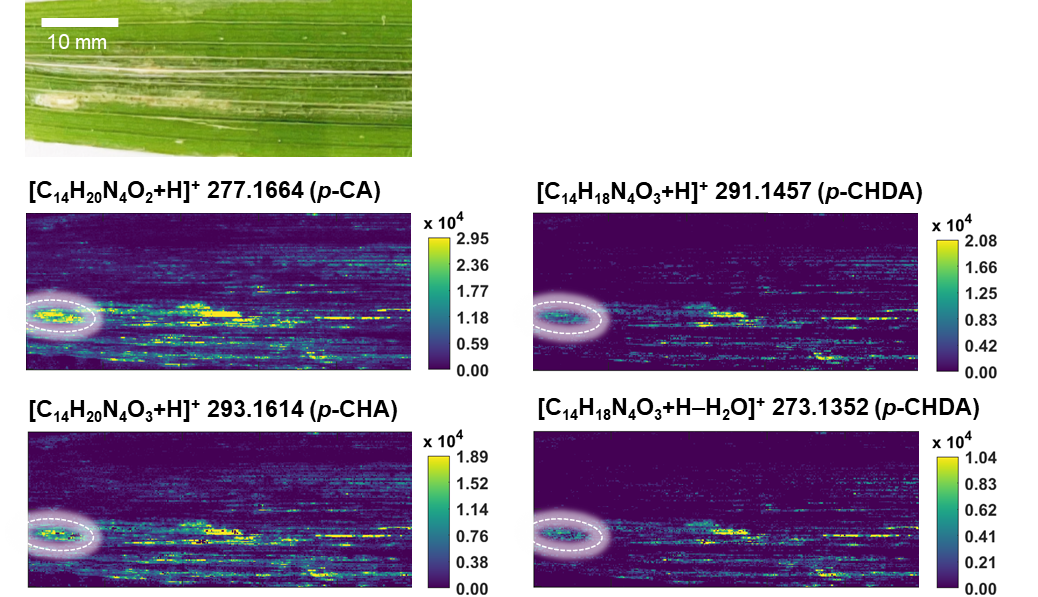


**Figure S12.** Optical image and DESI-MSI spectra of barley leaves with symptoms of spot blotch. The relative intensities of marker compounds p-CA (**31**), p-CHA (**32**), and p-CHDA (**33**) were higher in the symptomatic areas.


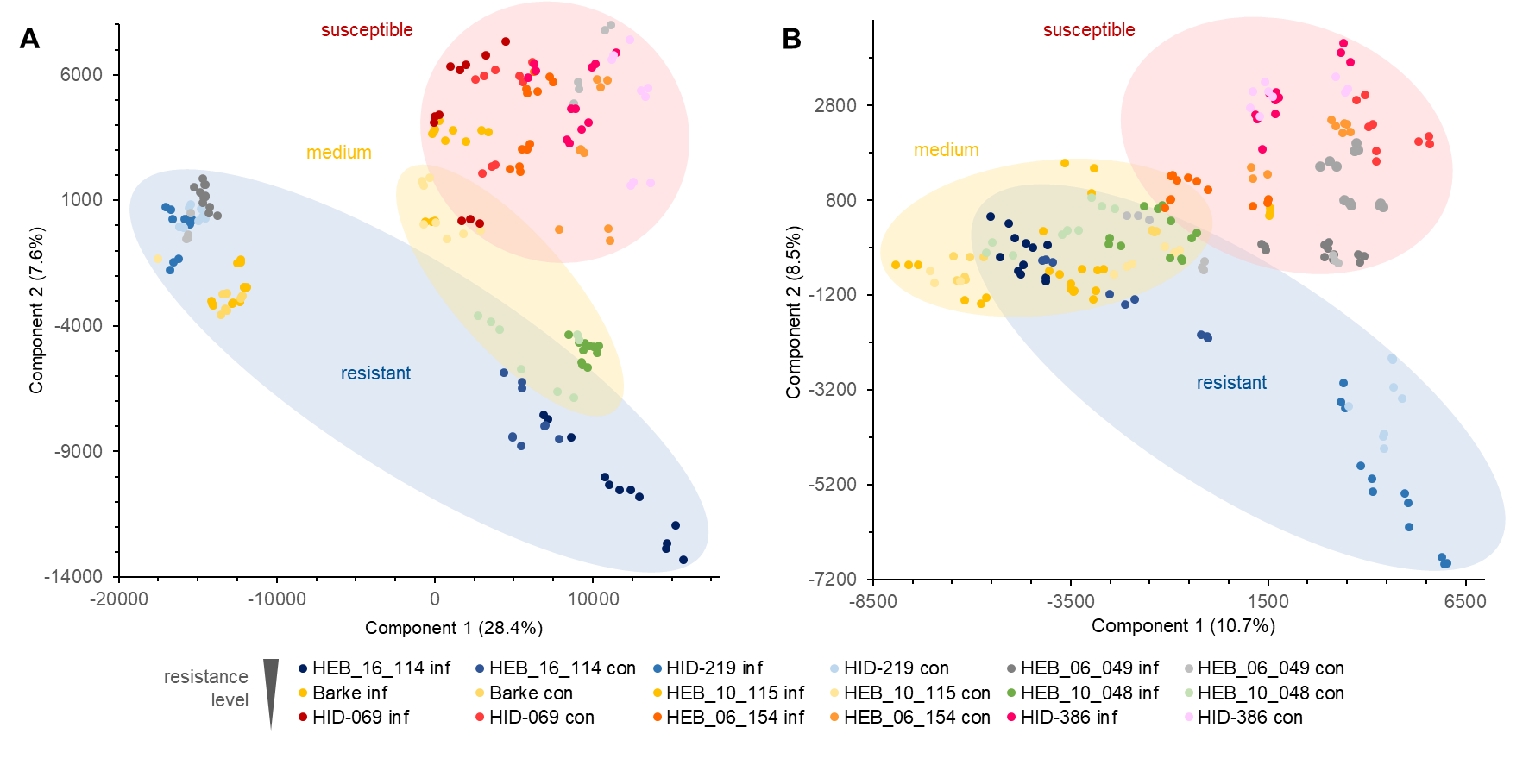


**Figure S13.** PCA score plots of different resistant and susceptible barley genotypes infected with B. sorokiniana in (A) ESI^+^ and (B) ESI^–^. Features were filtered by an ANOVA p-value ≤ 0.05 and a fold-change ≥ 2.


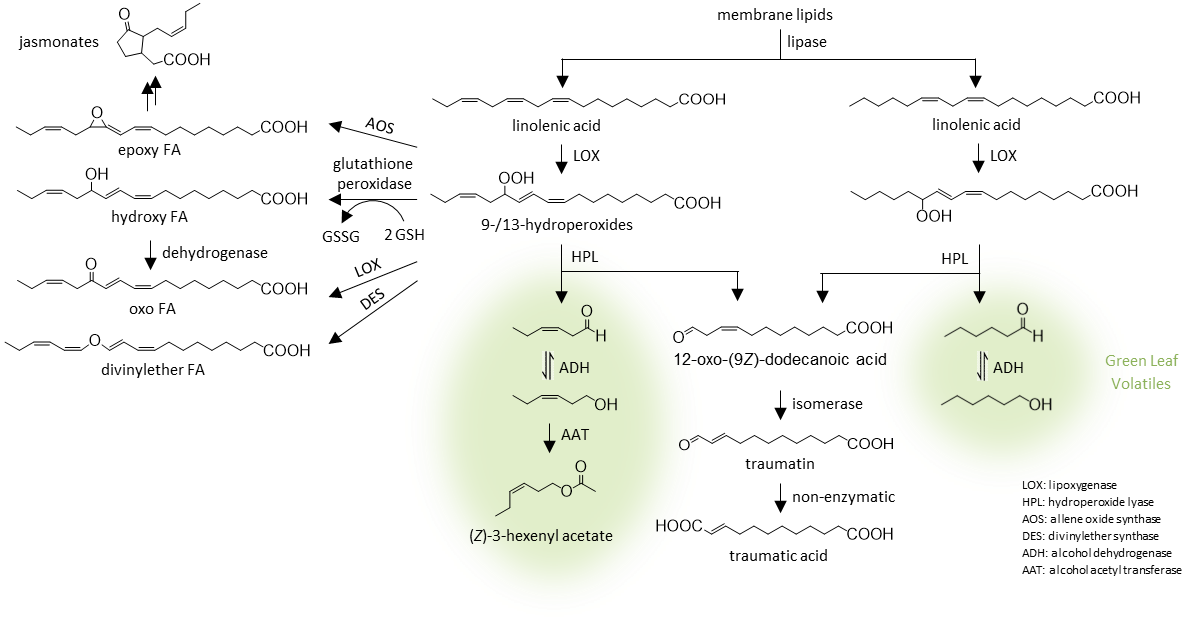


**Figure S14.** In the lipoxygenase pathway unsaturated fatty acids are oxidized into various oxylipins and green leaf volatiles.

## References

Giavalisco, P., Li, Y., Matthes, A., Eckhardt, A., Hubberten, H. M., Hesse, H., Segu, S., Hummel, J., Köhl, K., Willmitzer, L. (2011). Elemental formula annotation of polar and lipophilic metabolites using ^13^C, ^15^N and ^34^S isotope labelling, in combination with high-resolution mass spectrometry. *The Plant Journal, 68*(2), 364-376.
